# Supplementary material for: Novel Isoniazid-Carborane Hybrids Active In Vitro against Mycobacterium tuberculosis
Source: Pharmaceuticals (Basel). 2020 Dec 15;13(12):465. doi: 10.3390/ph13120465 (PMC7765321; doi:10.3390/ph13120465)
Supplement: Supplementary file 1 [file pharmaceuticals-13-00465-s001.pdf]

Supplementary Materials

Novel Isoniazid-Carborane Hybrids Active *in vitro* Against *Mycobacterium tuberculosis*

Daria Różycka[1], Małgorzata Korycka-Machała[1], Anna Żaczek[2], Jarosław Dziadek[1], Dorota Gurda[3], Marta Orlicka-Płocka[3], Eliza Wyszko[3], Katarzyna Biniek-Antosiak[3], Wojciech Rypniewski[3], Agnieszka B. Olejniczak[1,*]

1 Institute of Medical Biology, Polish Academy of Sciences, 106 Lodowa St., 93-232 Lodz, Poland; drozycka@cbm.pan.pl (D.R.); mkorycka@cbm.pan.pl (M.K.M.); jdziadek@cbm.pan.pl (J.D.); aolejniczak@cbm.pan.pl (A.B.O.)

2 Institute of Medical Sciences, Medical College, University of Rzeszow, 2A Kopisto Avenue, 35-959 Rzeszow, Poland; ania.zaczek@yahoo.pl (A.Z.)

3 Institute of Bioorganic Chemistry, Polish Academy of Sciences, 12/14 Z. Noskowskiego St., 61-704 Poznan, Poland; d_gurda@ibch.poznan.pl (D.G.); mplocka@ibch.poznan.pl (M.O.P.); wyszkoe@ibch.poznan.pl (E.W.), kbiniek@ibch.poznan.pl (K.B.A.); wojtekr@ibch.poznan.pl (W. R.)

* Correspondence: aolejniczak@cbm.pan.pl (A. B. O.); Tel.: +48-42-272-36-37

Table of contents

**Figure S1**. ^{1}H NMR spectrum of compound **3**	4
**Figure S2**. ^{13}C NMR spectrum of compound **3**	5
**Figure S3**. ^{11}B NMR {H BB} spectrum of compound **3**	6
**Figure S4**. IR spectrum of compound **3**	7
**Figure S5**. MS-APCI spectrum of compound **3**	8
**Figure S6**. FT-IR spectrum of compound **8**	9
**Figure S7**. ^{13}C NMR spectrum of compound **8**	10
**Figure S8**. ^{11}B NMR {H BB} spectrum of compound **8**	11
**Figure S9.** IR spectrum of compound **8**	12
**Figure S10.** APCI-MS spectrum of compound **8**	13
**Figure S11**. HPLC analysis of compound **8**	14
**Figure S12**. ^{1}H NMR spectrum of compound **9**	15
**Figure S13**. ^{13}C NMR spectrum of compound **9**	16
**Figure S14**. ^{11}B NMR {H BB} spectrum of compound **9**	17
**Figure S15**. IR spectrum of compound **9**	18
**Figure S16**. APCI-MS spectrum of compound **9**	19
**Figure S17**. HPLC analysis of compound **9**	20
**Figure S18**. ^{1}H NMR spectrum of compound **10**	21
**Figure S19**. ^{13}C NMR spectrum of compound **10**	22
**Figure S20**. ^{11}B NMR {H BB} spectrum of compound **10**	23
**Figure S21.** IR spectrum of compound **10**	24
**Figure S22**. APCI-MS spectrum of compound **10**	25
**Figure S23**. HPLC analysis of compound **10**	26
**Figure S24**. ^{1}H NMR spectrum of compound **14**	27
**Figure S25**. ^{13}C NMR spectrum of compound **14**	28
**Figure S26**. ^{11}B NMR {^{1}H BB} spectrum of compound **14**	29
**Figure S27**. IR spectrum of compound **14**	30
**Figure S28**. MS spectrum of compound **14**	31
**Figure S29**. HPLC analysis of compound **14**	32
**Figure S30**. ^{1}H NMR spectrum of compound **15**	33
**Figure S31**. ^{13}C NMR spectrum of compound **15**	34
**Figure S32**. ^{11}B NMR {^{1}H BB} spectrum of compound **15**	35
**Figure S33**. IR spectrum of compound **15**	36
**Figure S34**. MS spectrum of compound **15**	37
**Figure S35**. HPLC analysis of compound **15**	38
**Figure S36**. ^{1}H NMR spectrum of compound **16**	39
**Figure S37**. ^{13}C NMR spectrum of compound **16**	40
**Figure S38**. ^{11}B NMR {^{1}H BB} spectrum of compound **16**	41
**Figure S39**. IR spectrum of compound **16**	42
**Figure S40**. MS spectrum of compound **16**	43
**Figure S41**. HPLC analysis of compound **16**	44
**Figure S42**. ^{1}H NMR spectrum of compound **20**	45
**Figure S43**. ^{13}C NMR spectrum of compound **20**	46
**Figure S44**. ^{11}B NMR {^{1}H BB} spectrum of compound **20**	47
**Figure S45**. IR spectrum of compound **20**	48
**Figure S46**. MS spectrum of compound **20**	49
**Figure S47**. HPLC analysis of compound **20**	50
**Figure S48**. ^{1}H NMR spectrum of compound **21**	51
**Figure S49**. ^{13}C NMR spectrum of compound **21**	52

Figure S50. ^{11}B NMR {^{1}H BB} spectrum of compound **21** 53
Figure S51. IR spectrum of compound **21** 54
Figure S52. MS spectrum of compound **21** 55
Figure S53. HPLC analysis of compound **21** 56
Figure S54. ^{1}H NMR spectrum of compound **22** 57
Figure S55. ^{13}C NMR spectrum of compound **22** 58
Figure S56. ^{11}B NMR {^{1}H BB} spectrum of compound **22** 59
Figure S57. IR spectrum of compound **22** 60
Figure S58. MS spectrum of compound **22** 61
Figure S59. HPLC analysis of compound **22** 62
Figure S60. ^{1}H NMR spectrum of compound **23** 63
Figure S61. ^{13}C NMR spectrum of compound **23** 64
Figure S62. ^{11}B NMR {^{1}H BB} spectrum of compound **23** 65
Figure S63. IR spectrum of compound **23** 66
Figure S64. MS spectrum of compound **23** 67
Figure S65. HPLC analysis of compound **23** 68
Figure S66. ^{1}H NMR spectrum of compound **24** 69
Figure S67. ^{13}C NMR spectrum of compound **24** 70
Figure S68. ^{11}B NMR {^{1}H BB} spectrum of compound **24** 71
Figure S69. IR spectrum of compound **24** 72
Figure S70. MS spectrum of compound **24** 73
Figure S71. HPLC analysis of compound **24** 74
Figure S72. ^{1}H NMR spectrum of compound **25** 75
Figure S73. ^{13}C NMR spectrum of compound **25** 76
Figure S74. ^{11}B NMR {^{1}H BB} spectrum of compound **25** 77
Figure S75. IR spectrum of compound **25** 78
Figure S76. MS spectrum of compound **25** 79
Figure S77. HPLC analysis of compound **25** 80
Table 1S. Crystallographic data 81
General procedure for competitive trapping 82
Figure S78. The resulting trapped product *O*-isonicotinoylhydroxylamine (**26**) 83
Figure S79. MS spectrum of reaction of INH with TEMPO, and manganese catalyst 84
Figure S80. MS spectrum of reaction of **8** with TEMPO, and manganese catalyst 85
Figure S81. MS spectrum of reaction of **21** with TEMPO, and manganese catalyst 86
Figure S82. MS spectrum of reaction of **25** with TEMPO, and manganese catalyst 87
Figure S83. Confirmation of mutant construction by Southern Blotting analysis. 88

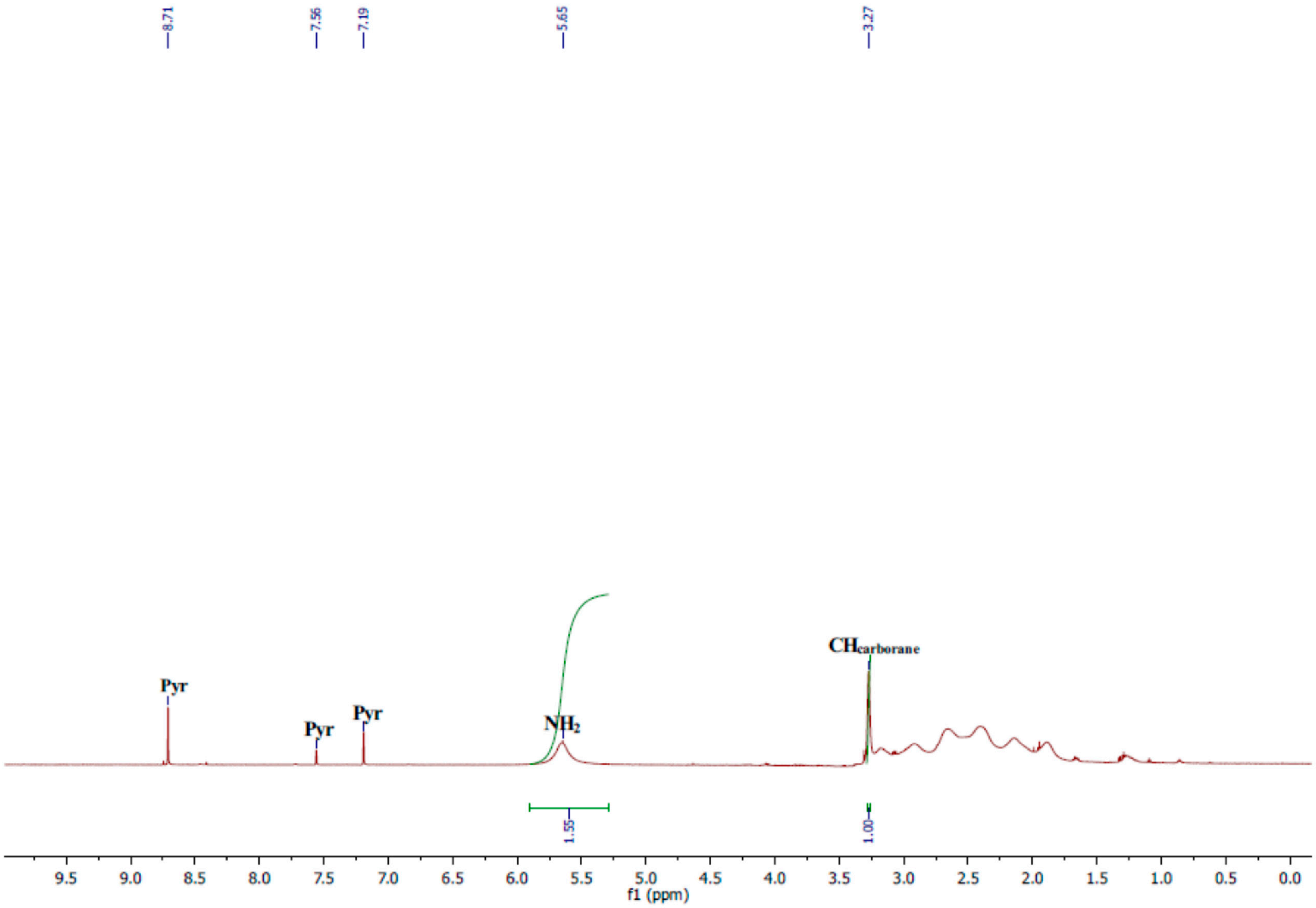

Figure S1. ^{1}H NMR spectrum of compound **3**.

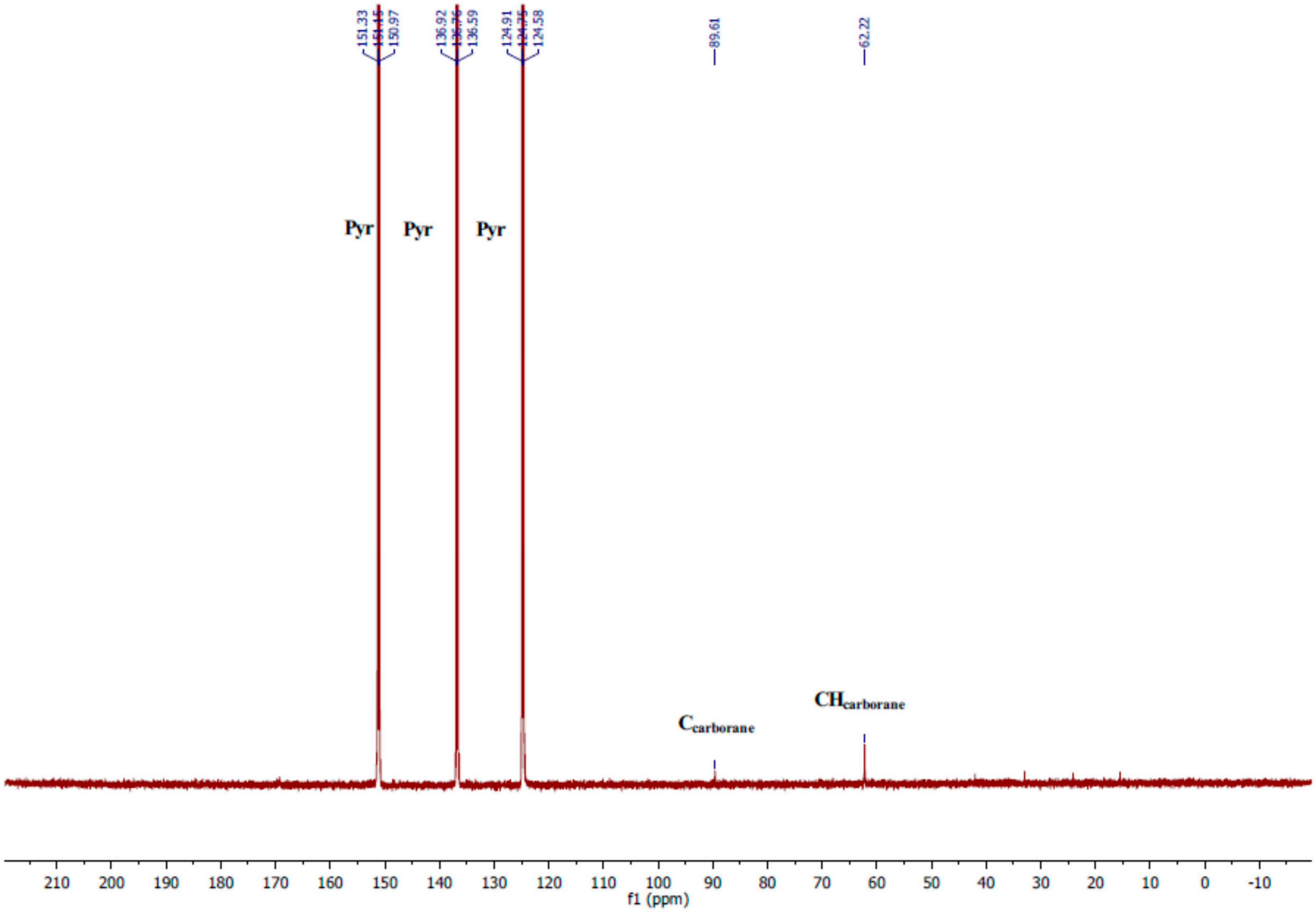

Figure S2. ^{13}C NMR spectrum of compound **3**.

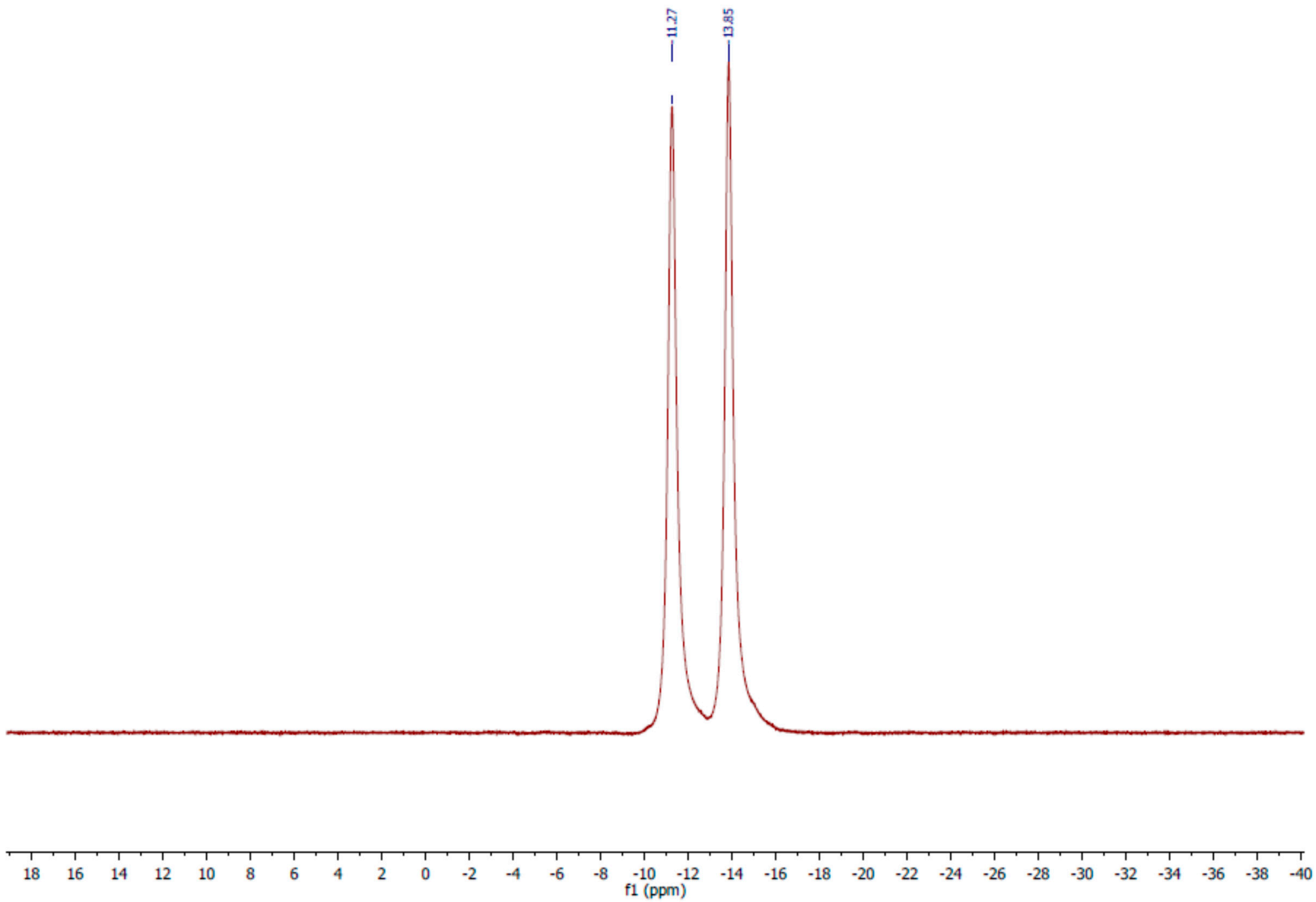

Figure S3. ^{11}B NMR {H BB} spectrum of compound **3**.

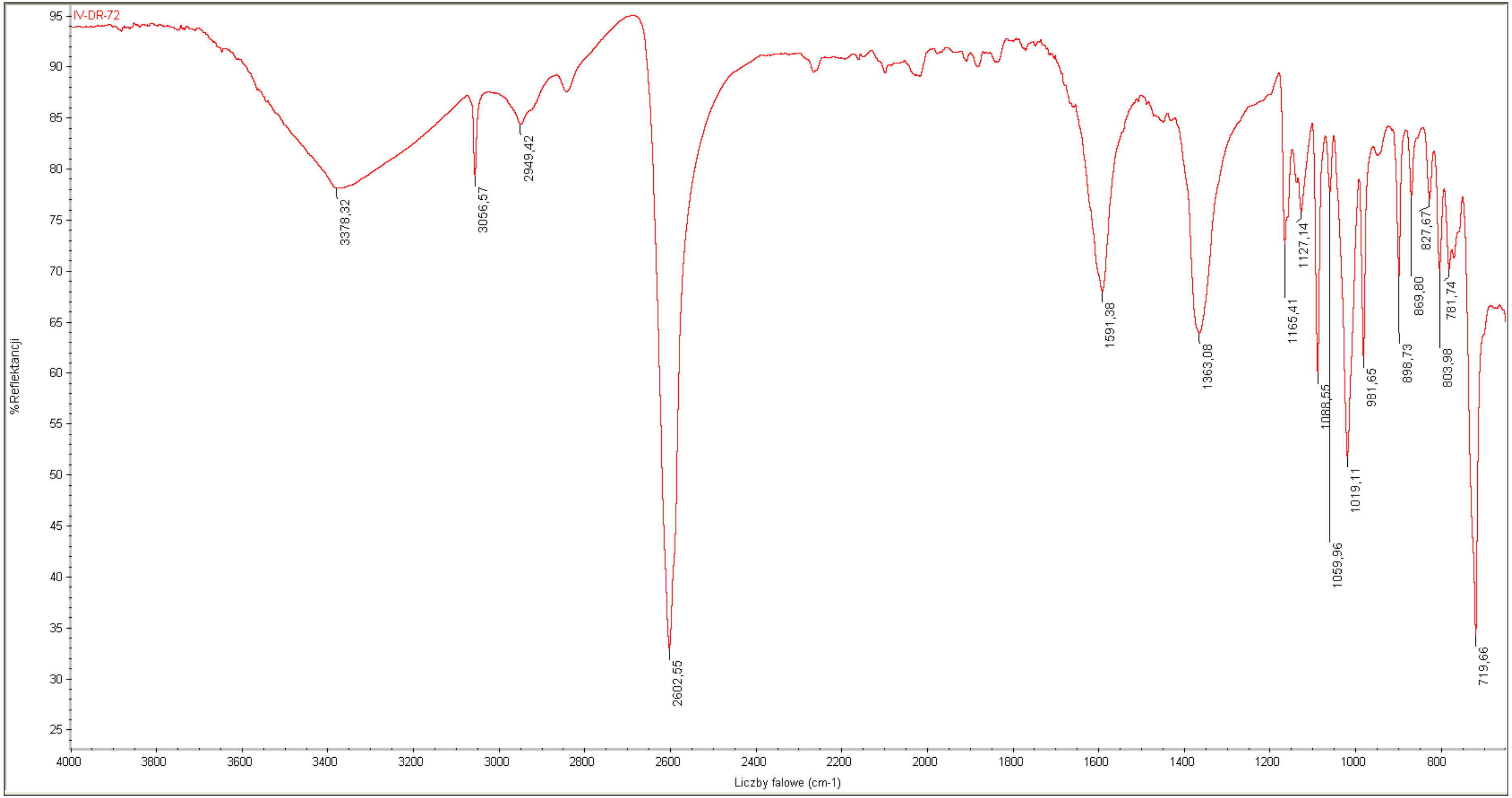

Figure S4. IR spectrum of compound **3**.

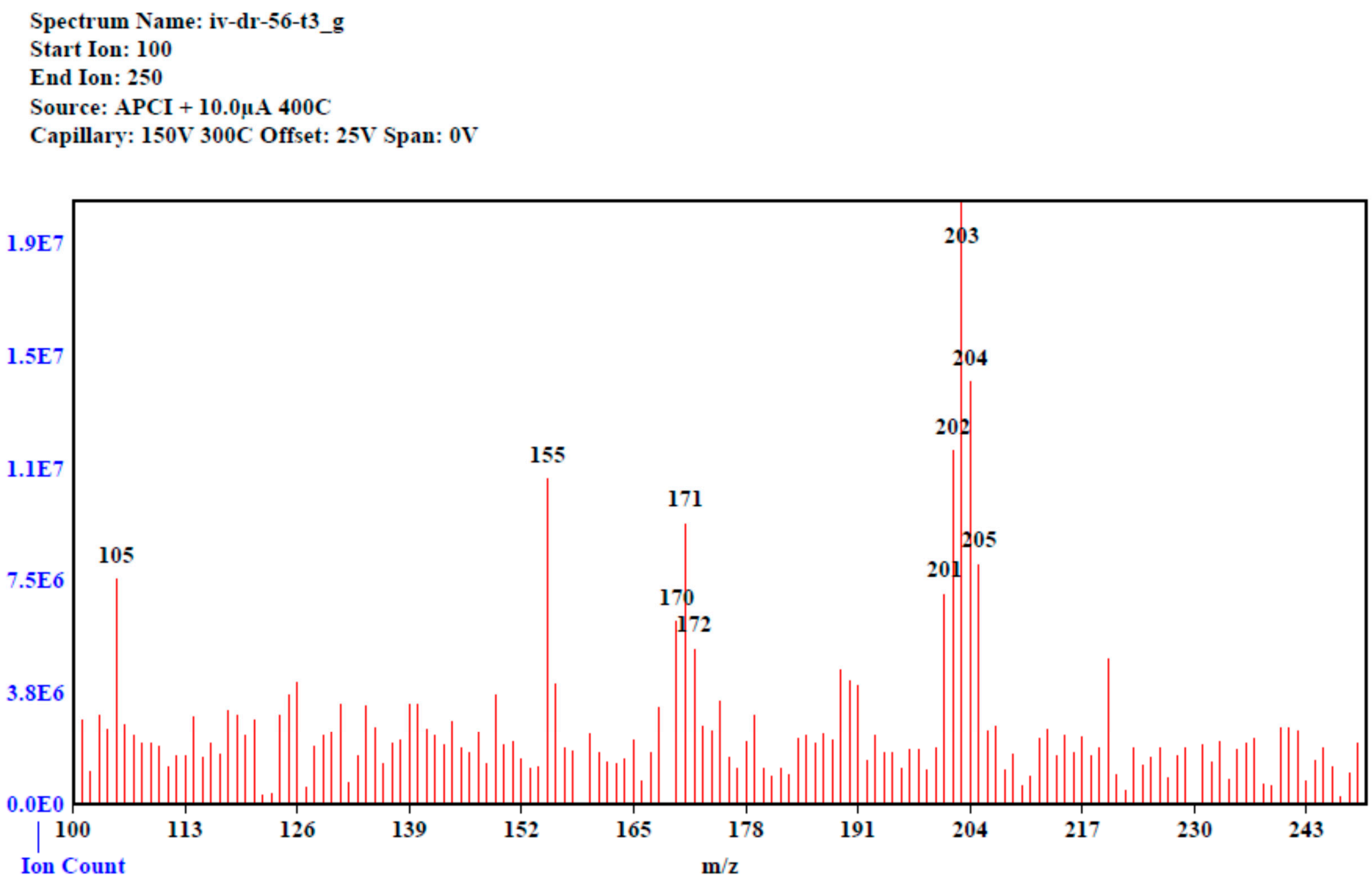

Figure S5. APCI-MS spectrum of compound **3**.

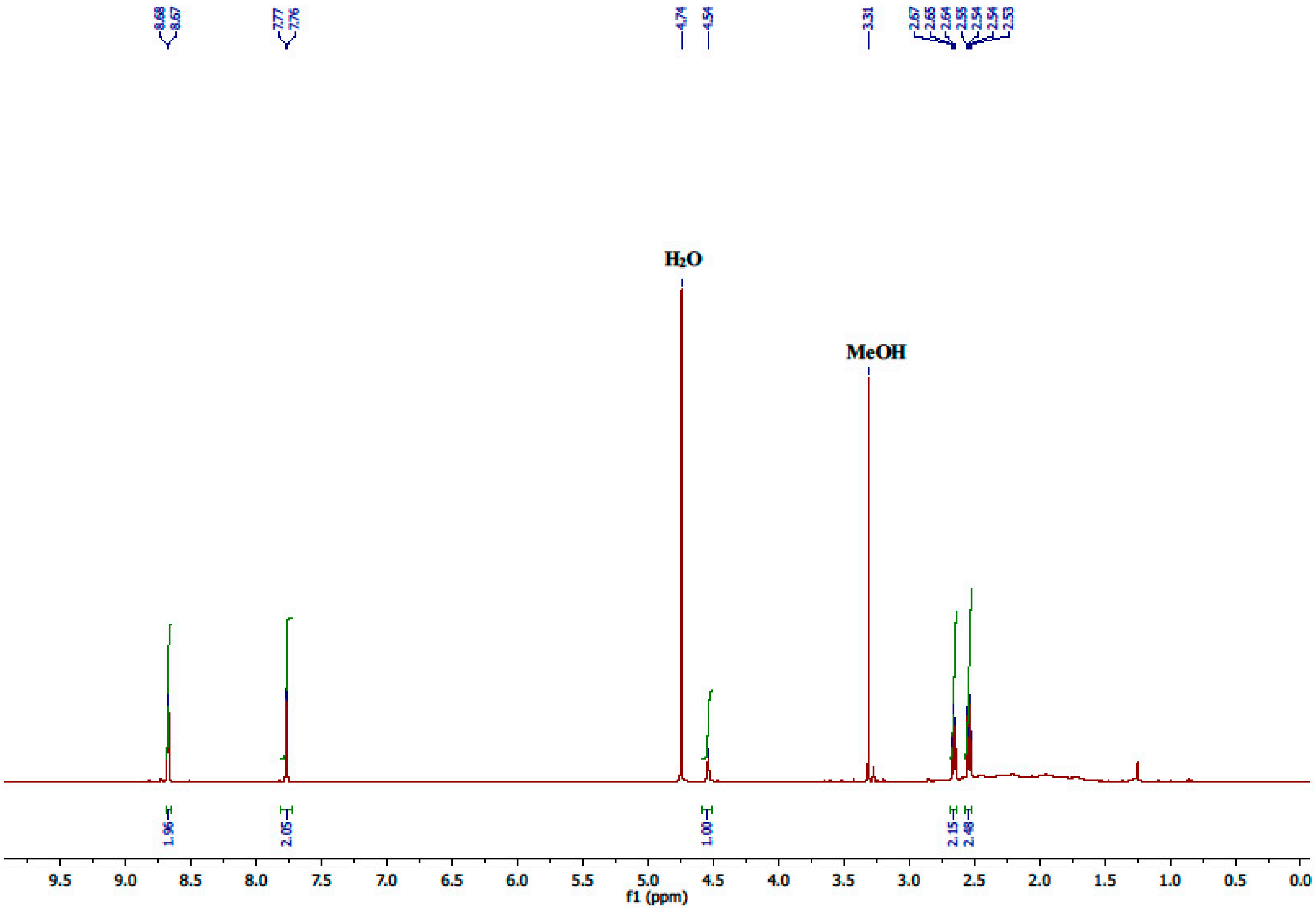

Figure S6. ^{1}H NMR spectrum of compound **8**.

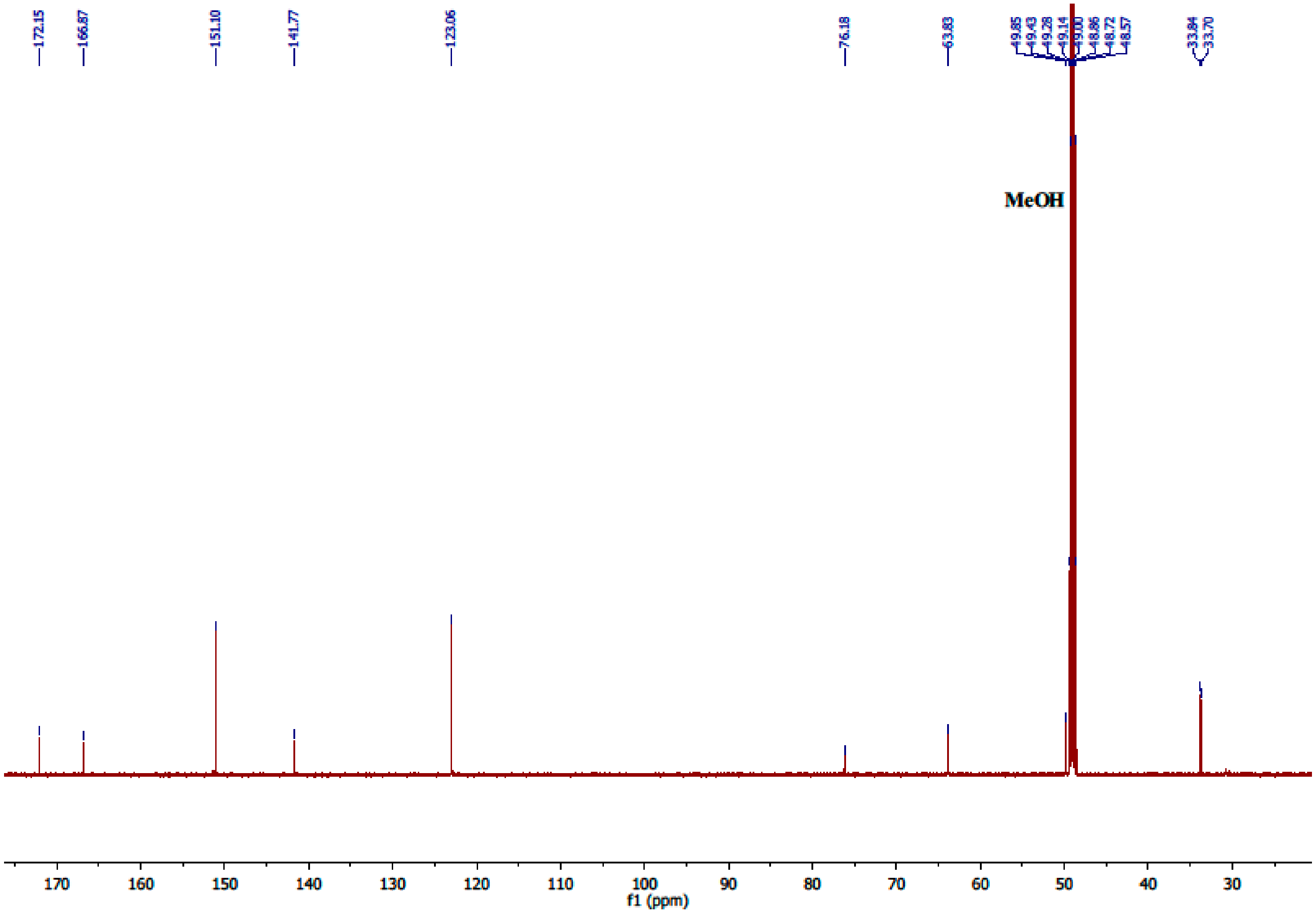

Figure S7. ^{13}C NMR spectrum of compound **8**.

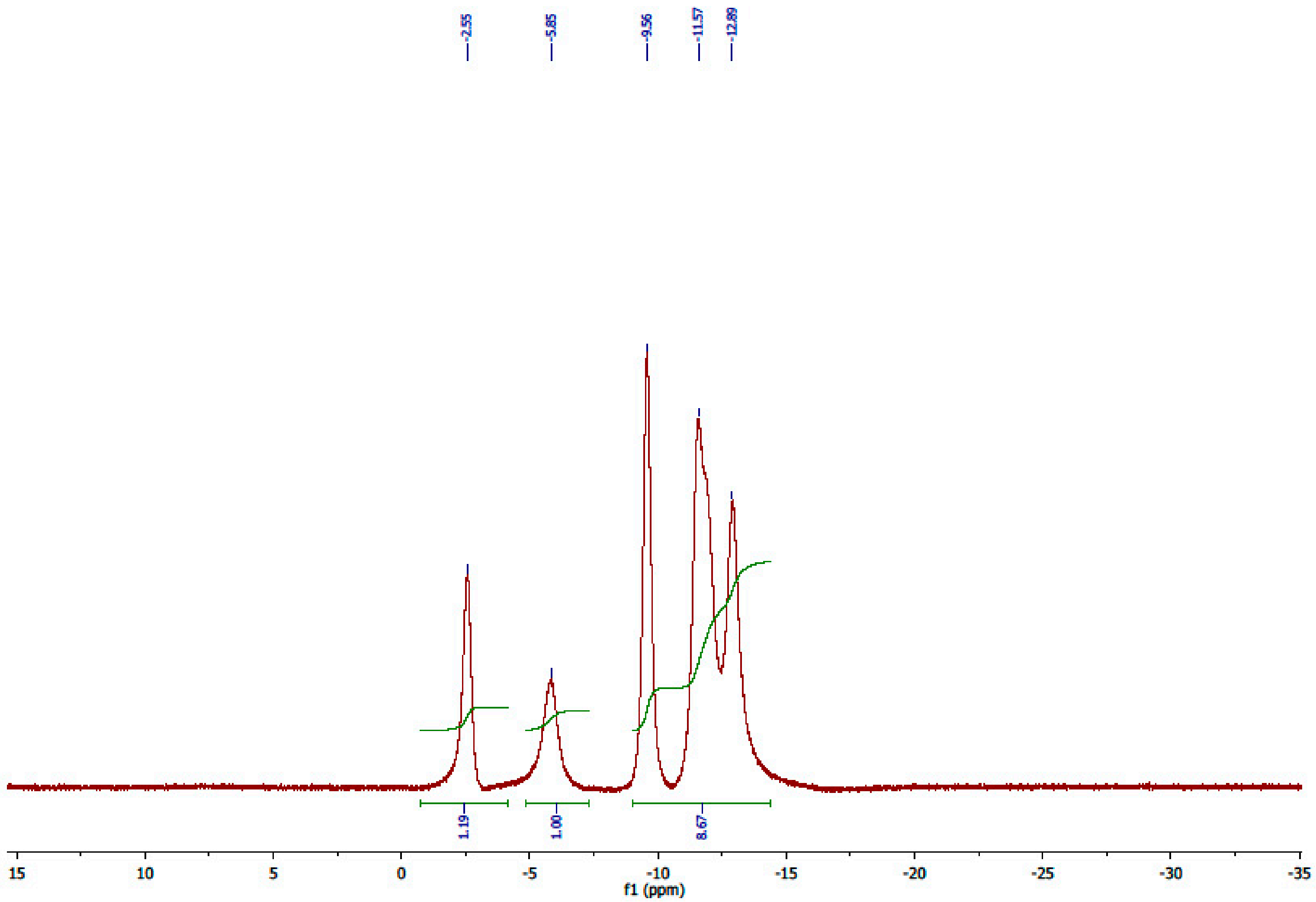

Figure S8. ^{11}B NMR {H BB} spectrum of compound **8**.

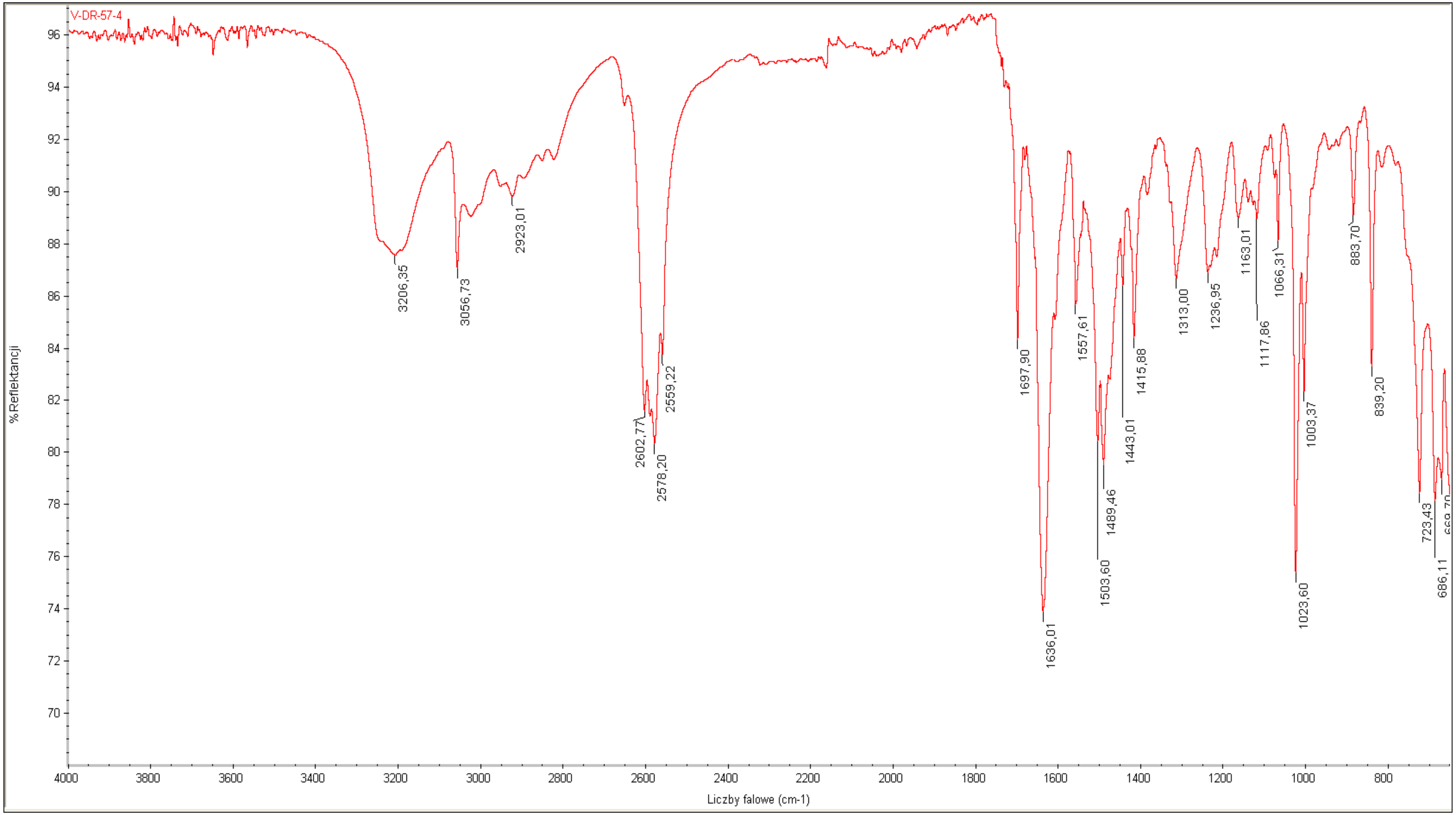

Figure S9. IR spectrum of compound **8**.

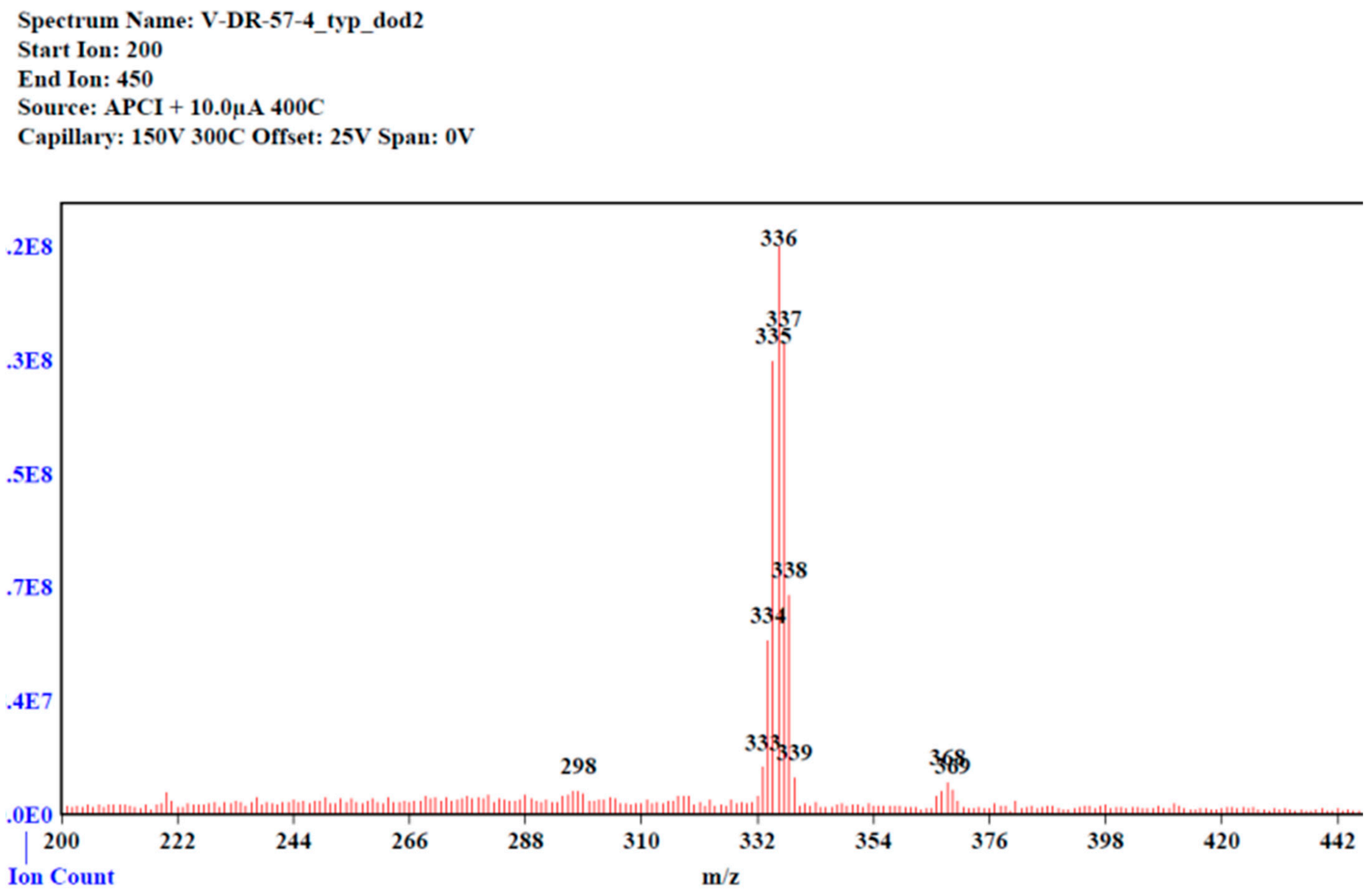

Figure S10. APCI-MS spectrum of compound **8**.

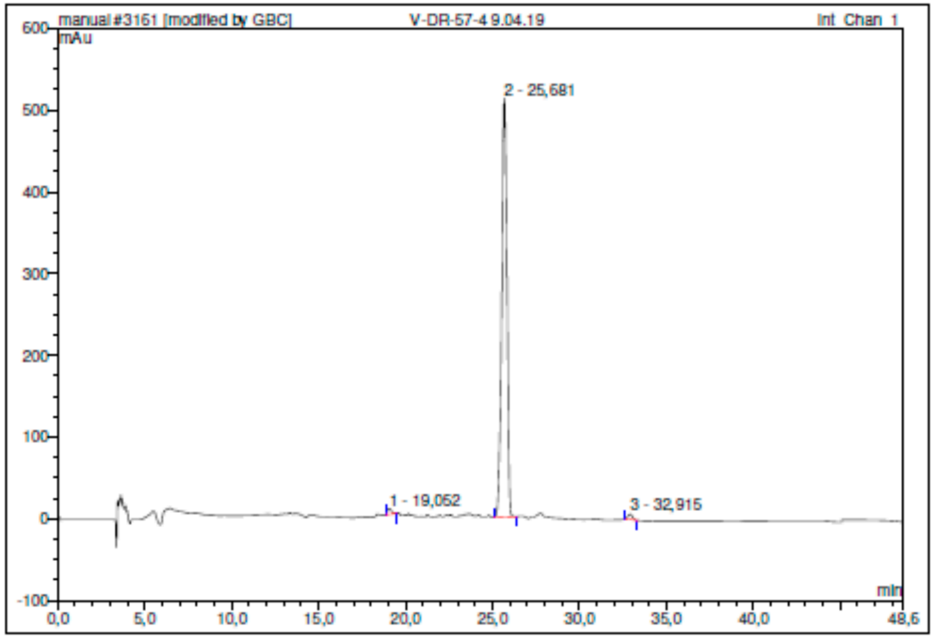

No.	Ret.Time min	Peak Name	Height mAu	Area mAu*min	Rel.Area %	Amount	Type
1	19,05	n.a.	6,984	1,928	1,06	n.a.	BMB*
2	25,68	n.a.	512,535	178,859	97,97	n.a.	BMB
3	32,92	n.a.	6,452	1,779	0,97	n.a.	BMB*
Total:			525,971	182,565	100,00	0,000	

Figure S11. HPLC analysis of compound **8**.

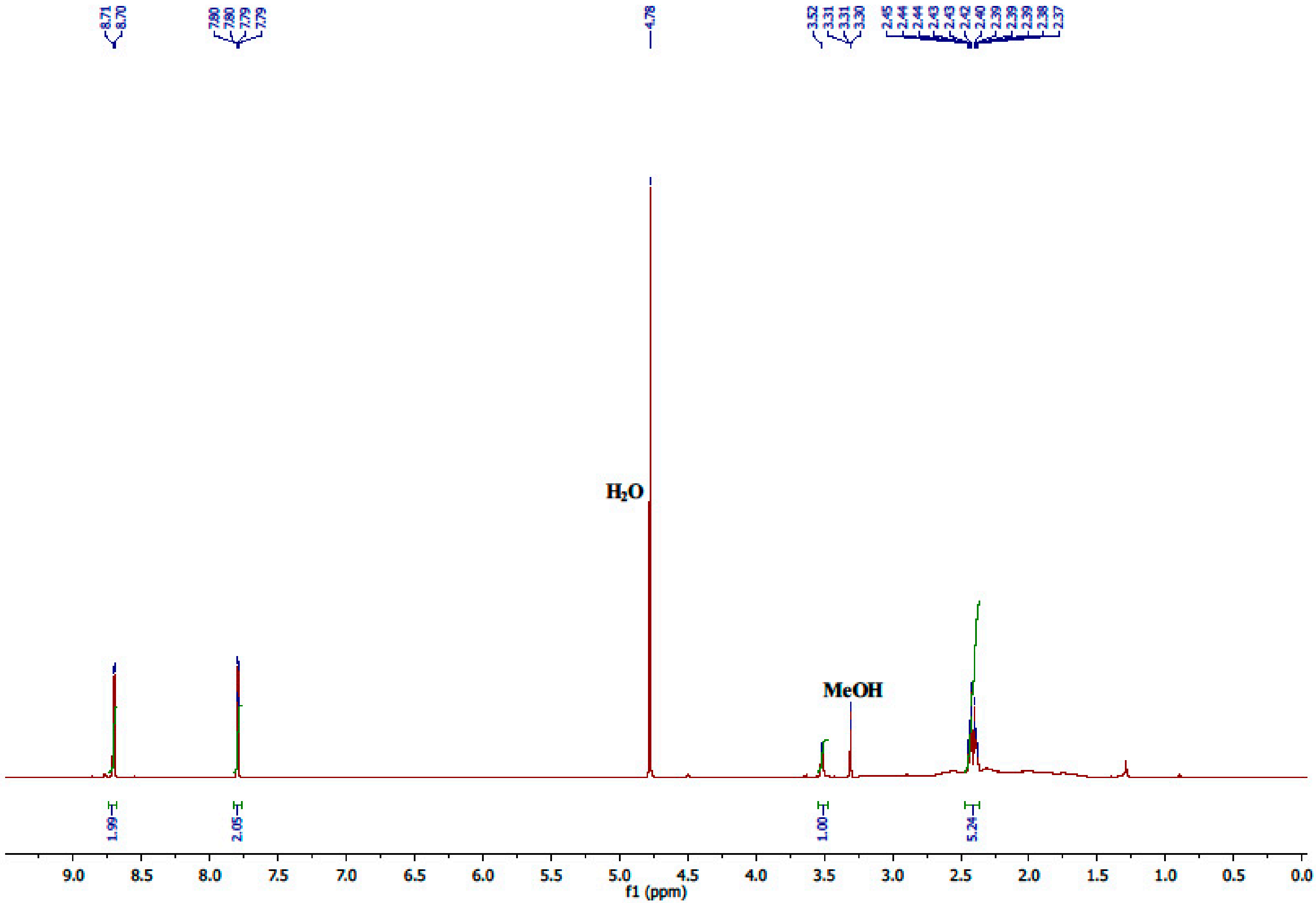

Figure S12. ^{1}H NMR spectrum of compound **9**.

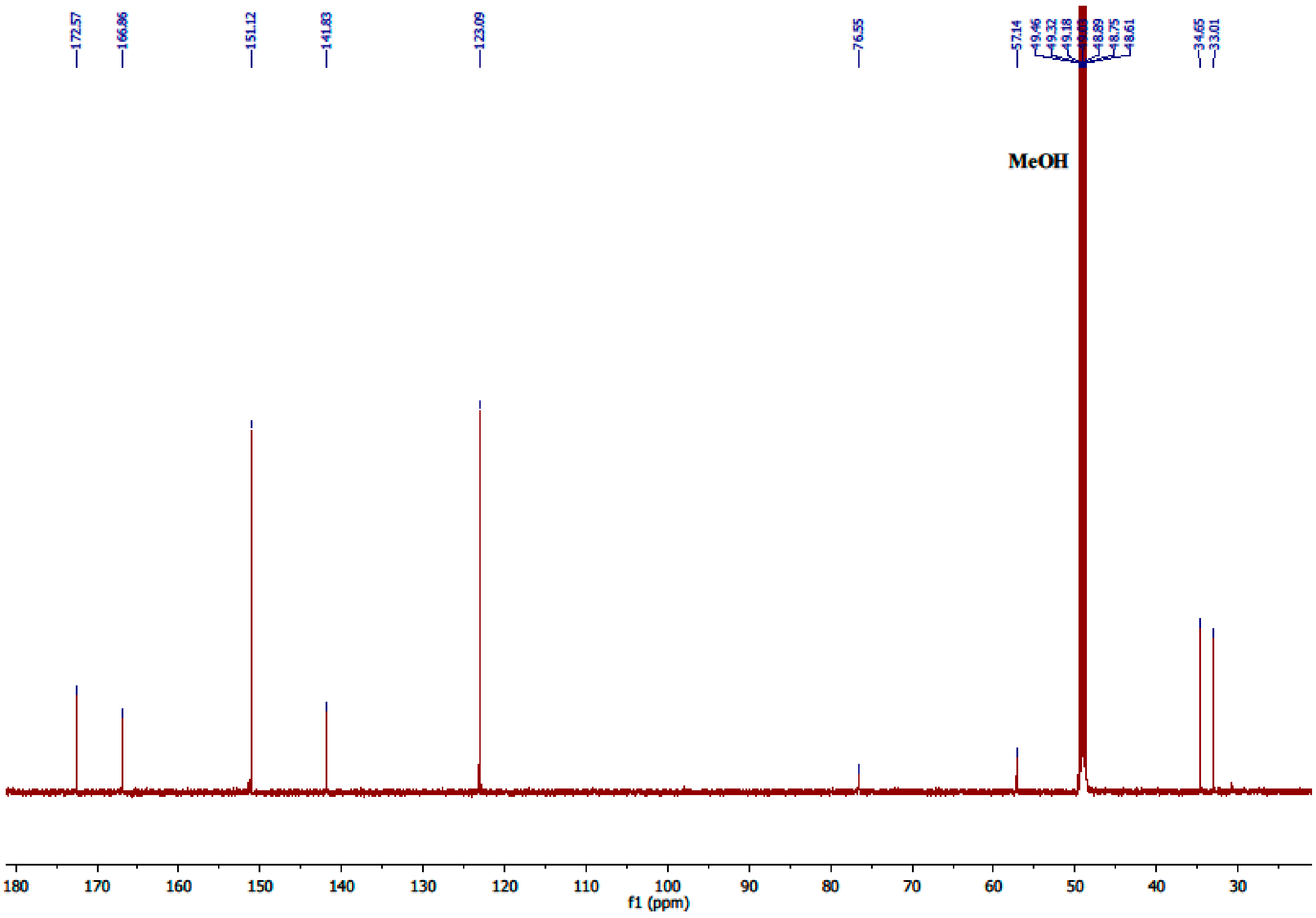

Figure S13. ${}^{13}C$ NMR spectrum of compound **9**.

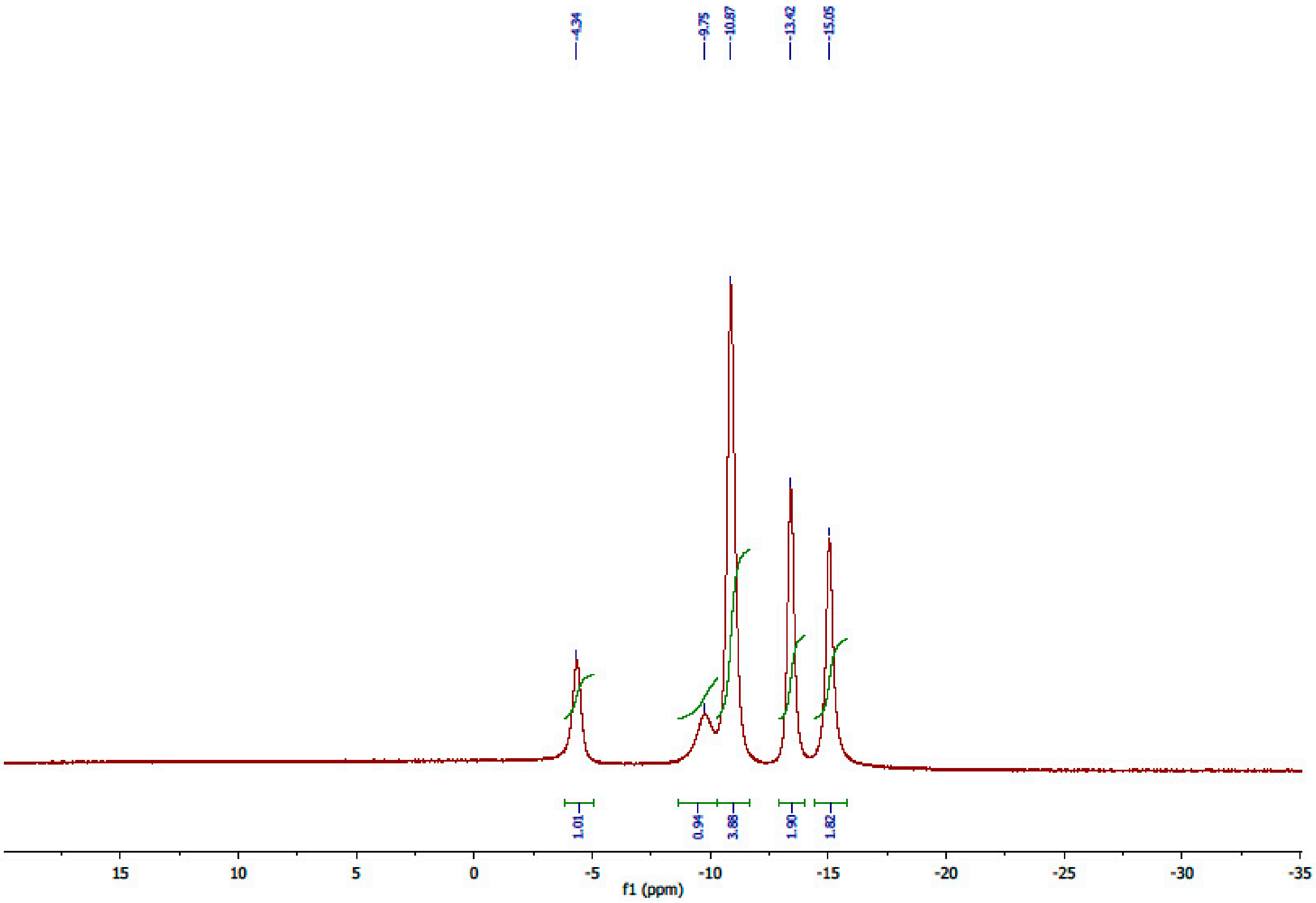

Figure S14. ^{11}B NMR {H BB} spectrum of compound **9**.

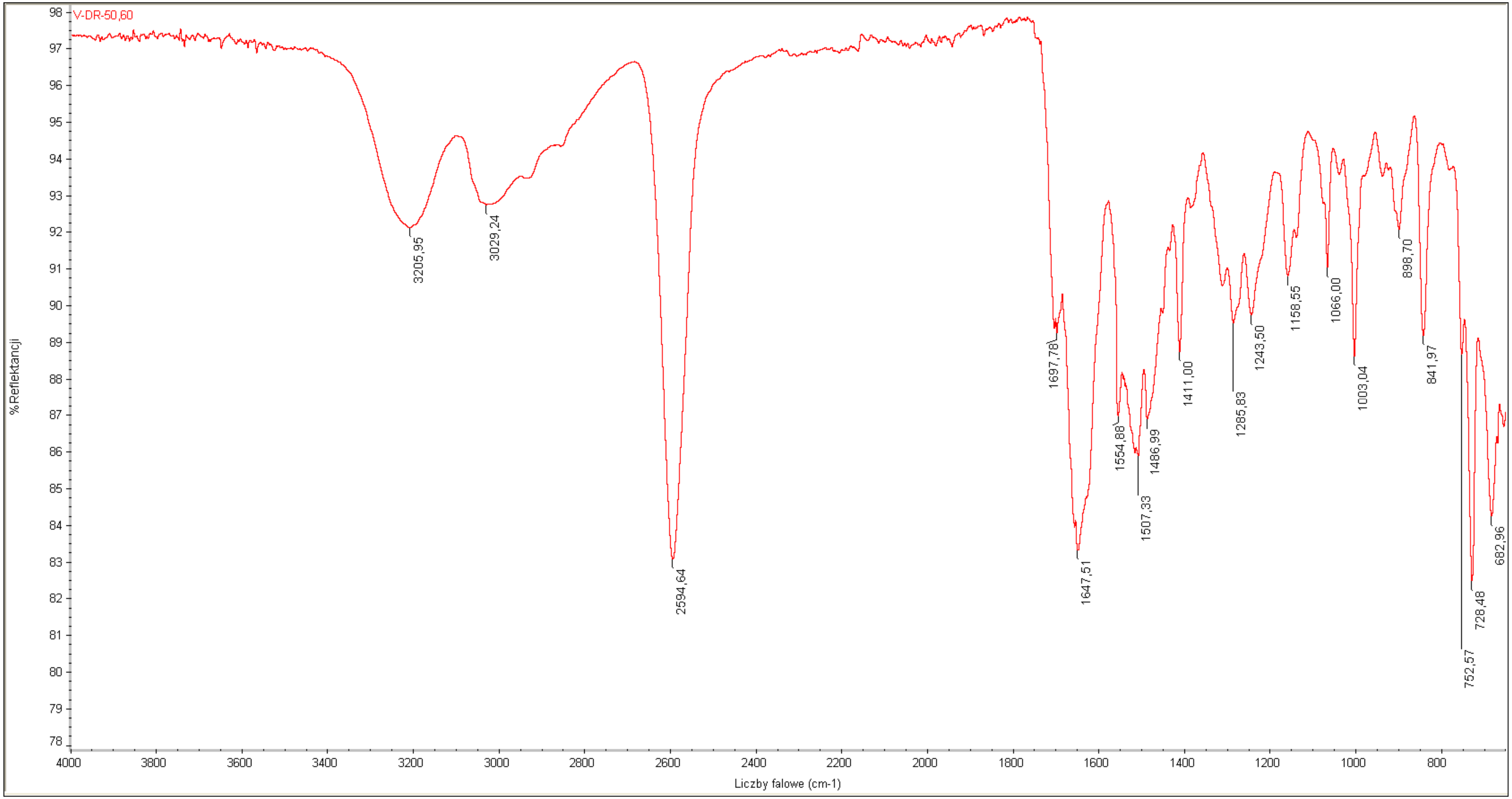

Figure S15. IR spectrum of compound **9**.

Spectrum Name: V-DR-50_60_typ_dod2
Start Ion: 100
End Ion: 500
Source: APCI + 10.0µA 400C
Capillary: 150V 300C Offset: 25V Span: 0V

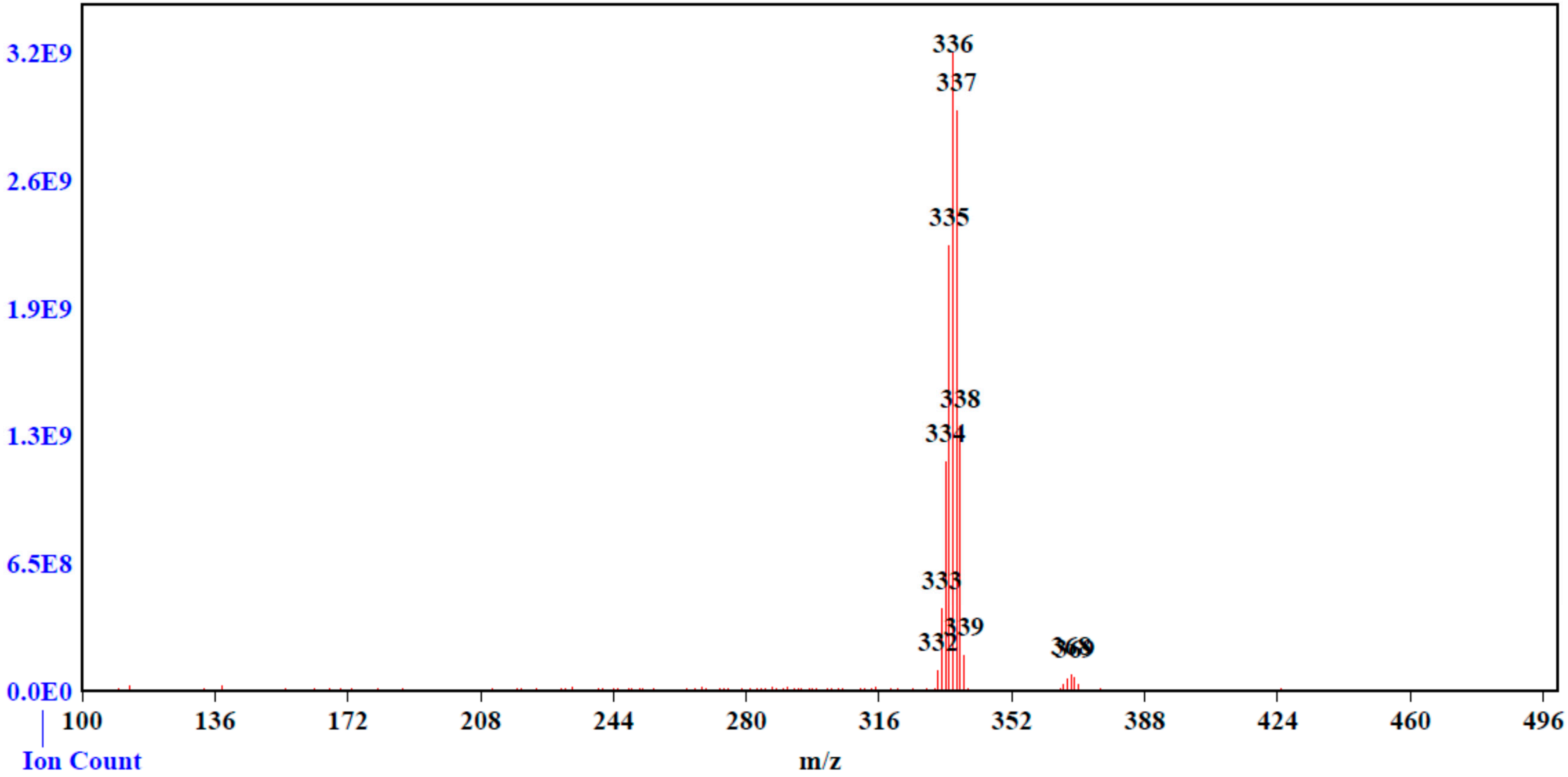

Figure S16. APCI-MS spectrum of compound **9**.

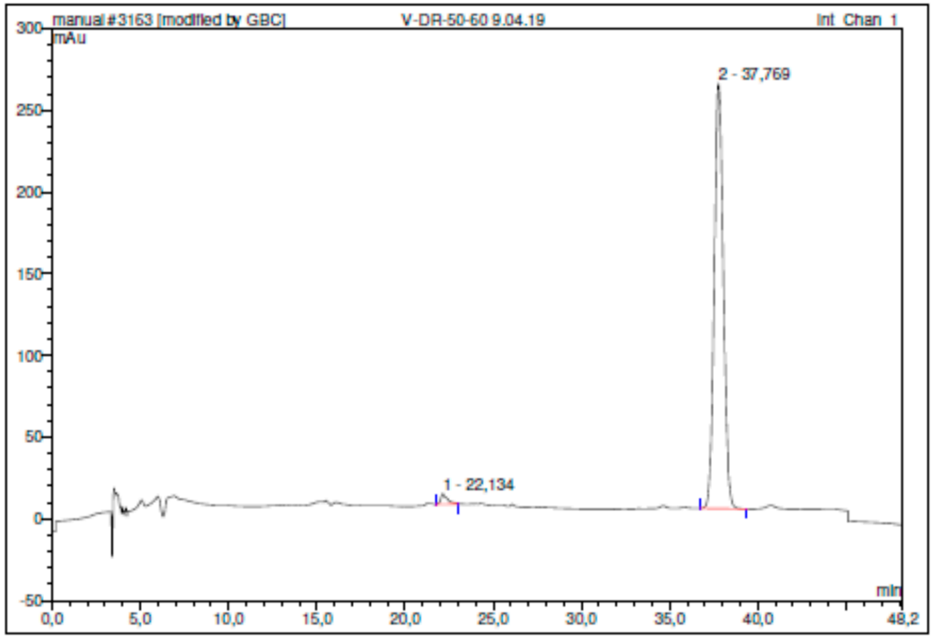

No.	Ret.Time min	Peak Name	Height mAu	Area mAu*min	Rel.Area %	Amount	Type
1	22,13	n.a.	6,494	2,681	1,70	n.a.	BMB*
2	37,77	n.a.	260,022	155,444	98,30	n.a.	BMB
Total:			266,516	158,124	100,00	0,000	

Figure S17. HPLC analysis of compound **9**.

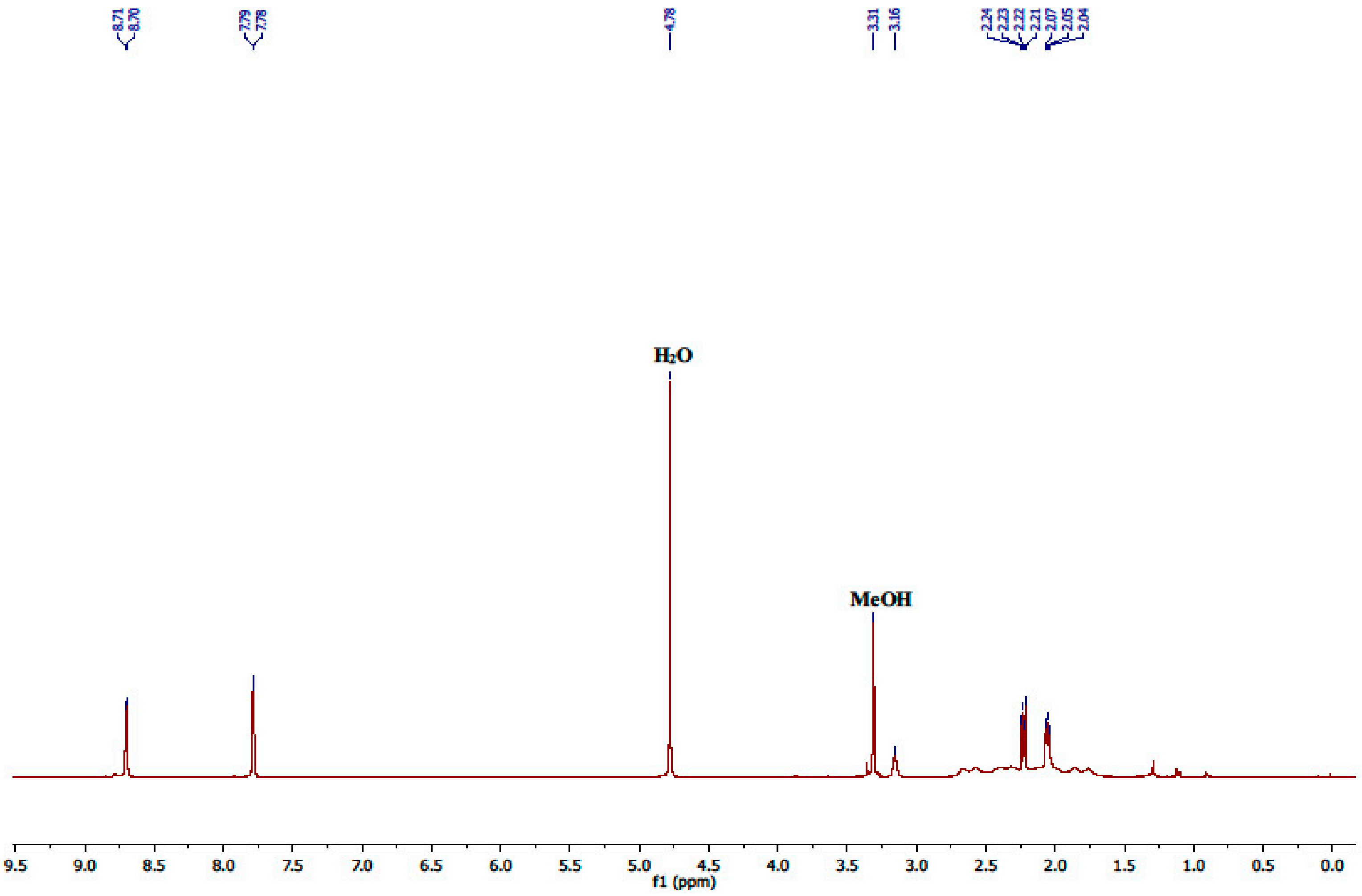

Figure S18. ^{1}H NMR spectrum of compound **10**.

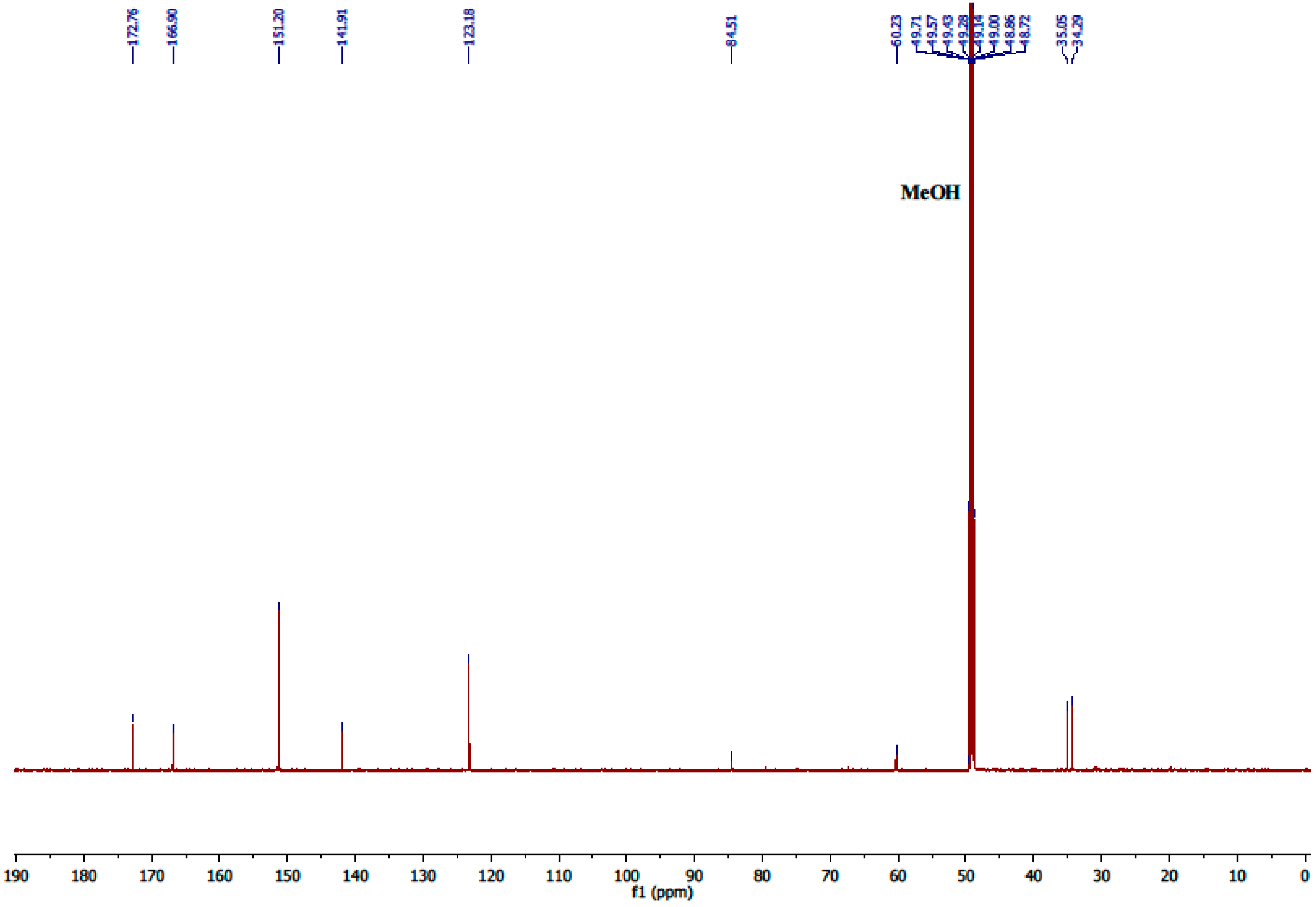

Figure S19. ^{13}C NMR spectrum of compound **10**.

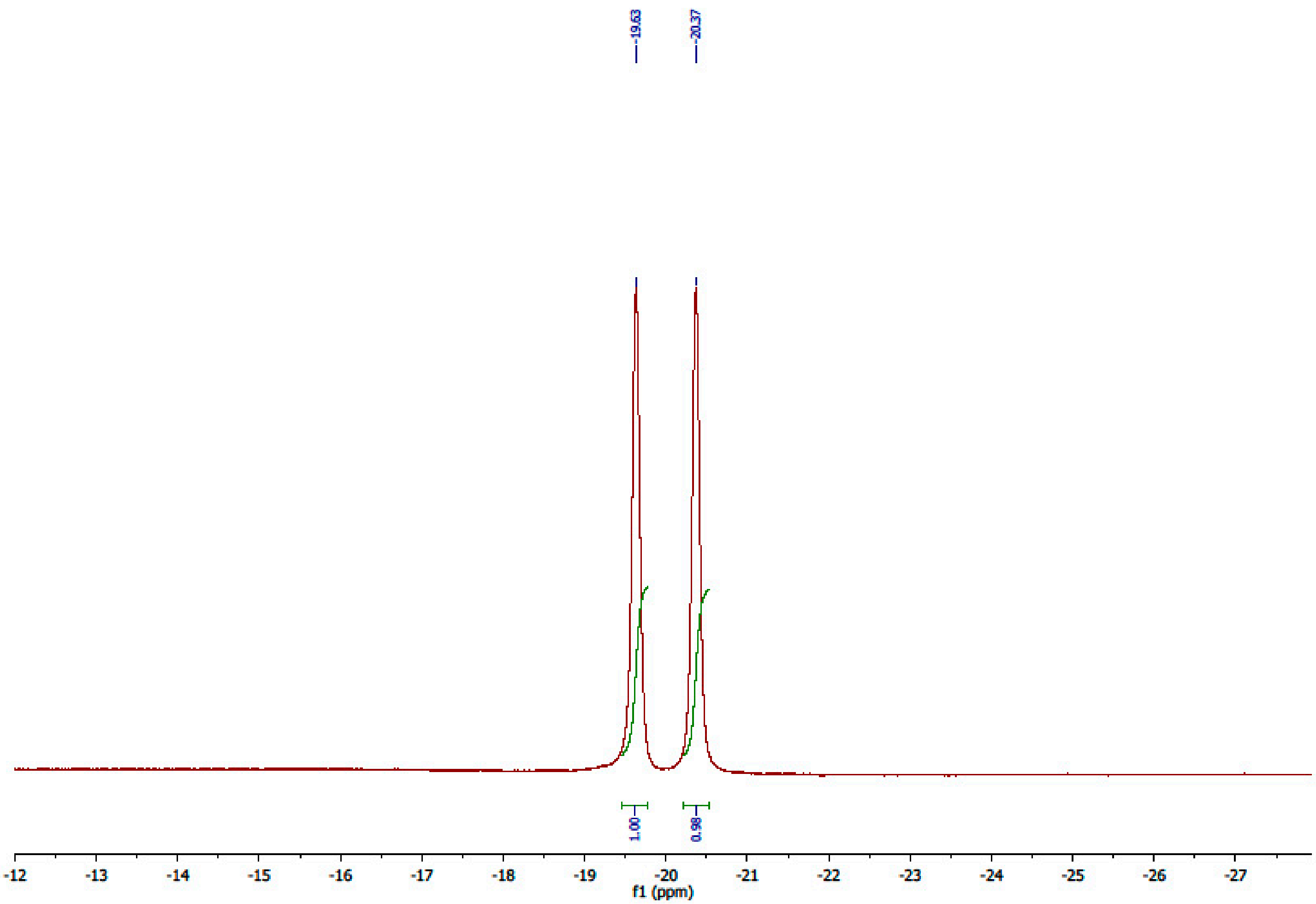

Figure S20. ^{11}B NMR {H BB} spectrum of compound **10**.

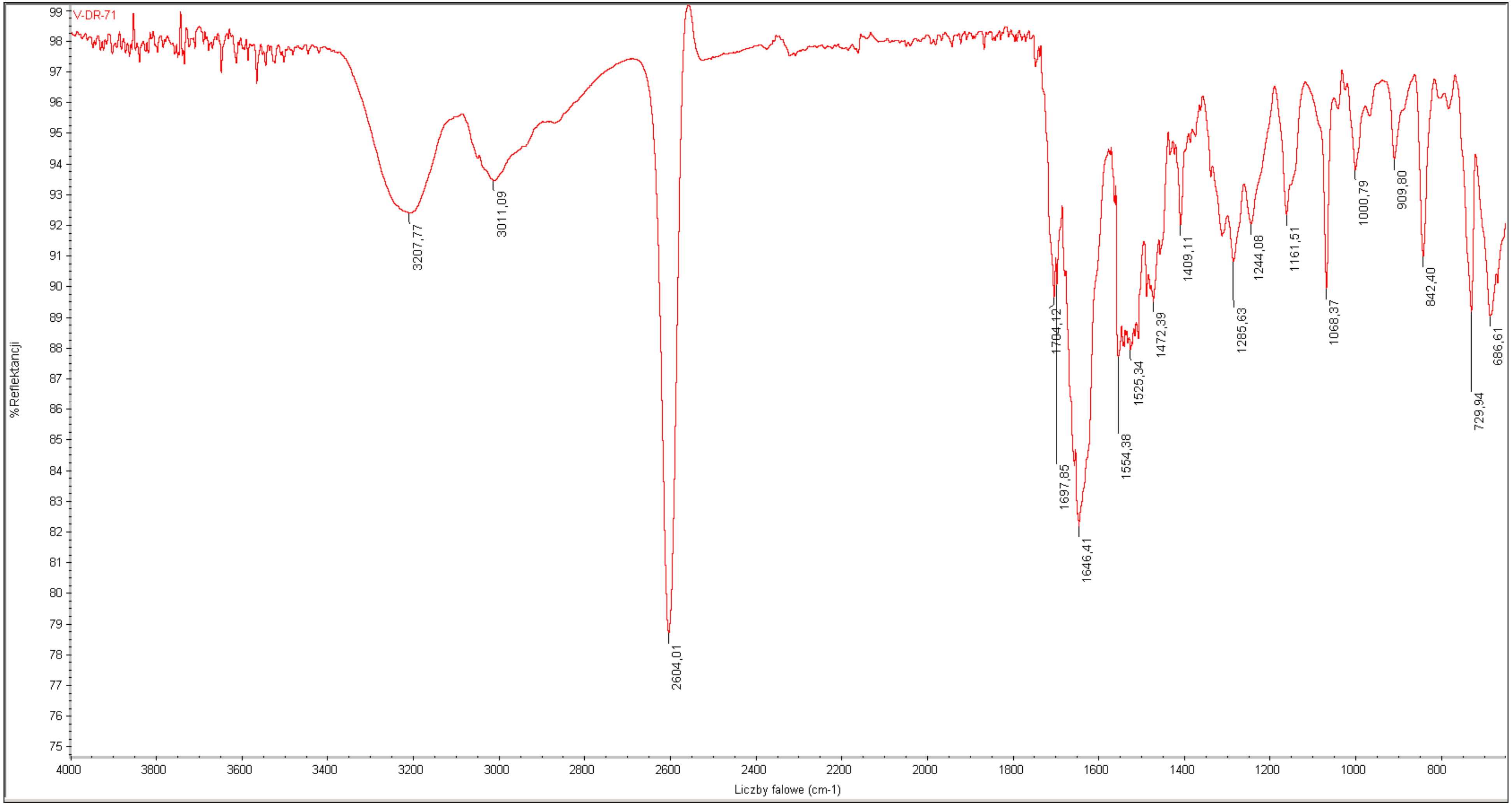

Figure S21. IR spectrum of compound **10**.

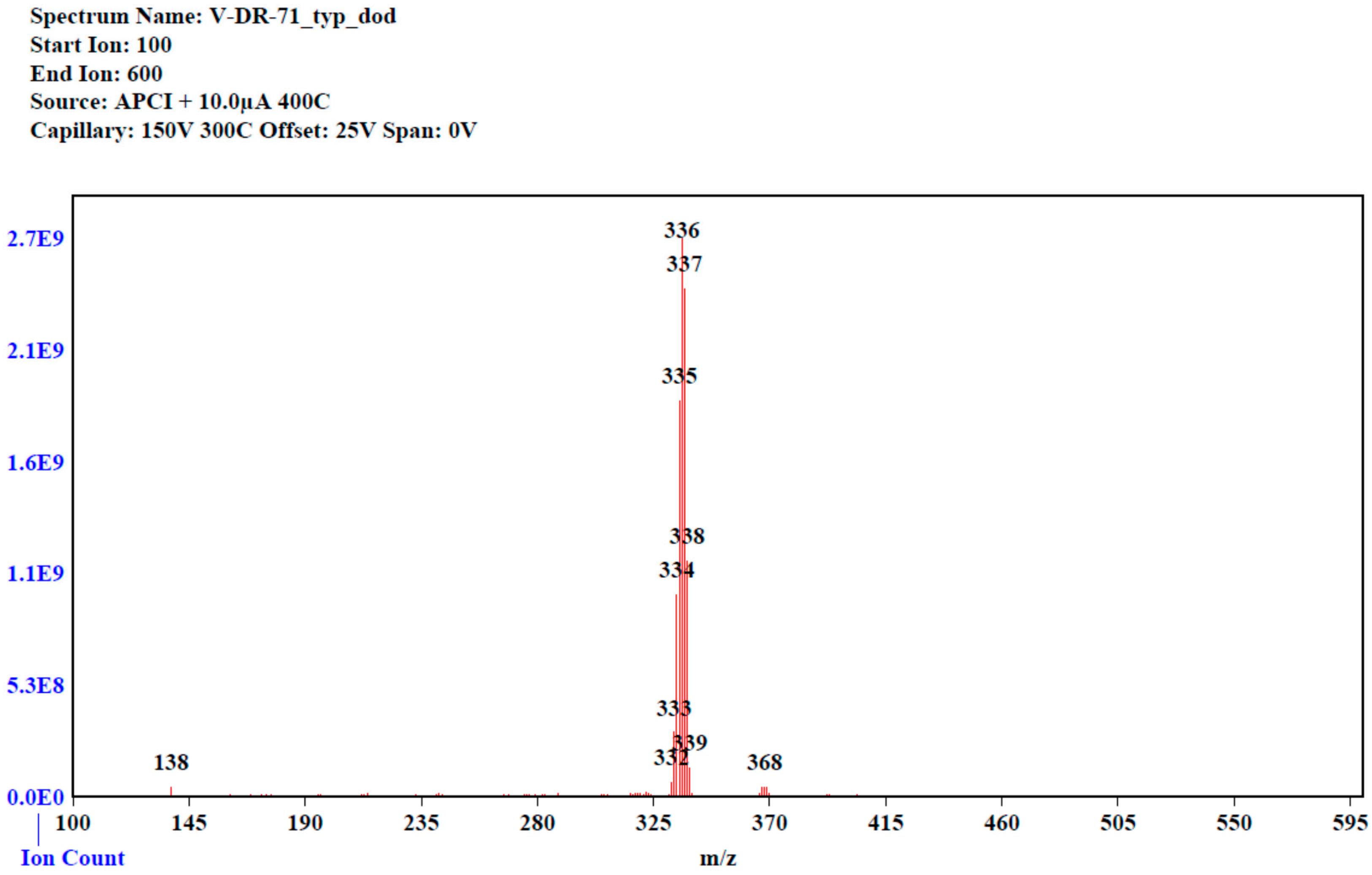

Figure S22. APCI-MS spectrum of compound **10**.

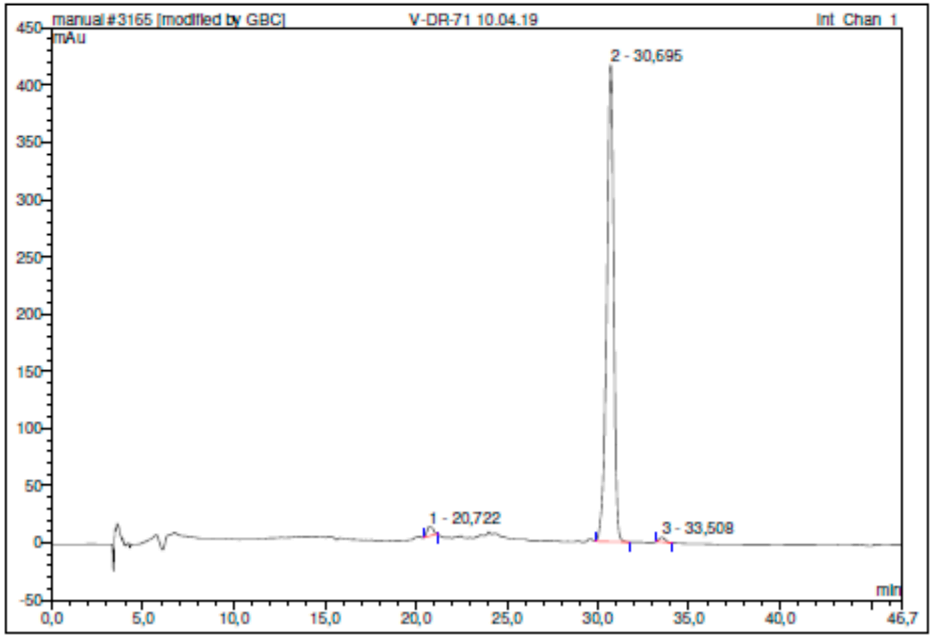

No.	Ret.Time min	Peak Name	Height mAu	Area mAu*min	Rel.Area %	Amount	Type
1	20,72	n.a.	8,134	3,028	1,60	n.a.	BMB*
2	30,69	n.a.	416,973	184,549	97,60	n.a.	BMB
3	33,51	n.a.	4,134	1,513	0,80	n.a.	BMB*
Total:			429,242	189,090	100,00	0,000	

Figure S23. HPLC analysis of compound **10**.

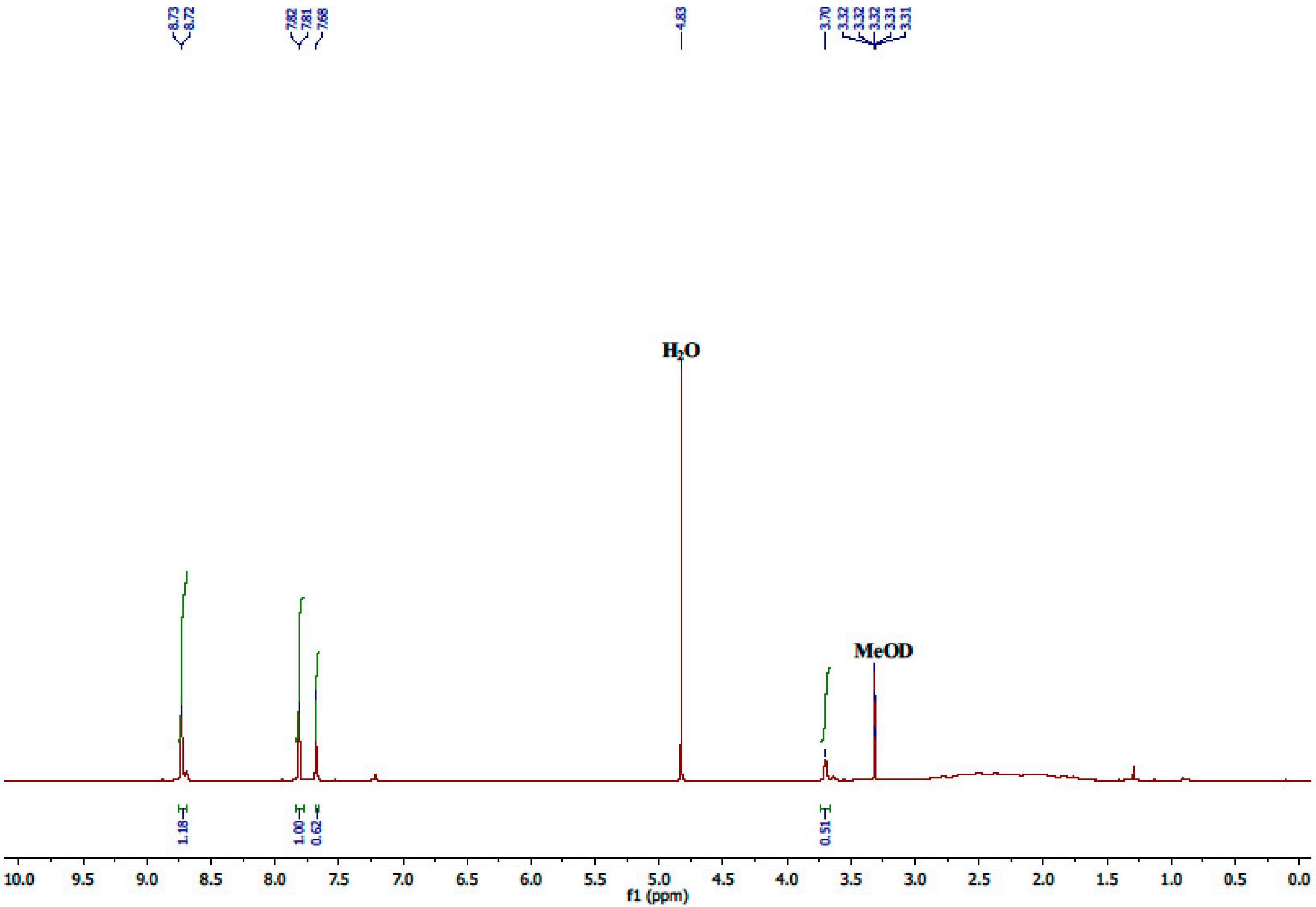

Figure S24. ^{1}H NMR spectrum of compound **14**.

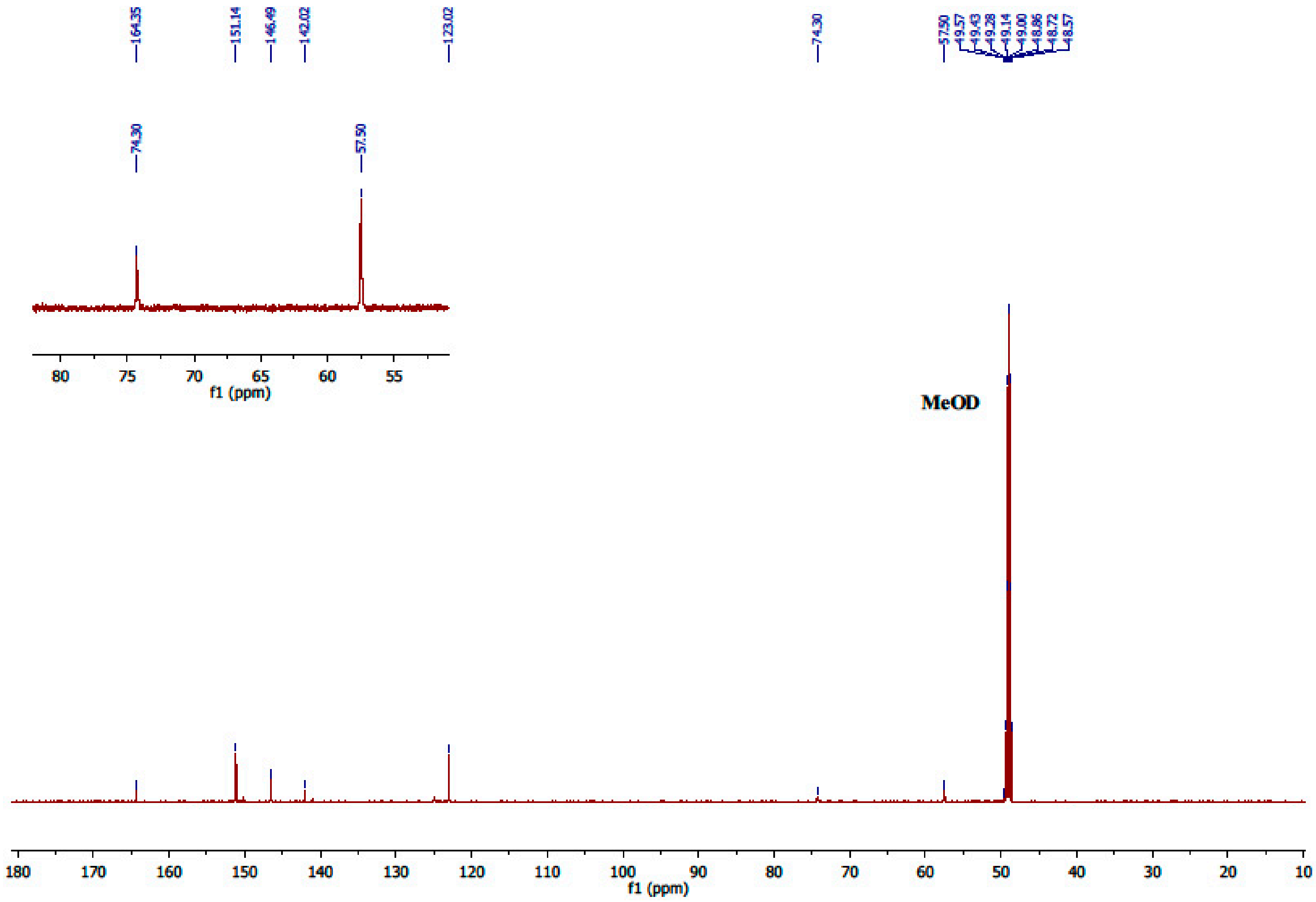

Figure S25. ^{13}C NMR spectrum of compound **14**.

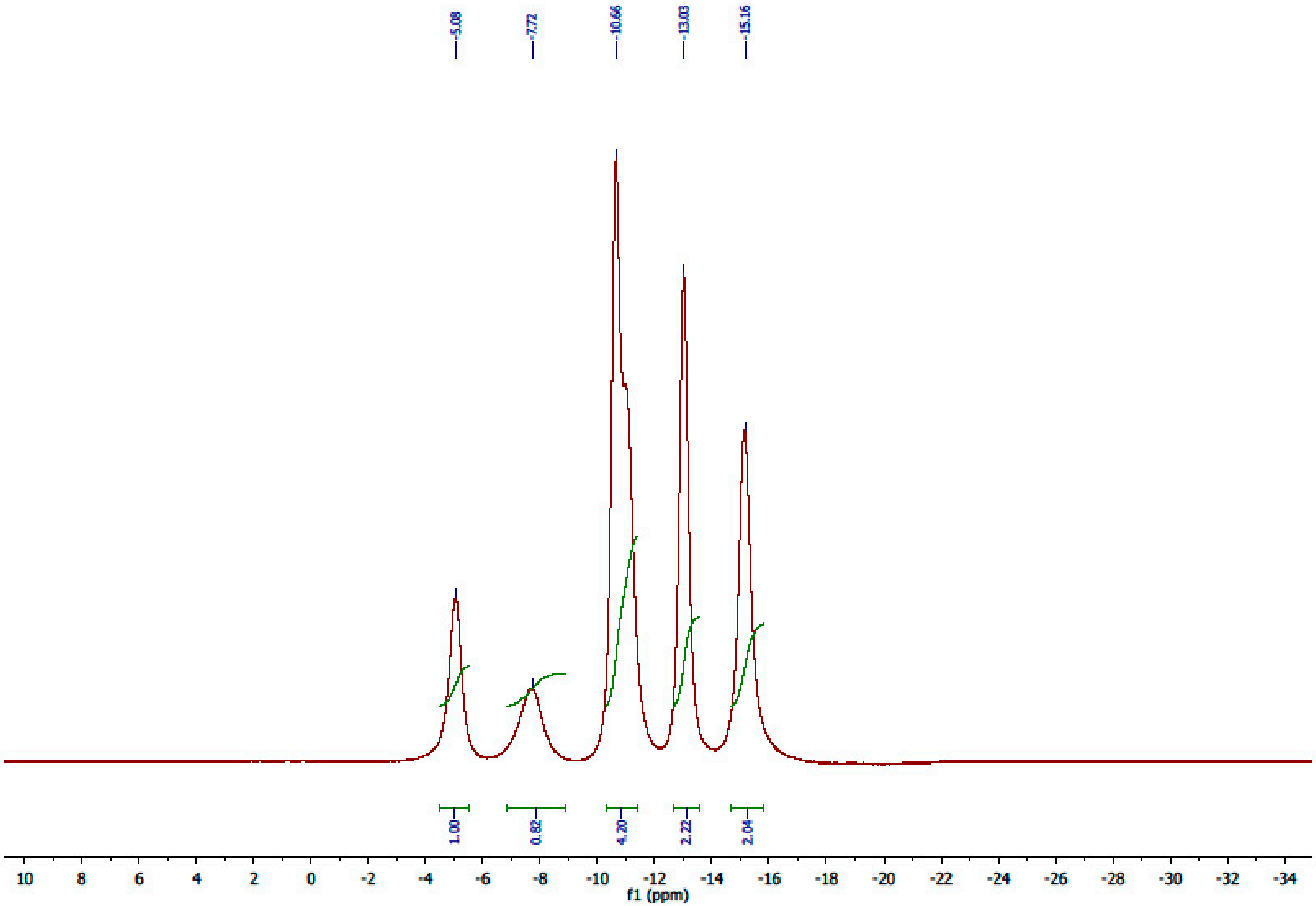

Figure S26. ^{11}B NMR {^{1}H BB} spectrum of compound **14**.

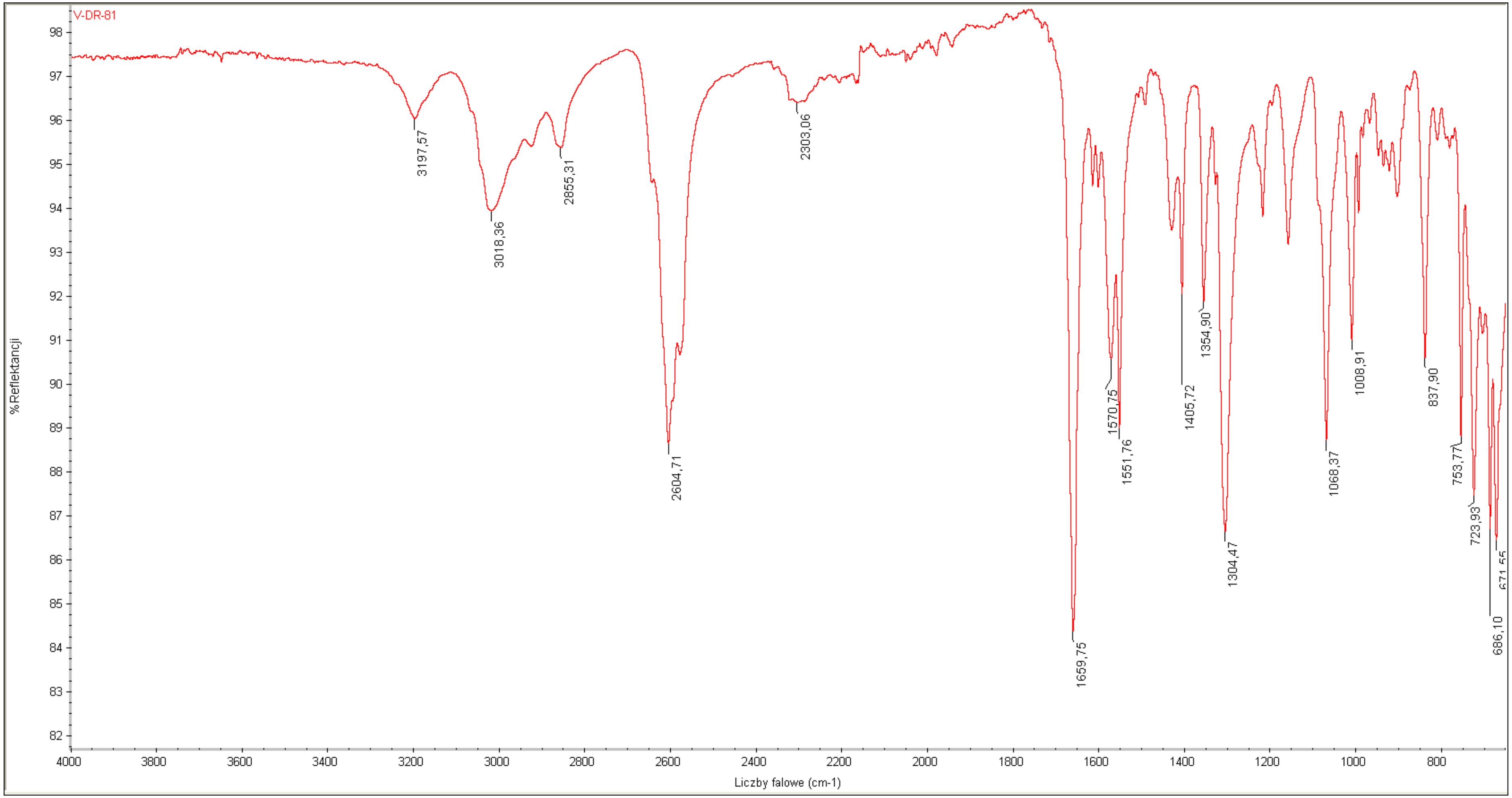

Figure S27. IR spectrum of compound **14**.

Spectrum Name: V-DR-81_rob_dod
Start Ion: 200
End Ion: 400
Source: APCI + 10.0µA 400C
Capillary: 180V 300C Offset: 30V Span: 20V

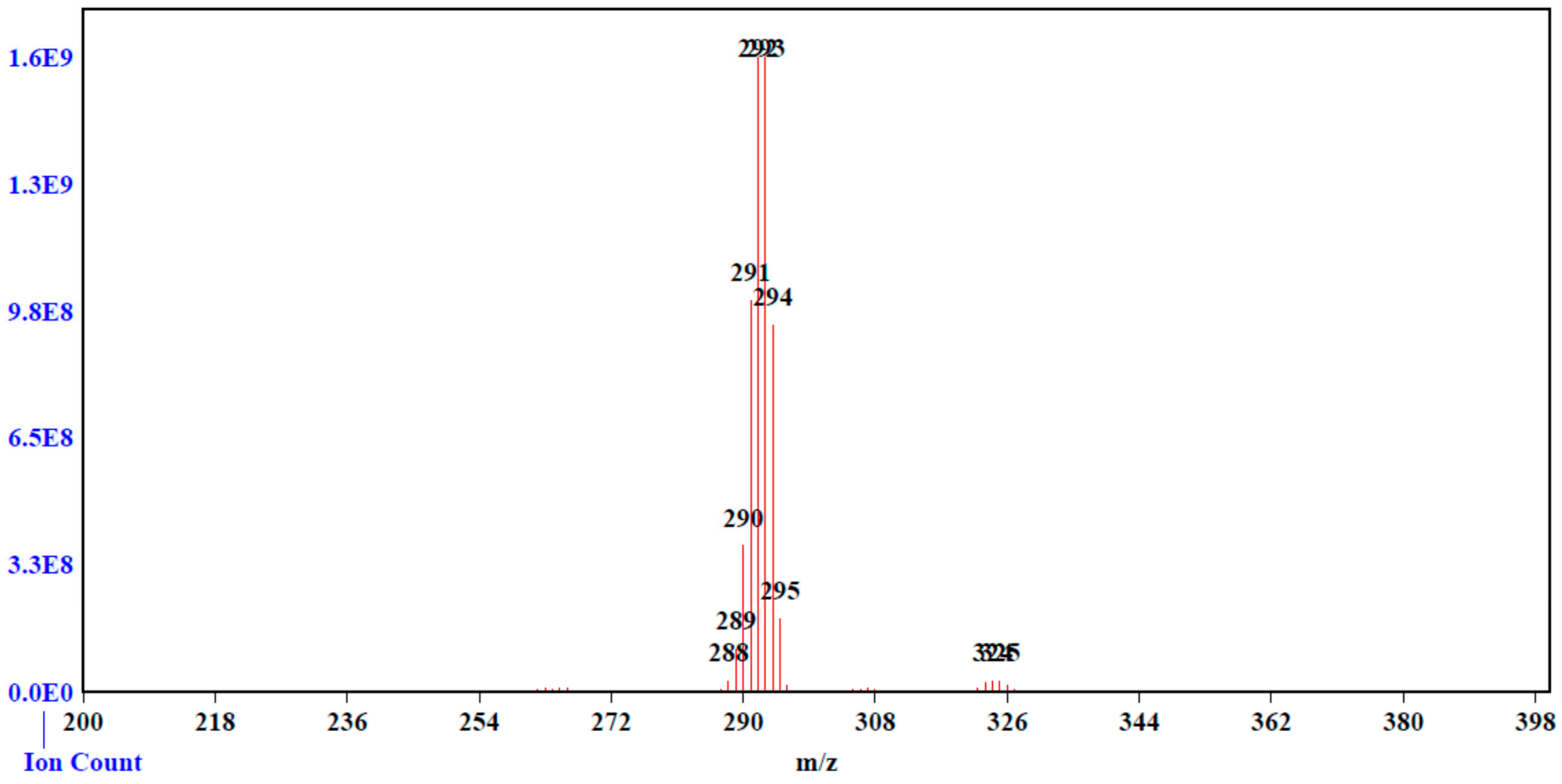

Figure S28. MS spectrum of compound **14**.

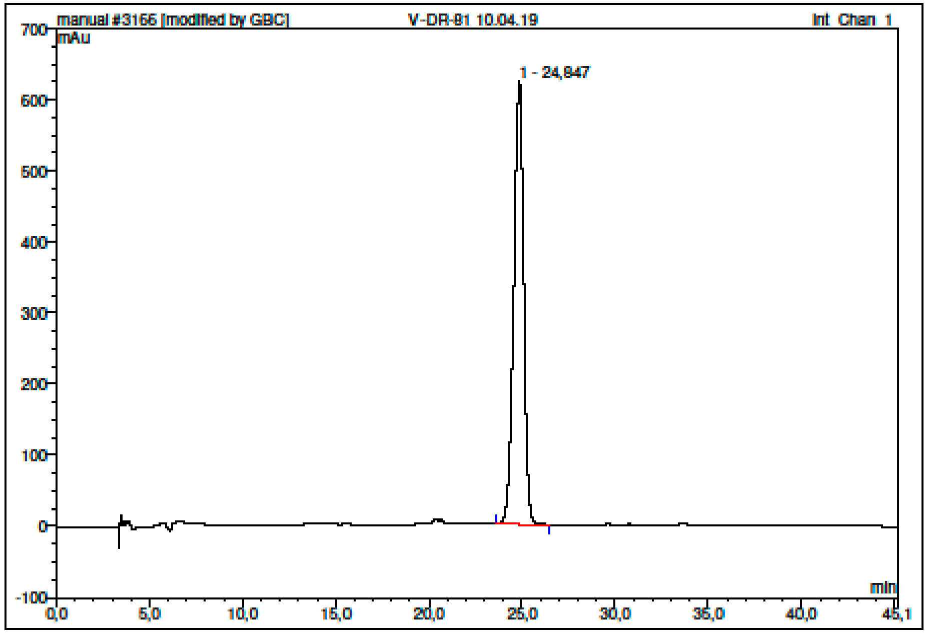

No.	Ret.Time min	Peak Name	Height mAu	Area mAu*min	Rel.Area %	Amount	Type
1	24,85	n.a.	622,459	391,932	100,00	n.a.	BMB
Total:			622,459	391,932	100,00	0,000	

Figure S29. HPLC analysis of compound **14**.

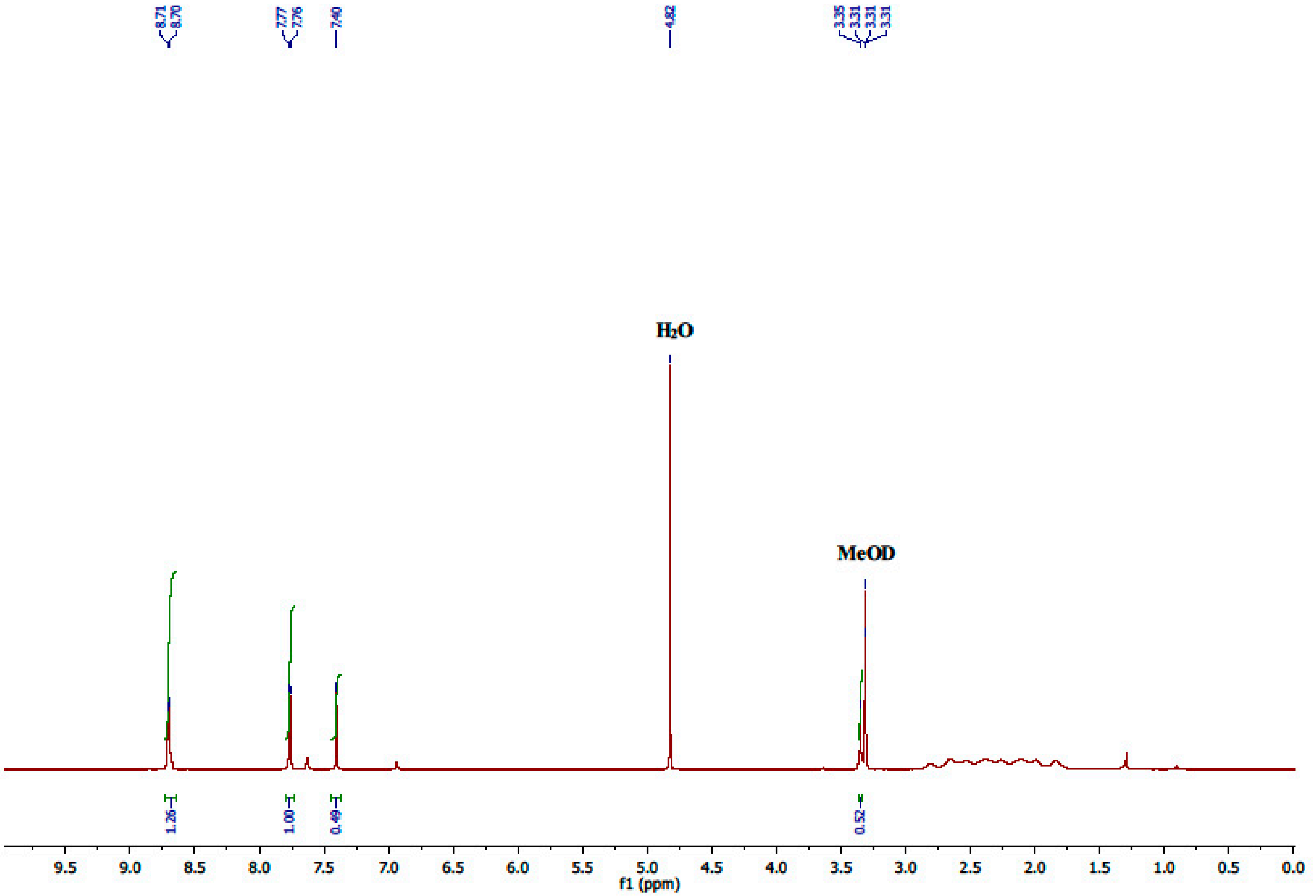

Figure S30. ^{1}H NMR spectrum of compound **15**.

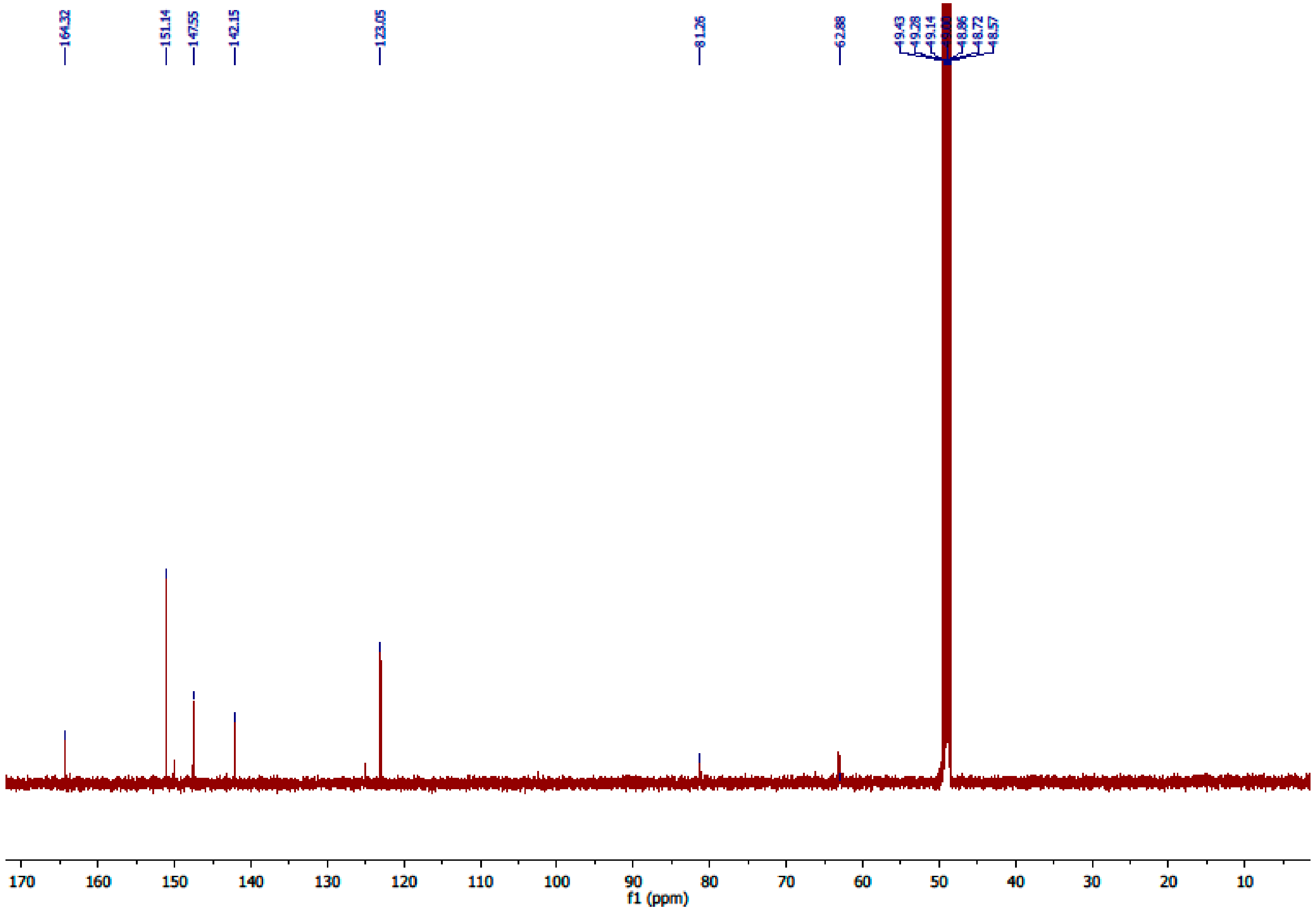

Figure S31. ^{13}C NMR spectrum of compound **15**.

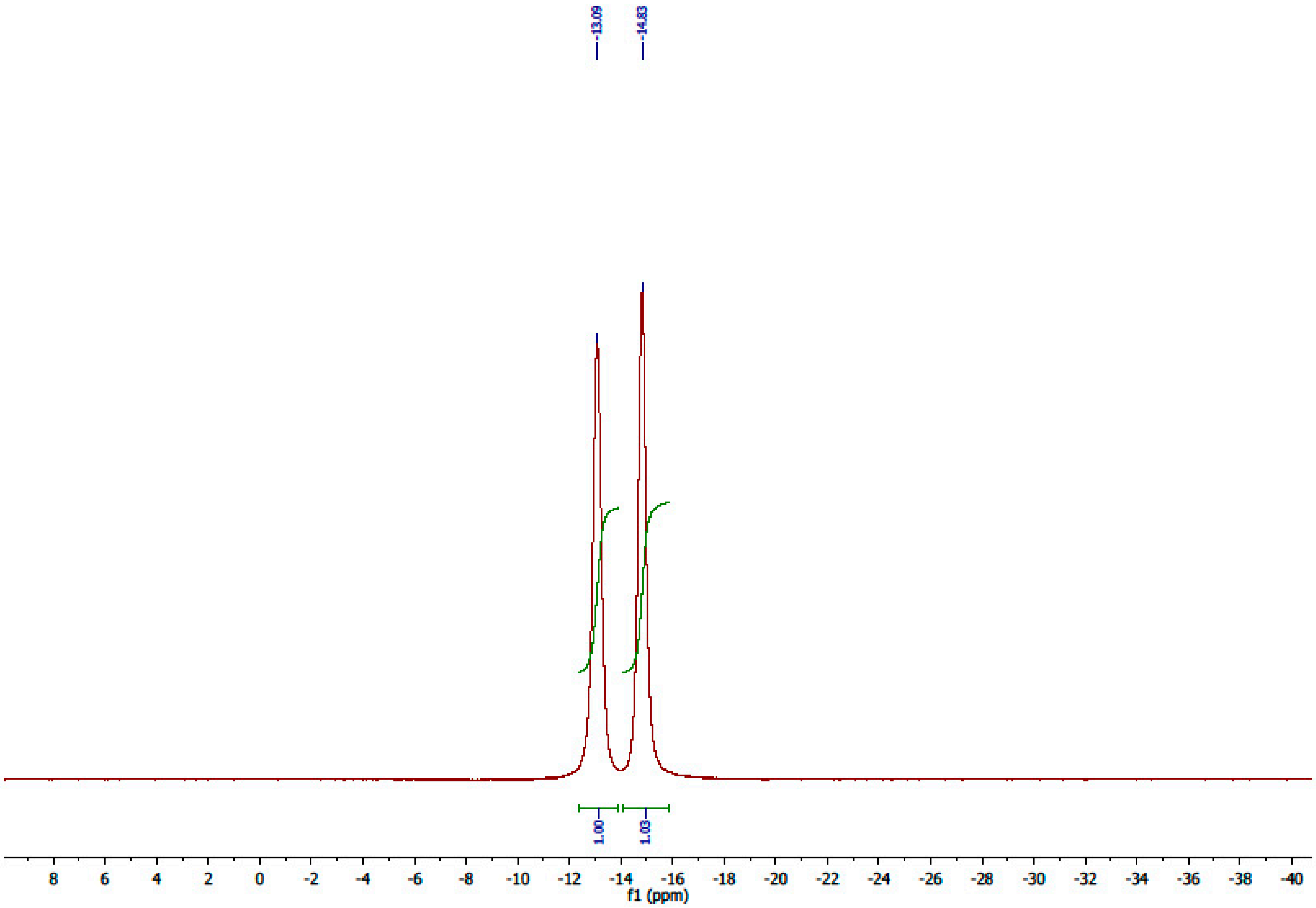

Figure S32. ^{11}B NMR {^{1}H BB} spectrum of compound **15**.

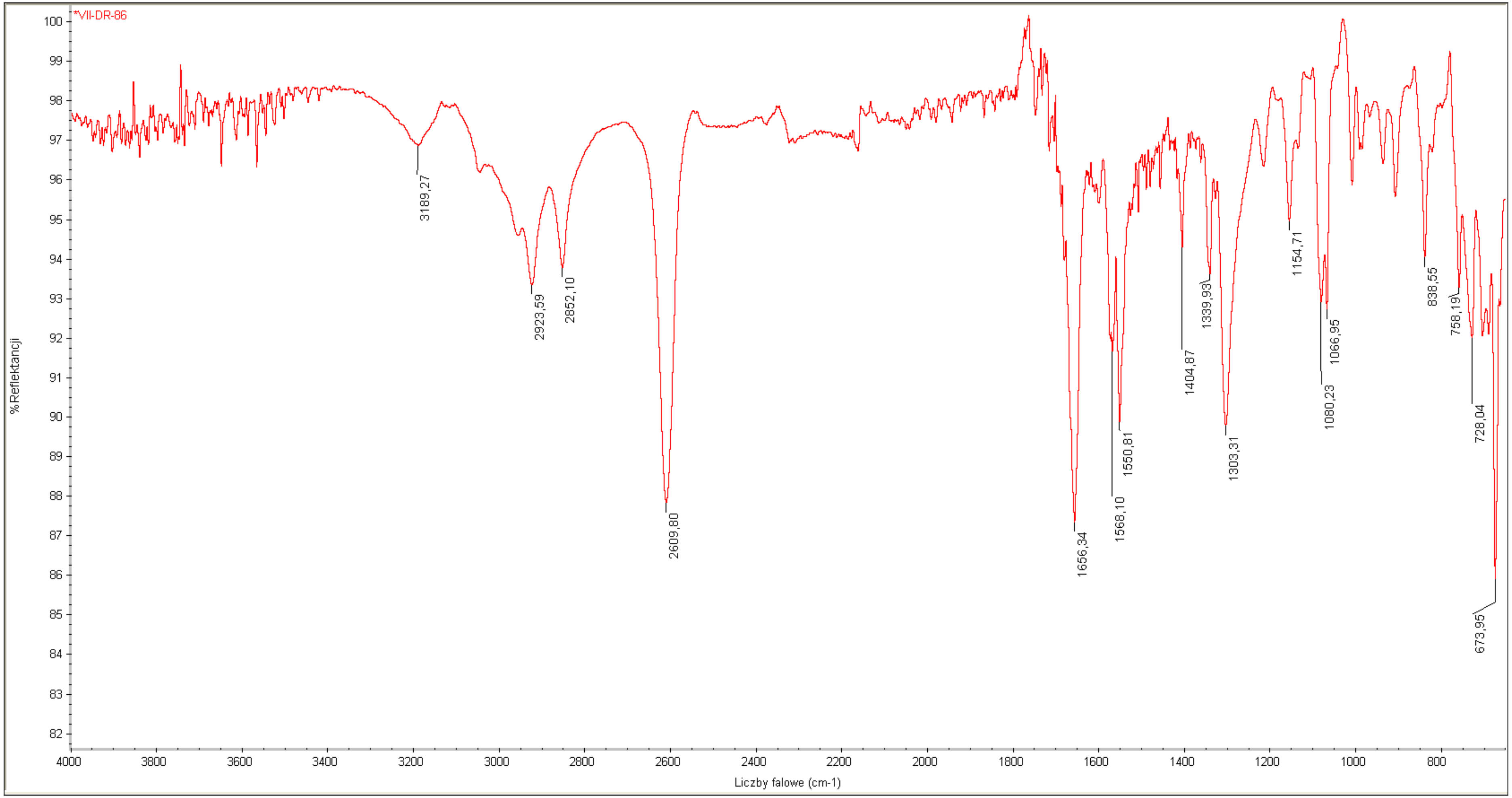

Figure S33. IR spectrum of compound **15**.

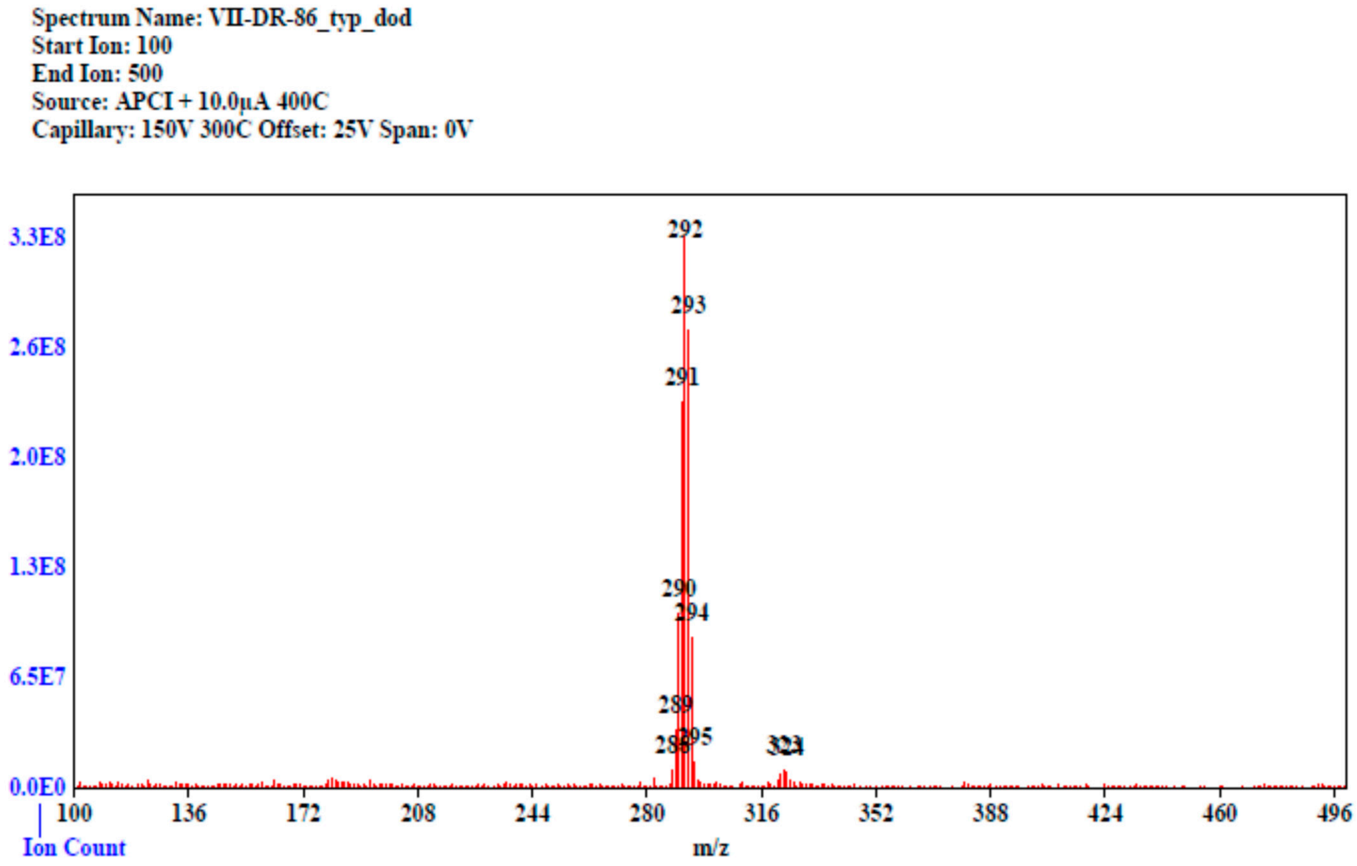

Figure S34. MS spectrum of compound 15.

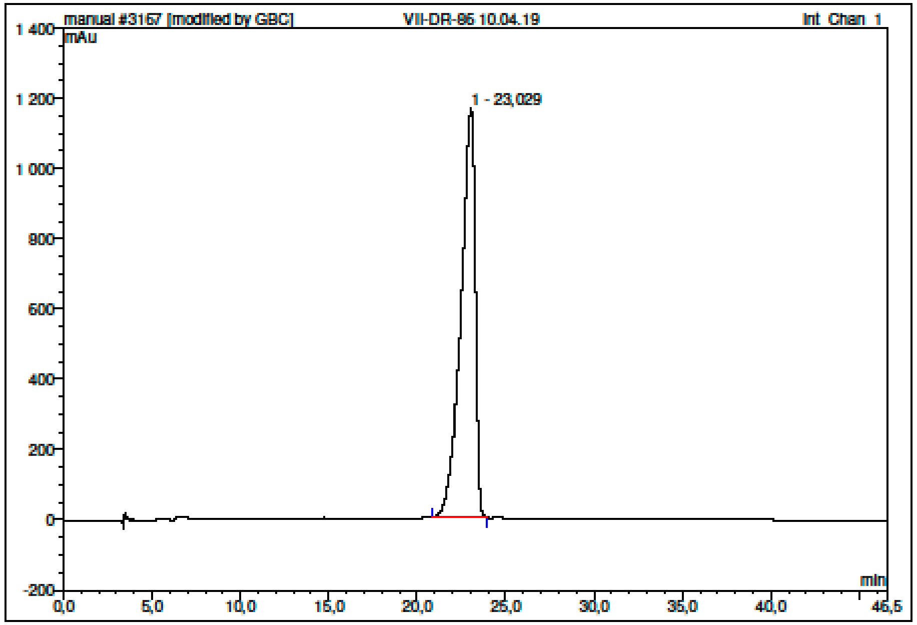

No.	Ret.Time min	Peak Name	Height mAu	Area mAu*min	Rel.Area %	Amount	Type
1	23,03	n.a.	1166,141	1101,737	100,00	n.a.	BMB
Total:			1166,141	1101,737	100,00	0,000	

Figure S35. HPLC analysis of compound **15**.

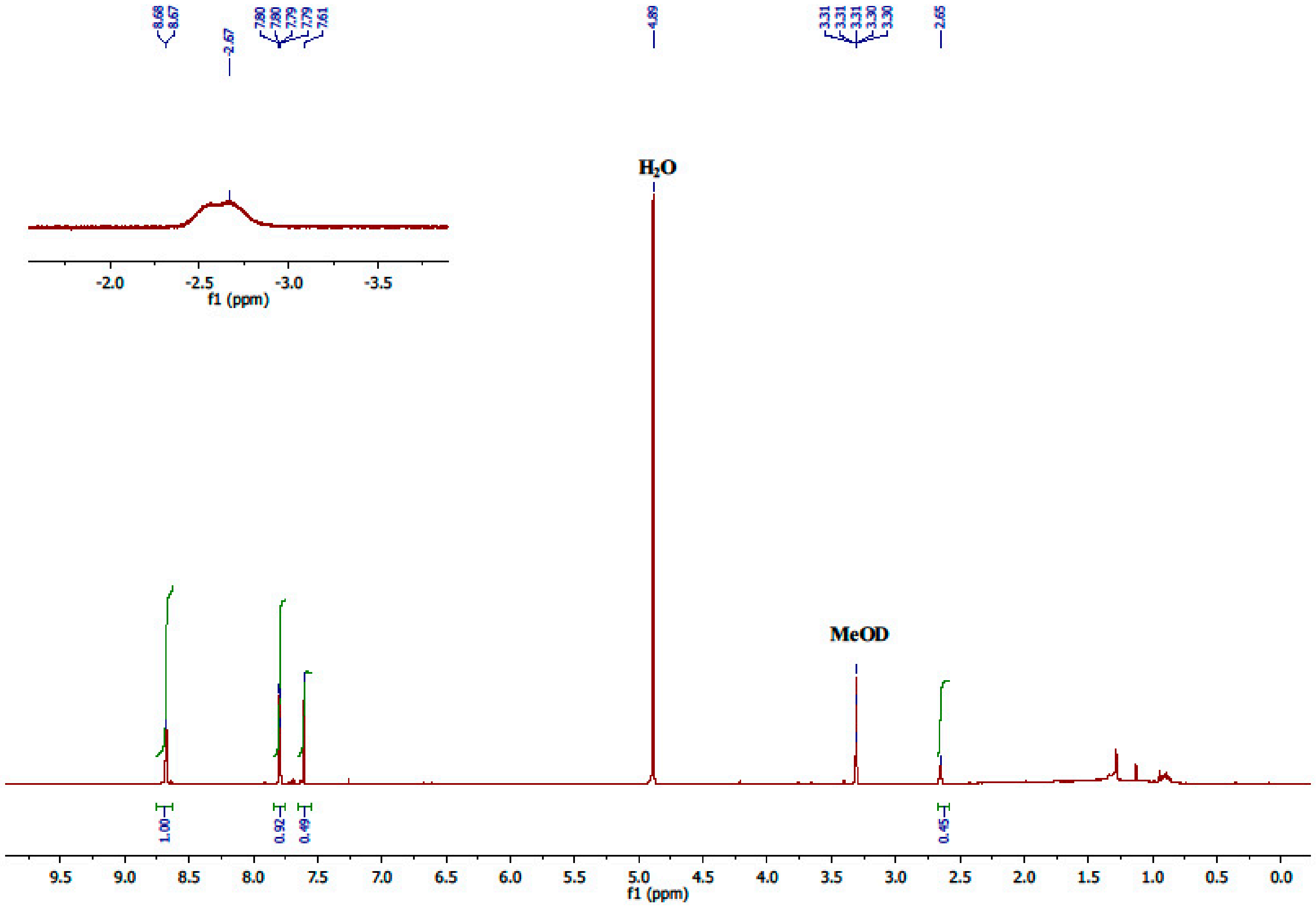

Figure S36. ^{1}H NMR spectrum of compound **16**.

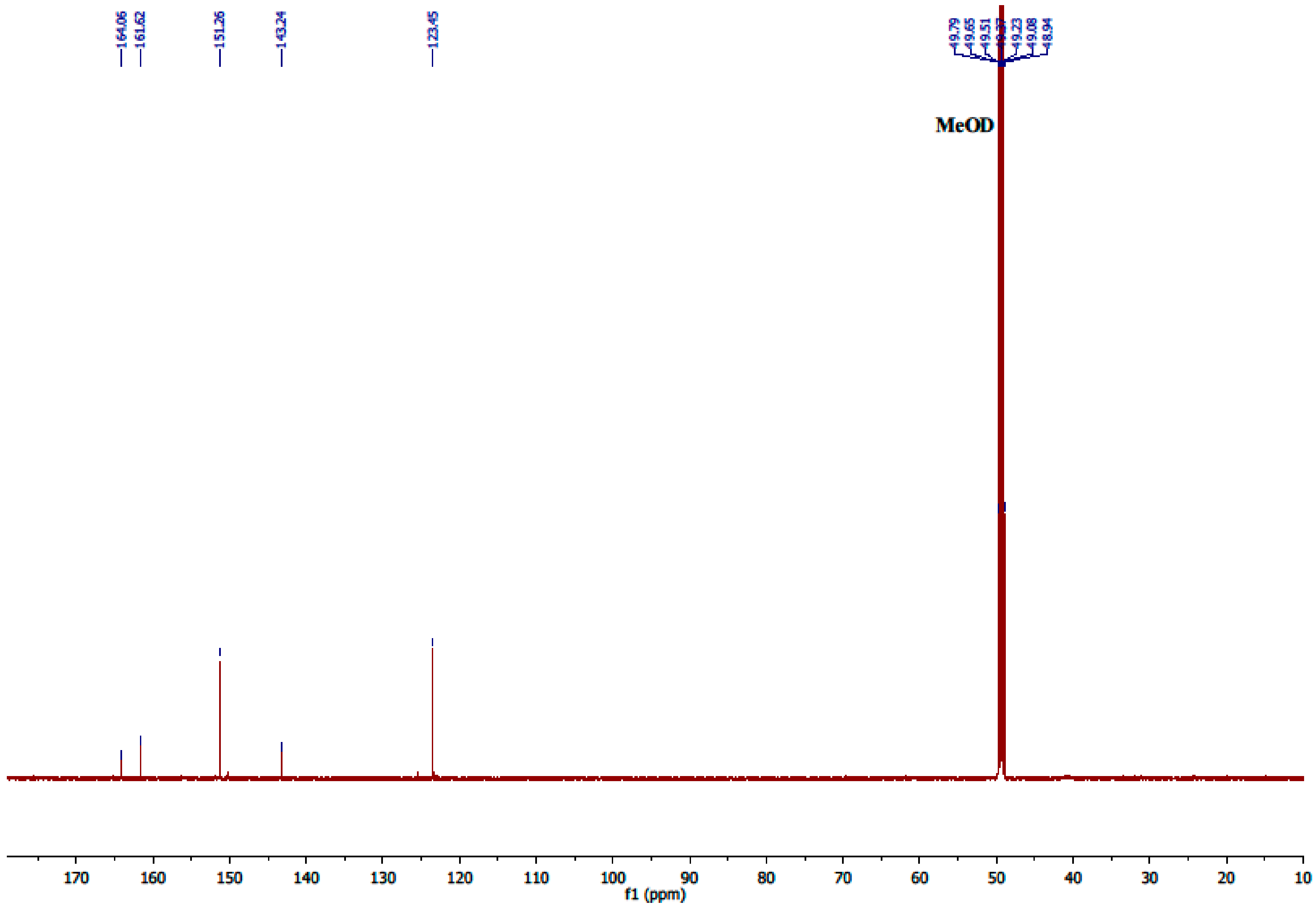

Figure S37. ^{13}C NMR spectrum of compound **16**.

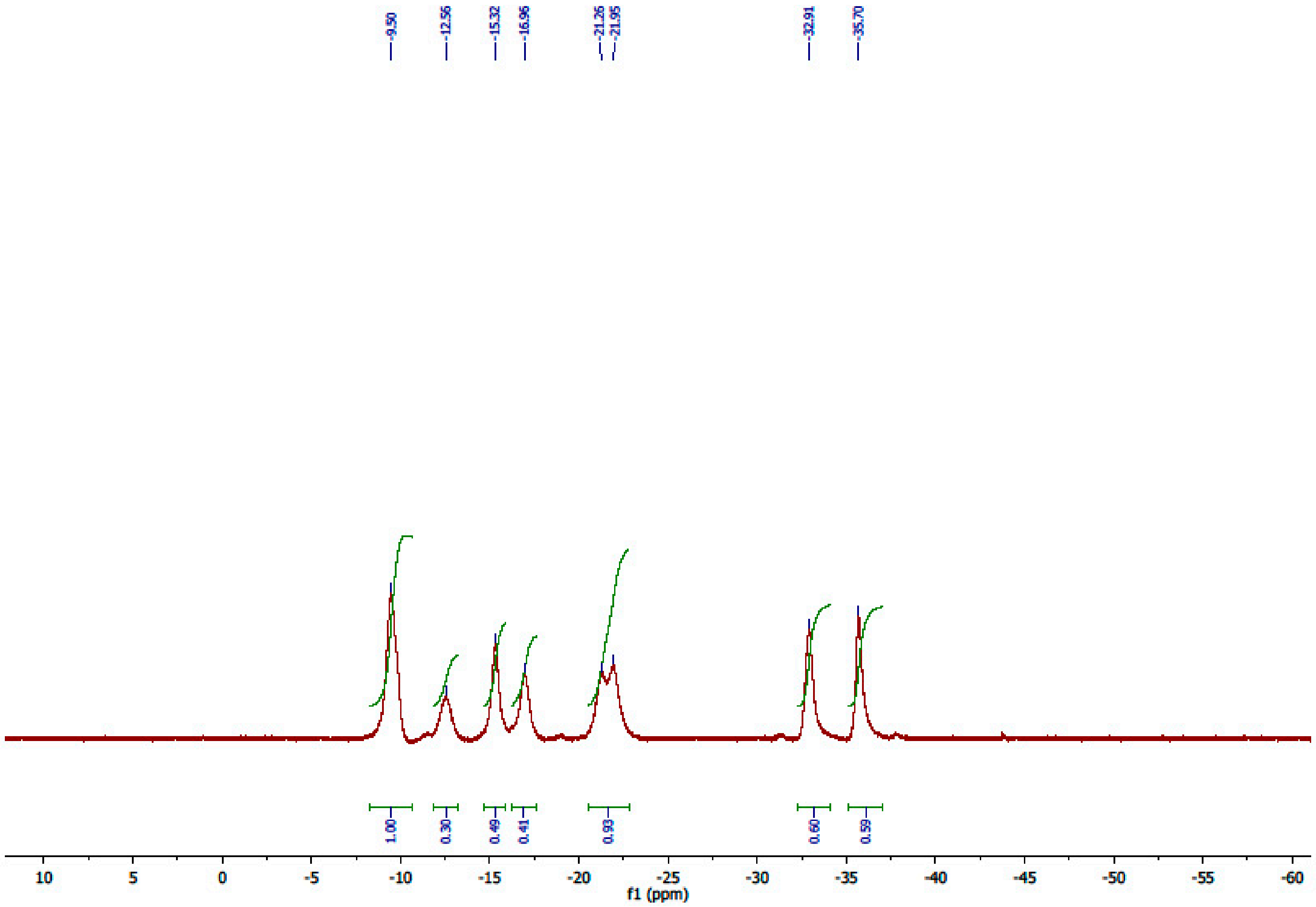

Figure S38. ^{11}B NMR {^{1}H BB} spectrum of compound **16**.

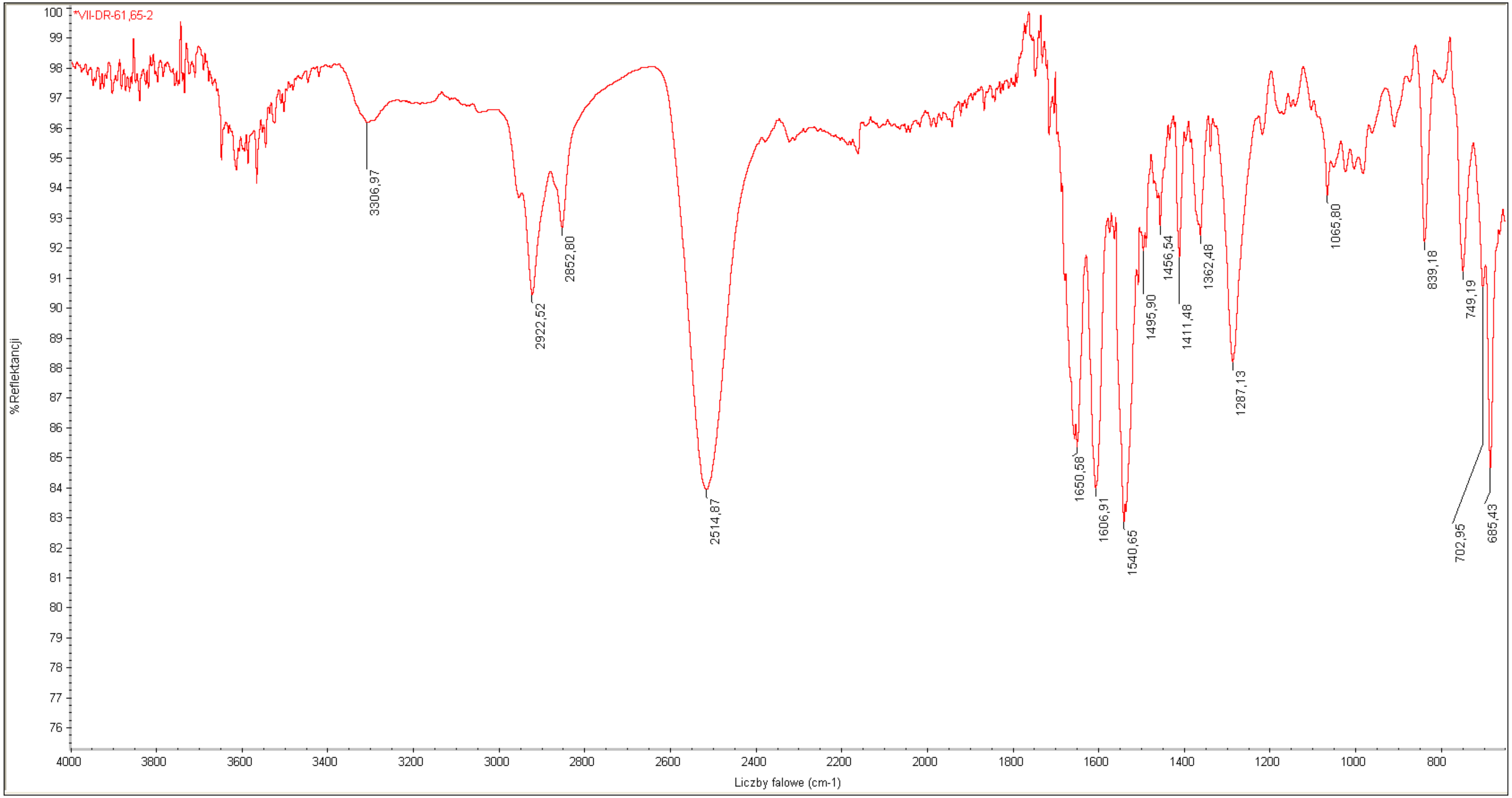

Figure S39. IR spectrum of compound **16**.

Spectrum Name: VII-DR-46_fr3_rob_min
Start Ion: 100
End Ion: 400
Source: ESI - 2.5kV 350C
Capillary: 180V 300C Offset: 30V Span: 20V

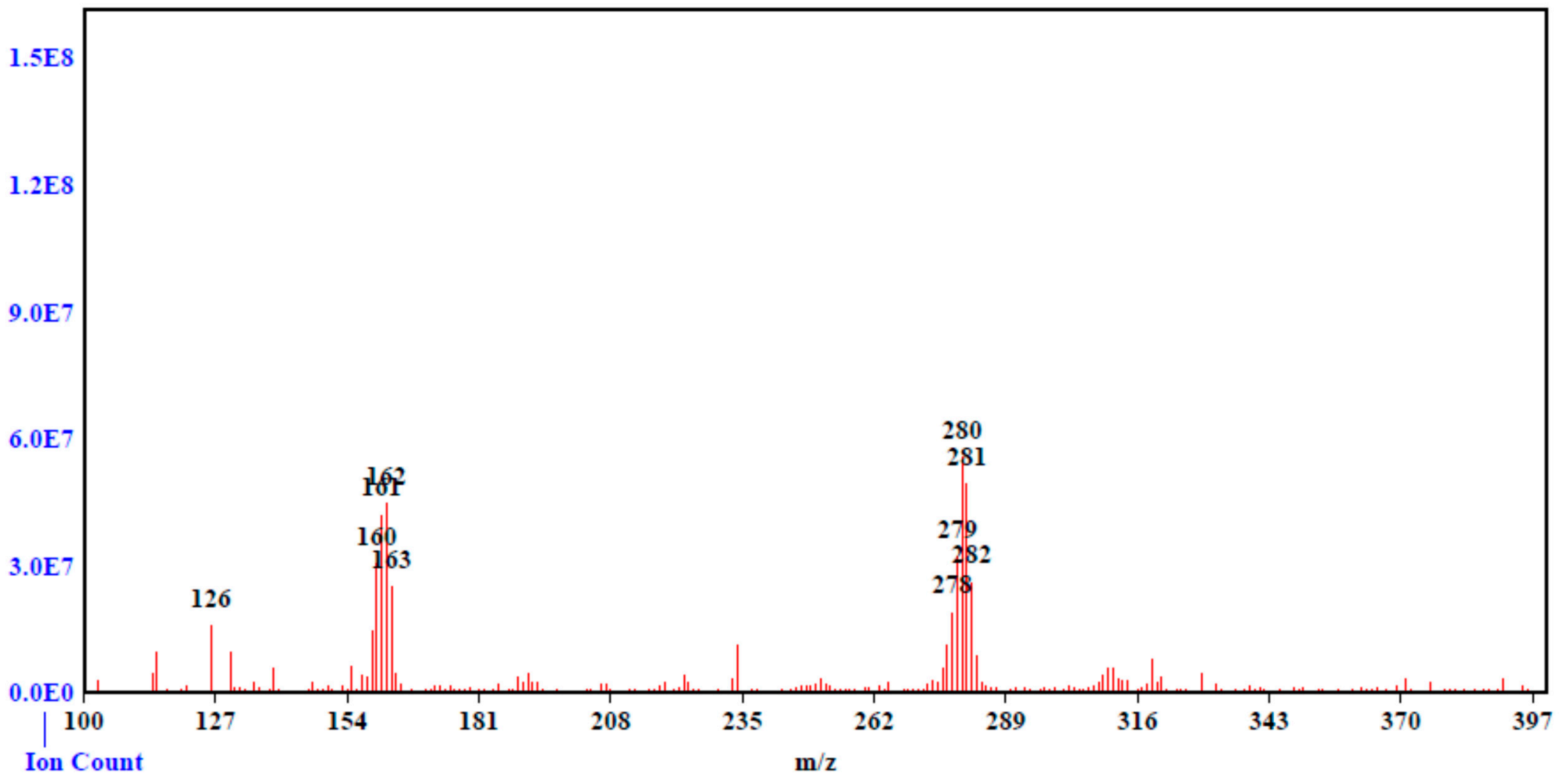

Figure S40. MS spectrum of compound **16**.

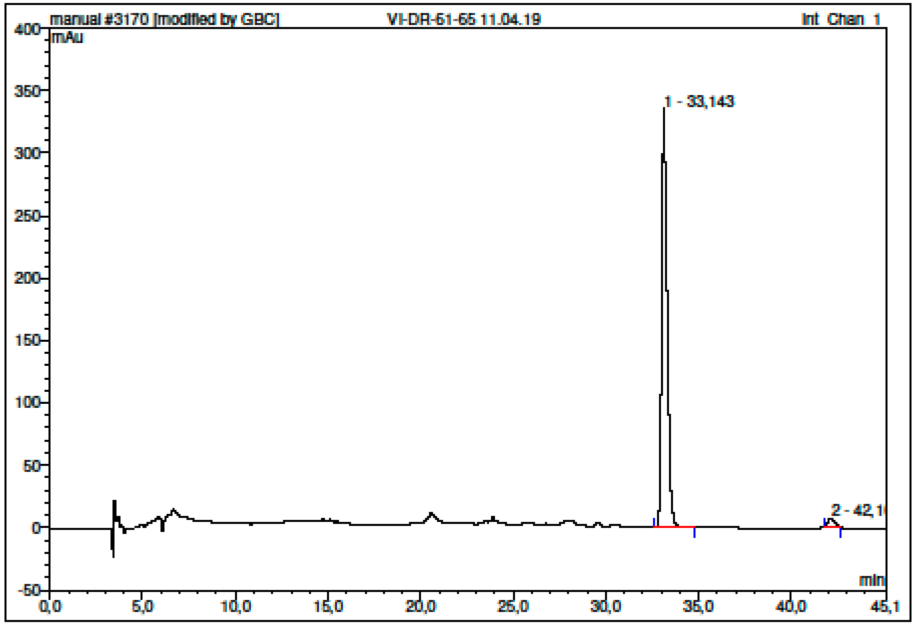

No.	Ret.Time min	Peak Name	Height mAu	Area mAu*min	Rel.Area %	Amount	Type
1	33,14	n.a.	334,957	112,624	97,46	n.a.	BMB
2	42,16	n.a.	6,903	2,935	2,54	n.a.	BMB*
Total:			341,860	115,559	100,00	0,000	

Figure S41. HPLC analysis of compound **16**.

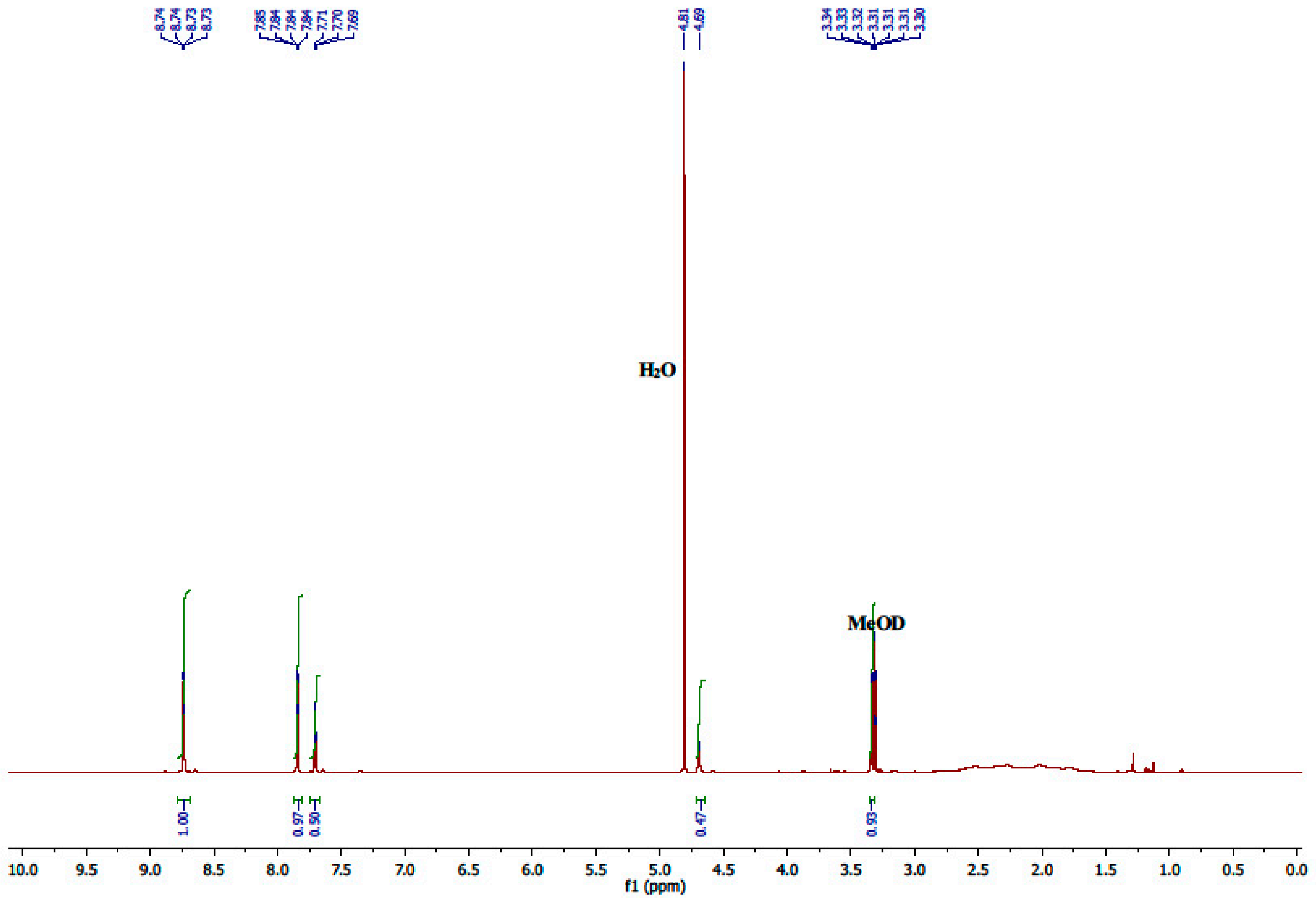

Figure S42. ^{1}H NMR spectrum of compound **20**.

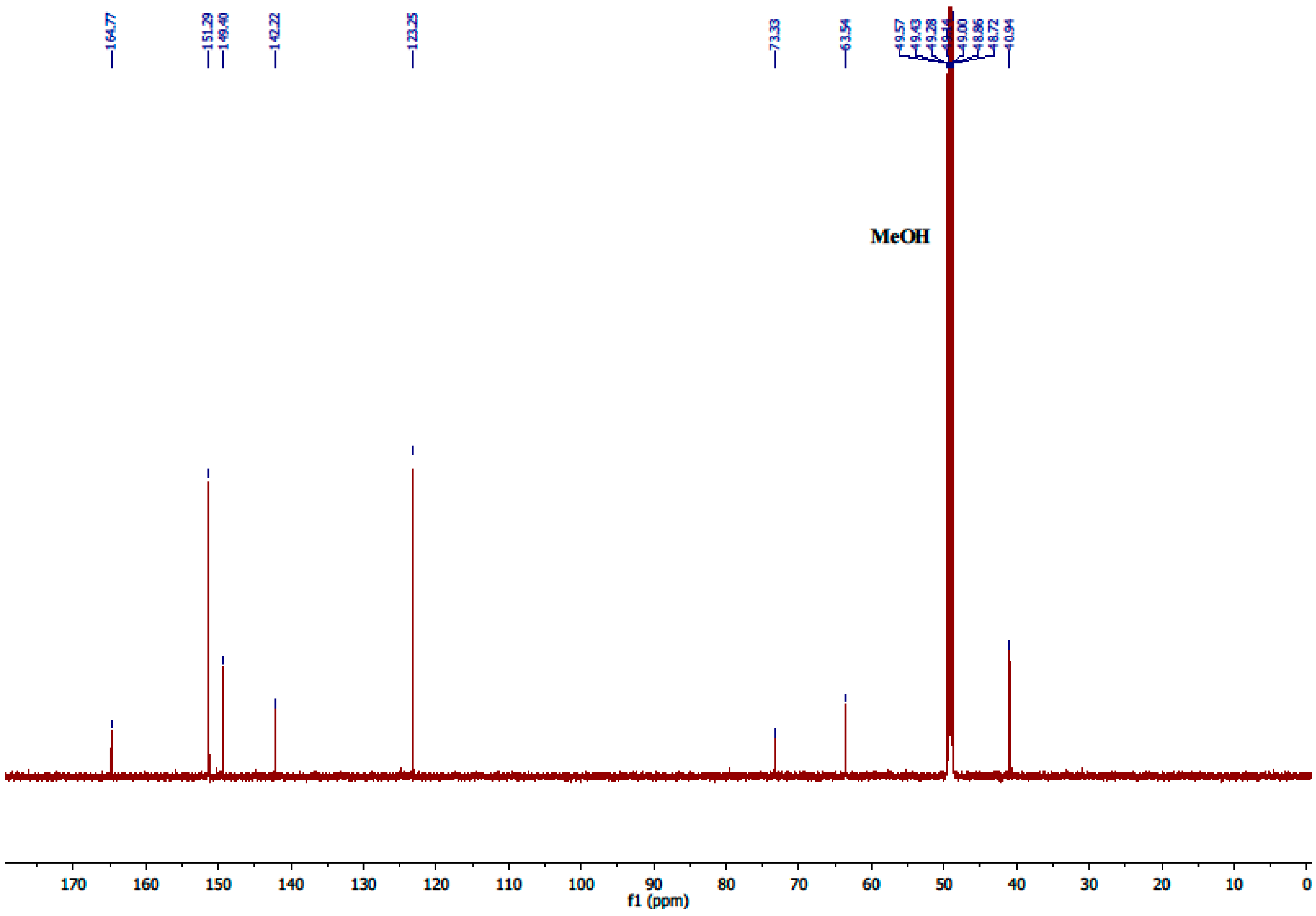

Figure S43. ^{13}C NMR spectrum of compound **20**.

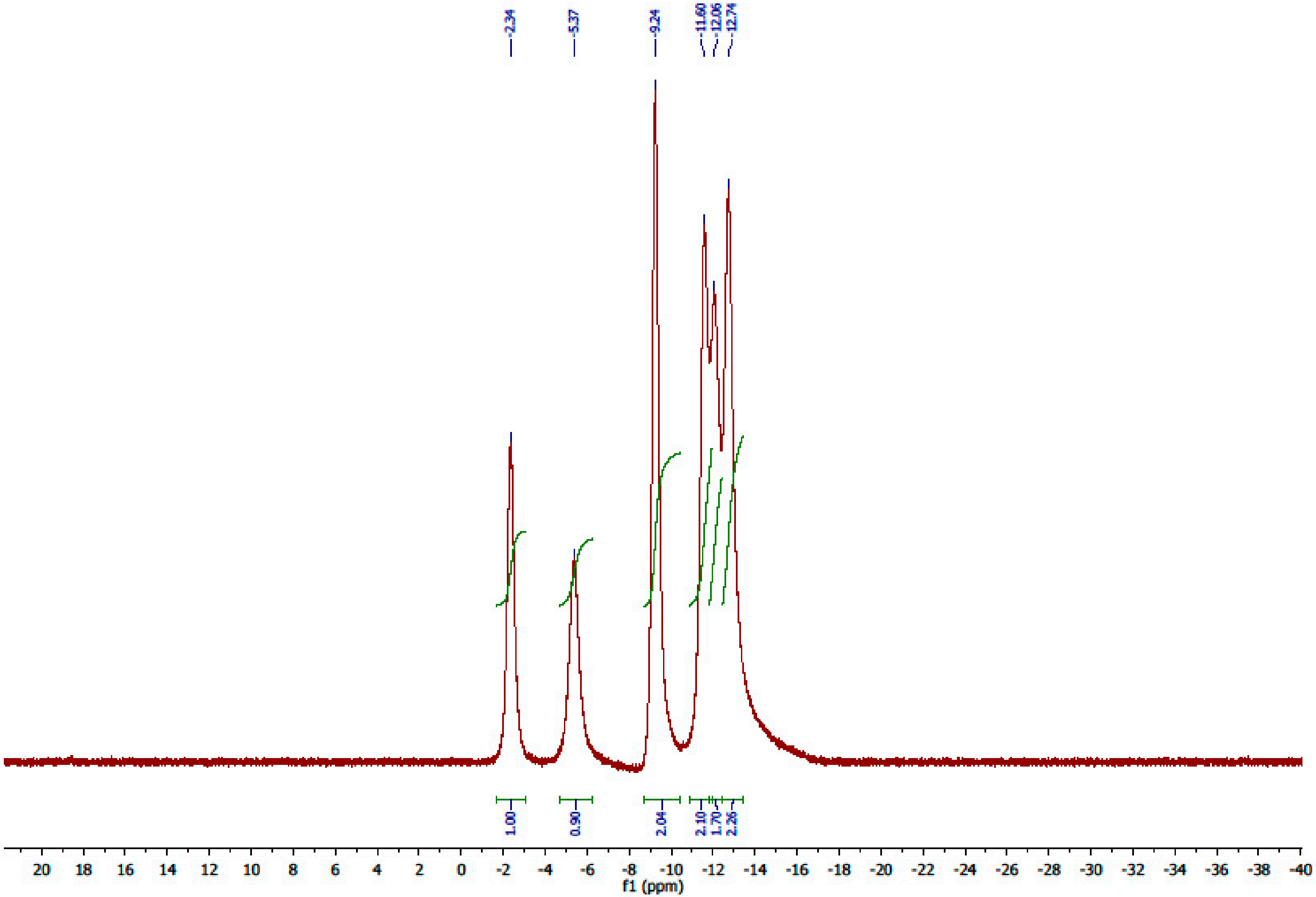

Figure S44. ^{11}B NMR {^{1}H BB} spectrum of compound **20**.

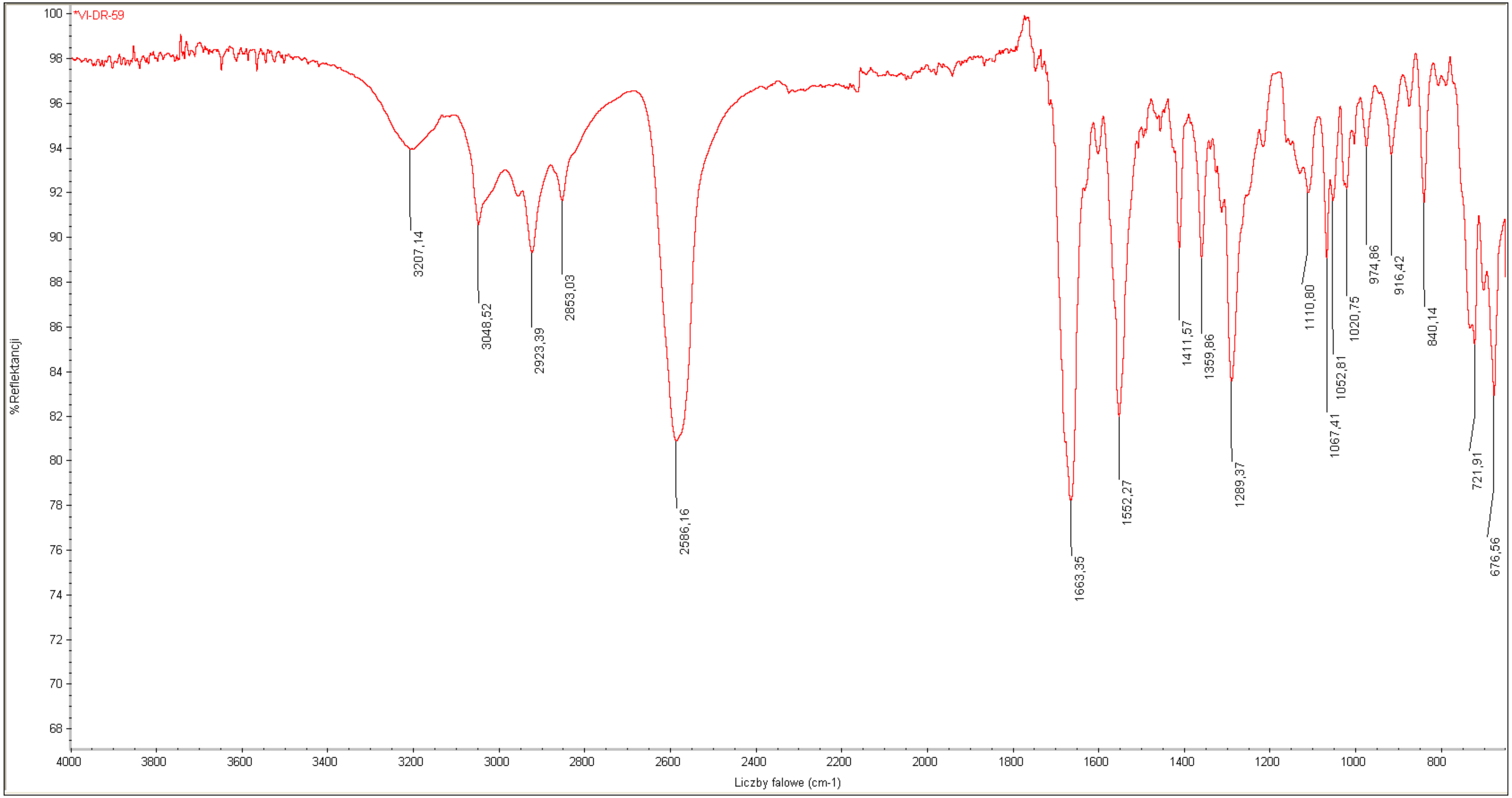

Figure S45. IR spectrum of compound **20**.

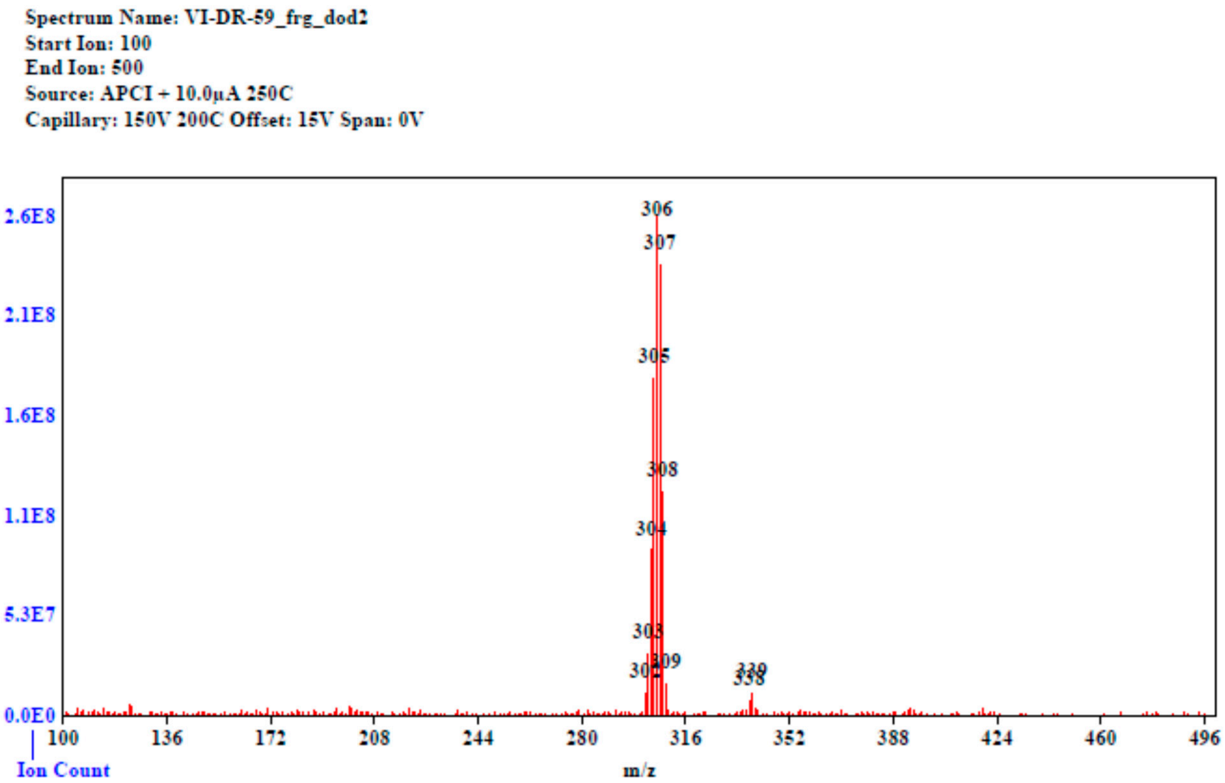

Figure S46. MS spectrum of compound **20**.

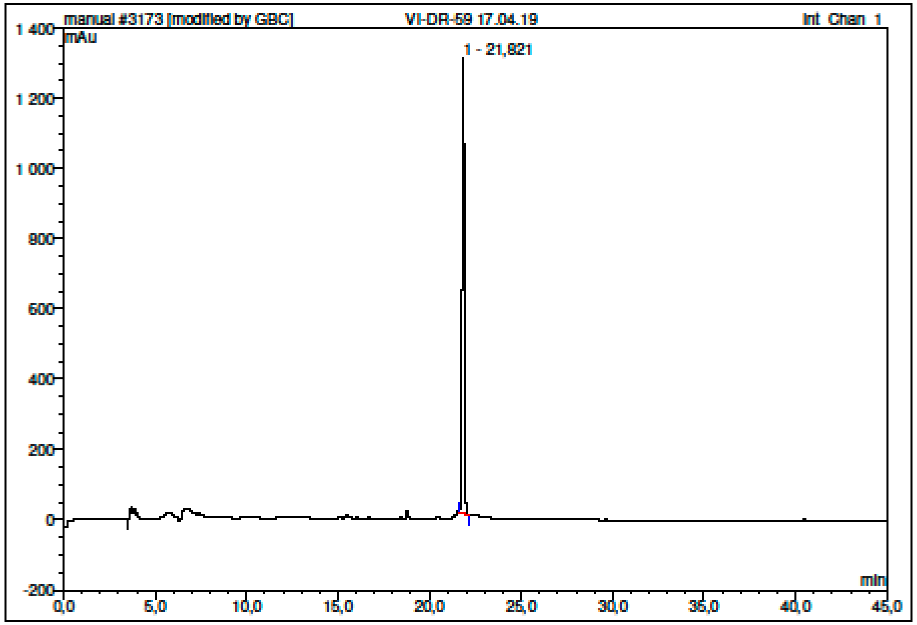

No.	Ret.Time min	Peak Name	Height mAu	Area mAu*min	Rel.Area %	Amount	Type
1	21,82	n.a.	1296,900	170,826	100,00	n.a.	BMB
Total:			1296,900	170,826	100,00	0,000	

Figure S47. HPLC analysis of compound **20**.

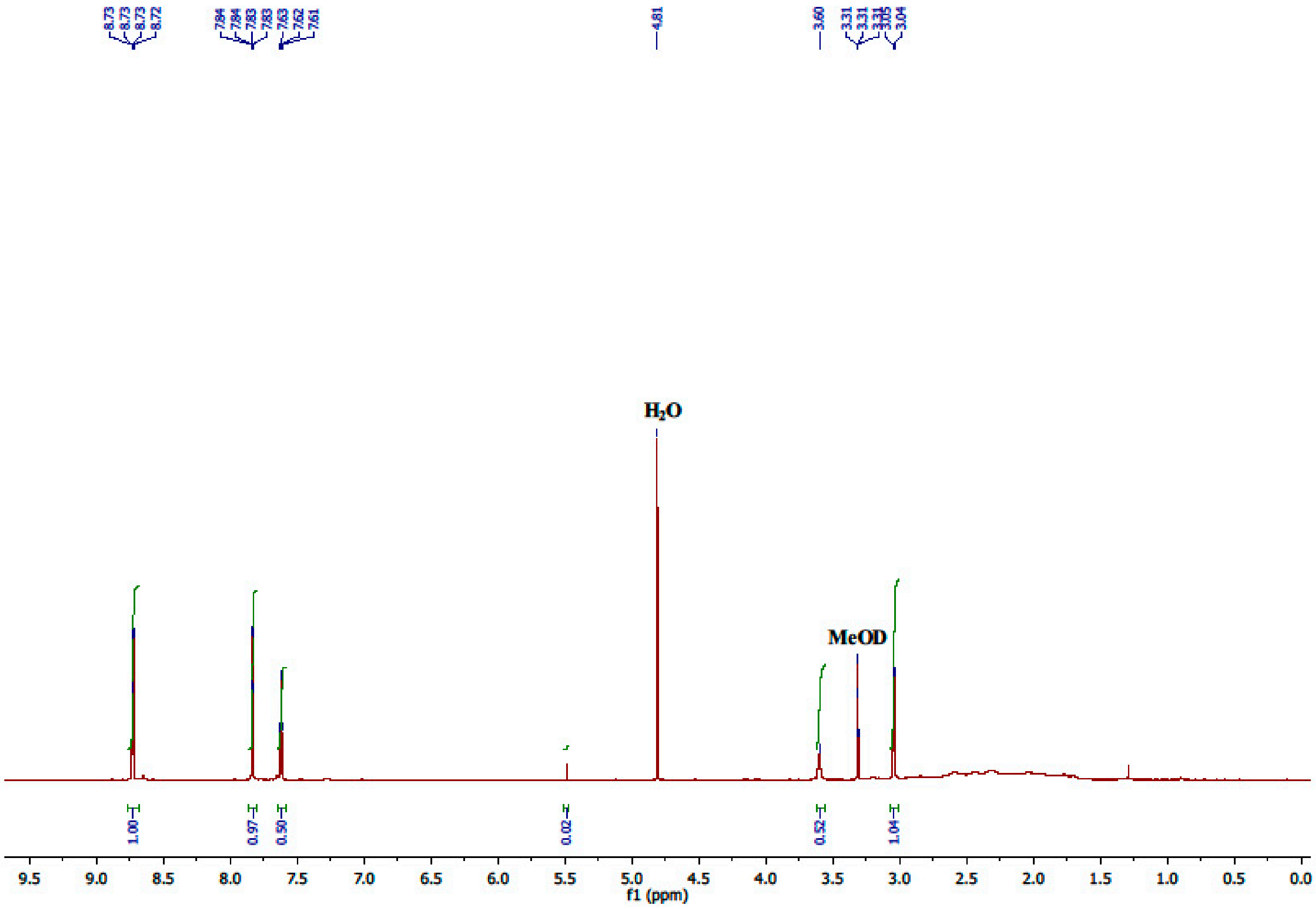

Figure S48. ^{1}H NMR spectrum of compound **21**.

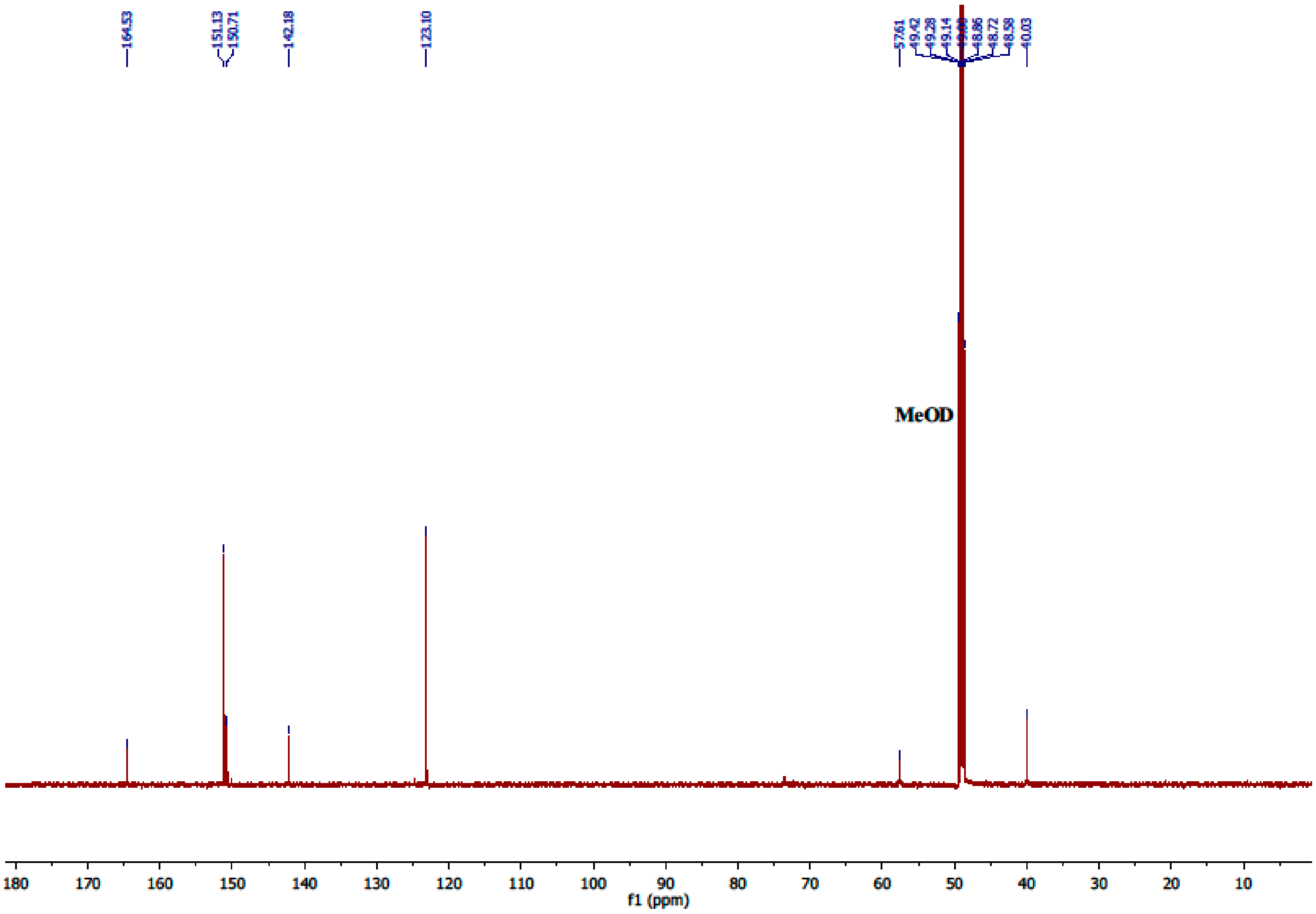

Figure S49. ^{13}C NMR spectrum of compound **21**.

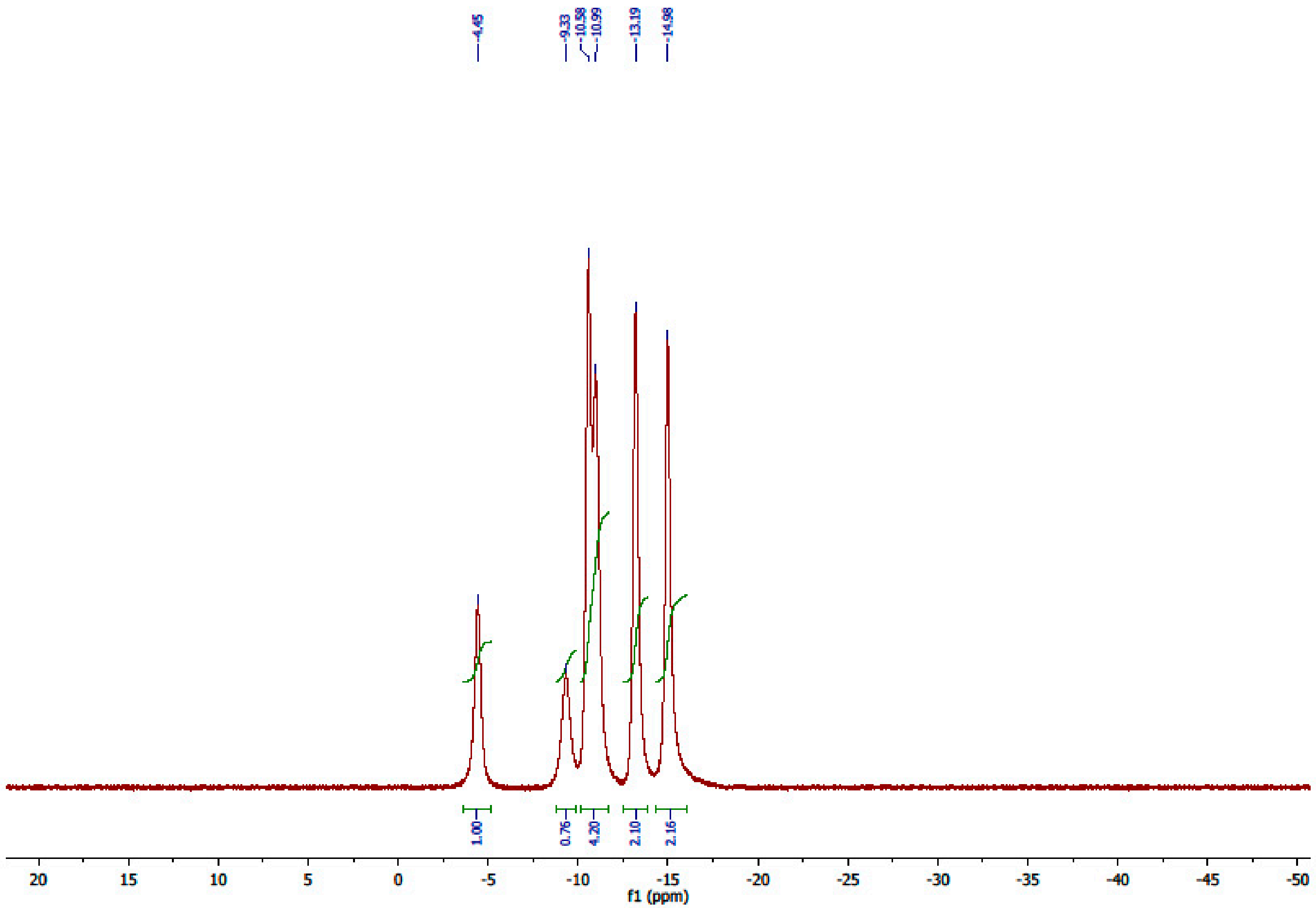

Figure S50. ^{11}B NMR {^{1}H BB} spectrum of compound **21**.

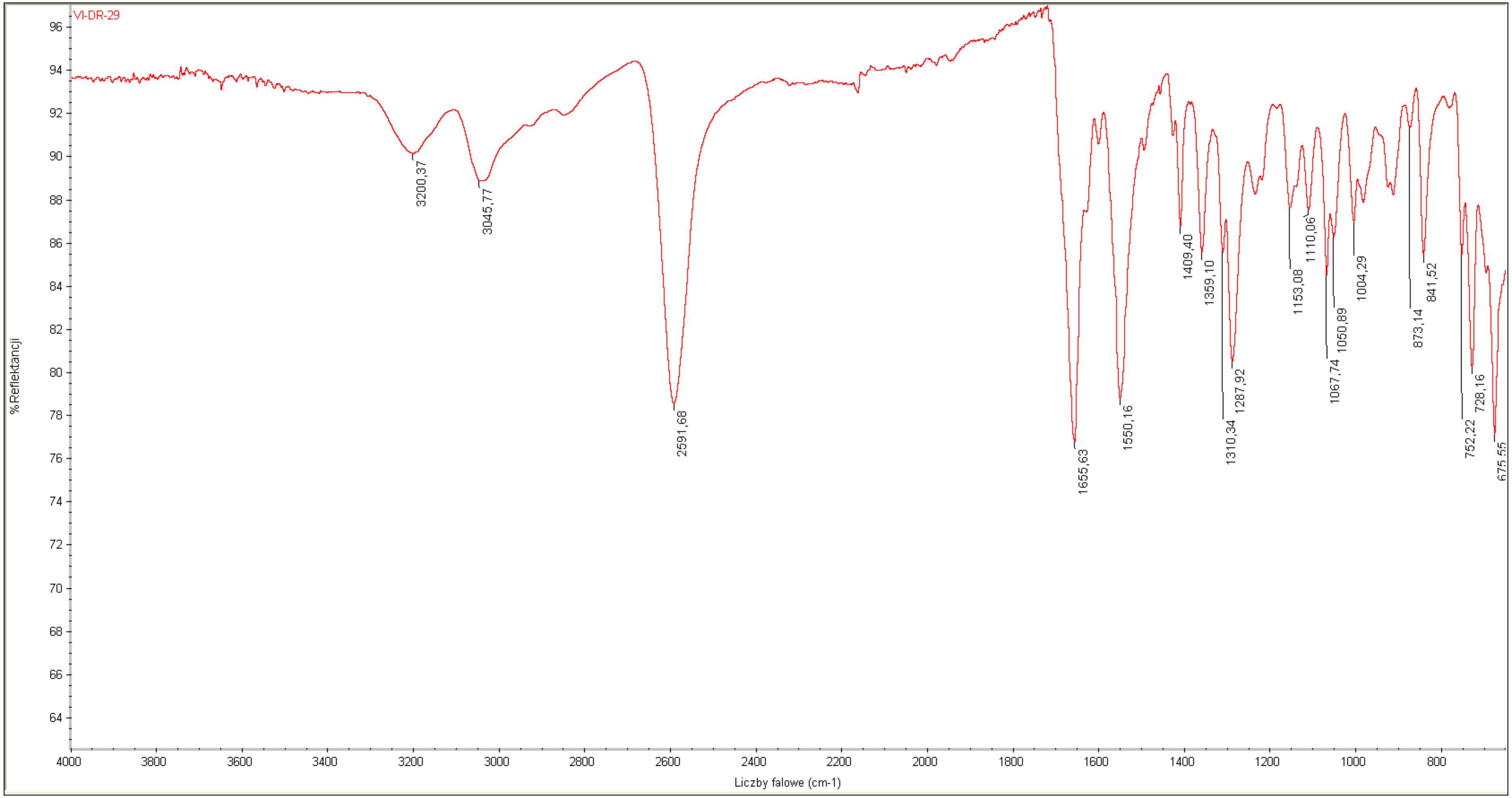

Figure S51. IR spectrum of **21**.

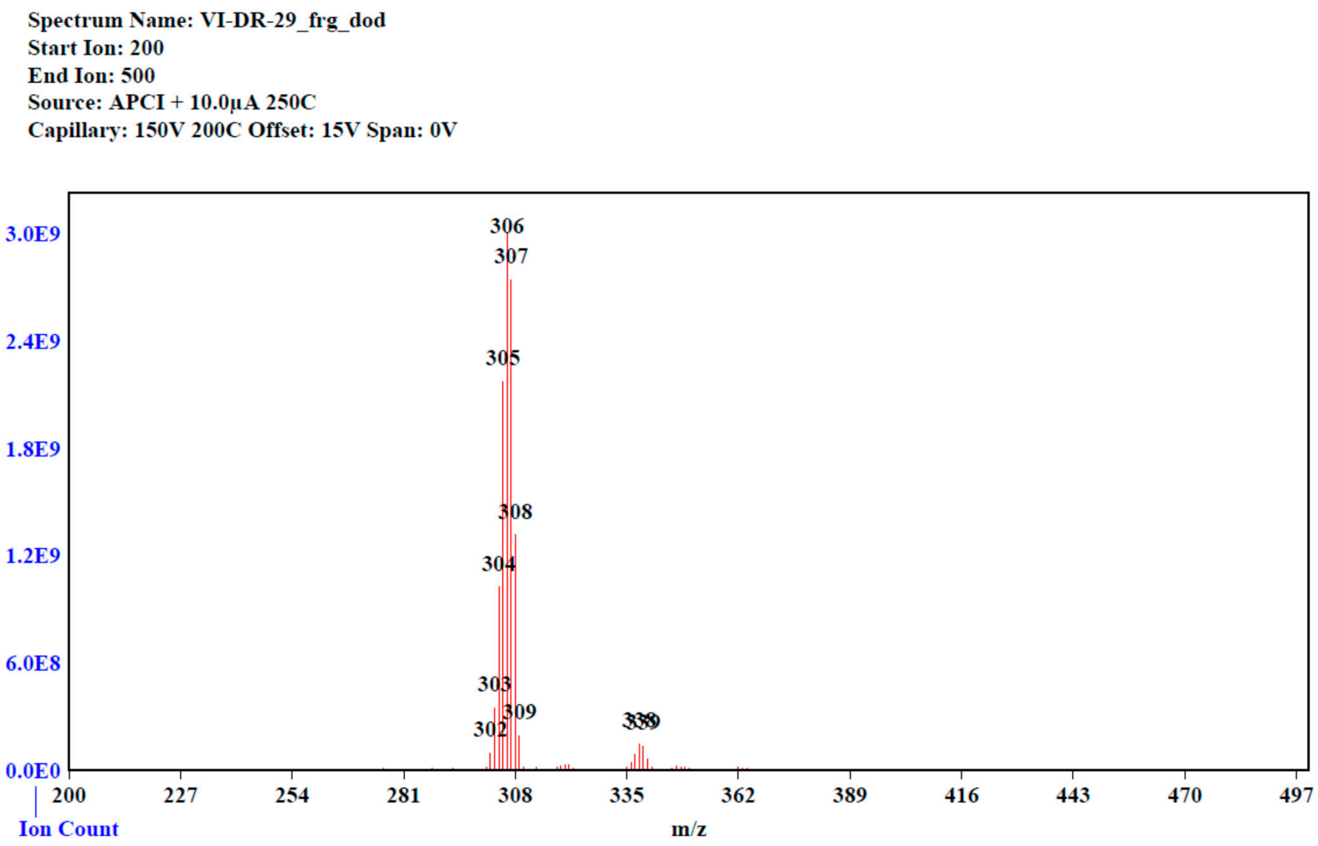

Figure S52. MS spectrum of compound **21**.

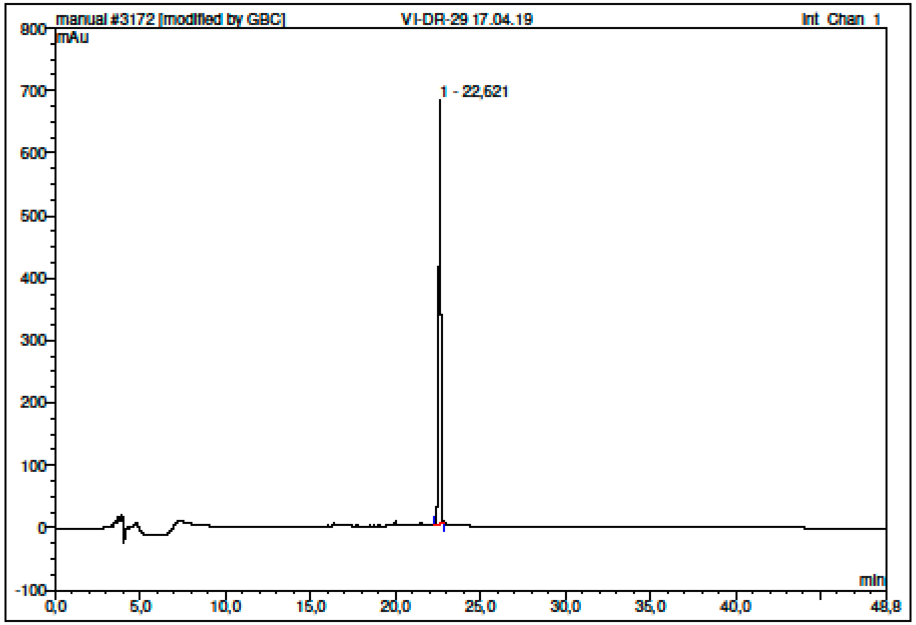

No.	Ret.Time min	Peak Name	Height mAu	Area mAu*min	Rel.Area %	Amount	Type
1	22,62	n.a.	678,063	95,957	100,00	n.a.	BMB
Total:			678,063	95,957	100,00	0,000	

Figure S53. HPLC analysis of compound **21**.

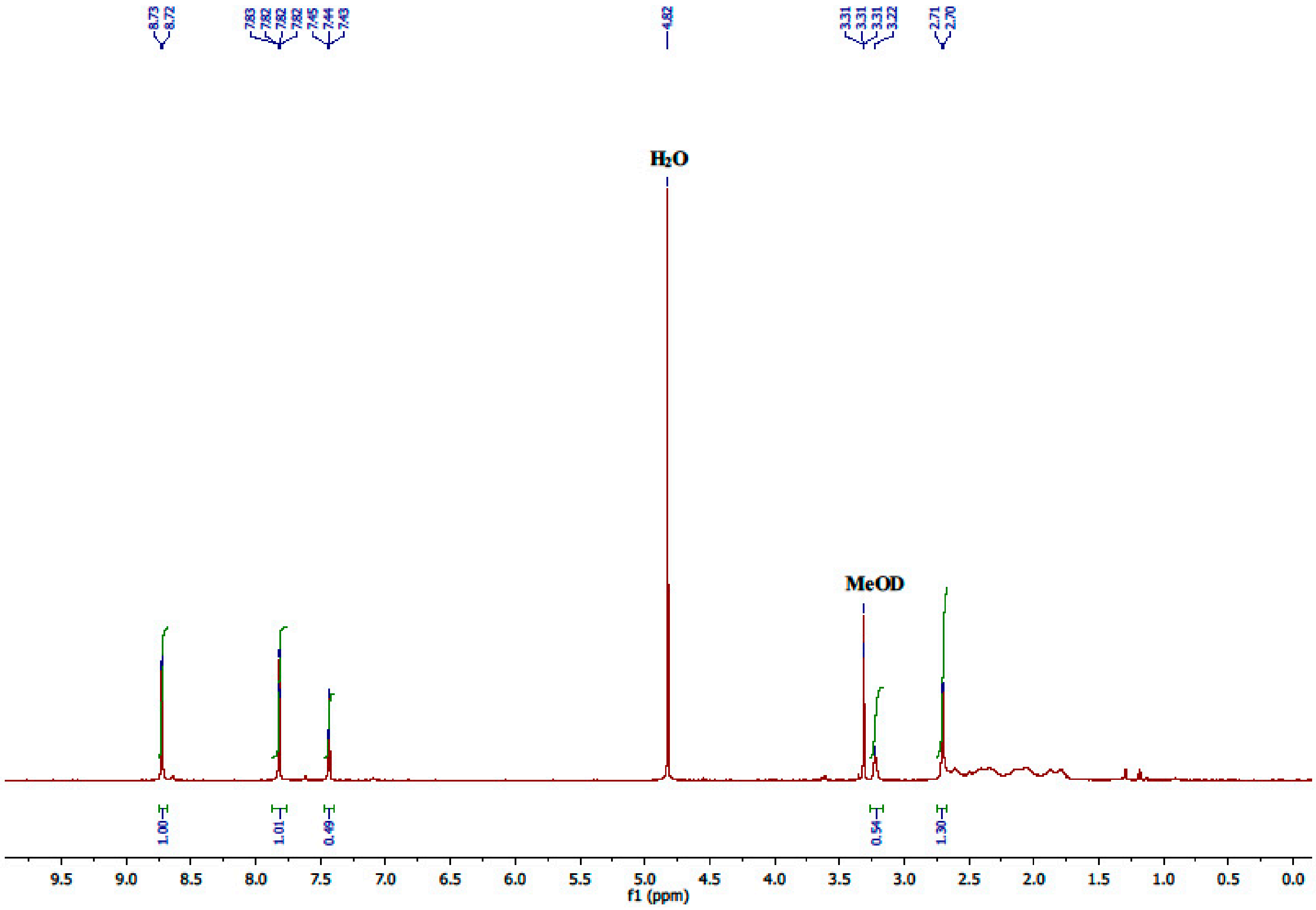

Figure S54. ^{1}H NMR spectrum of compound **22**.

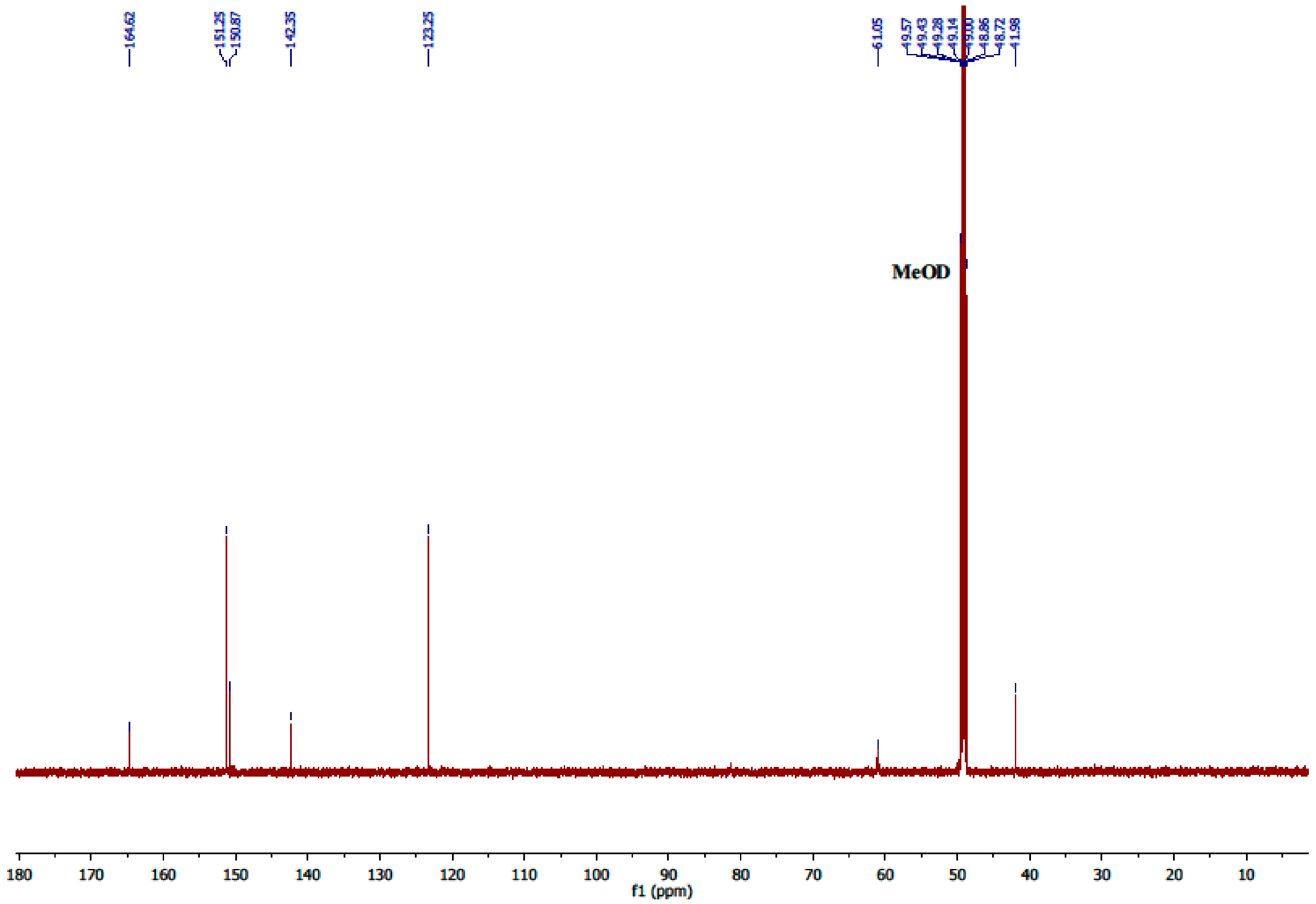

Figure S55. ^{13}C NMR spectrum of compound **22**.

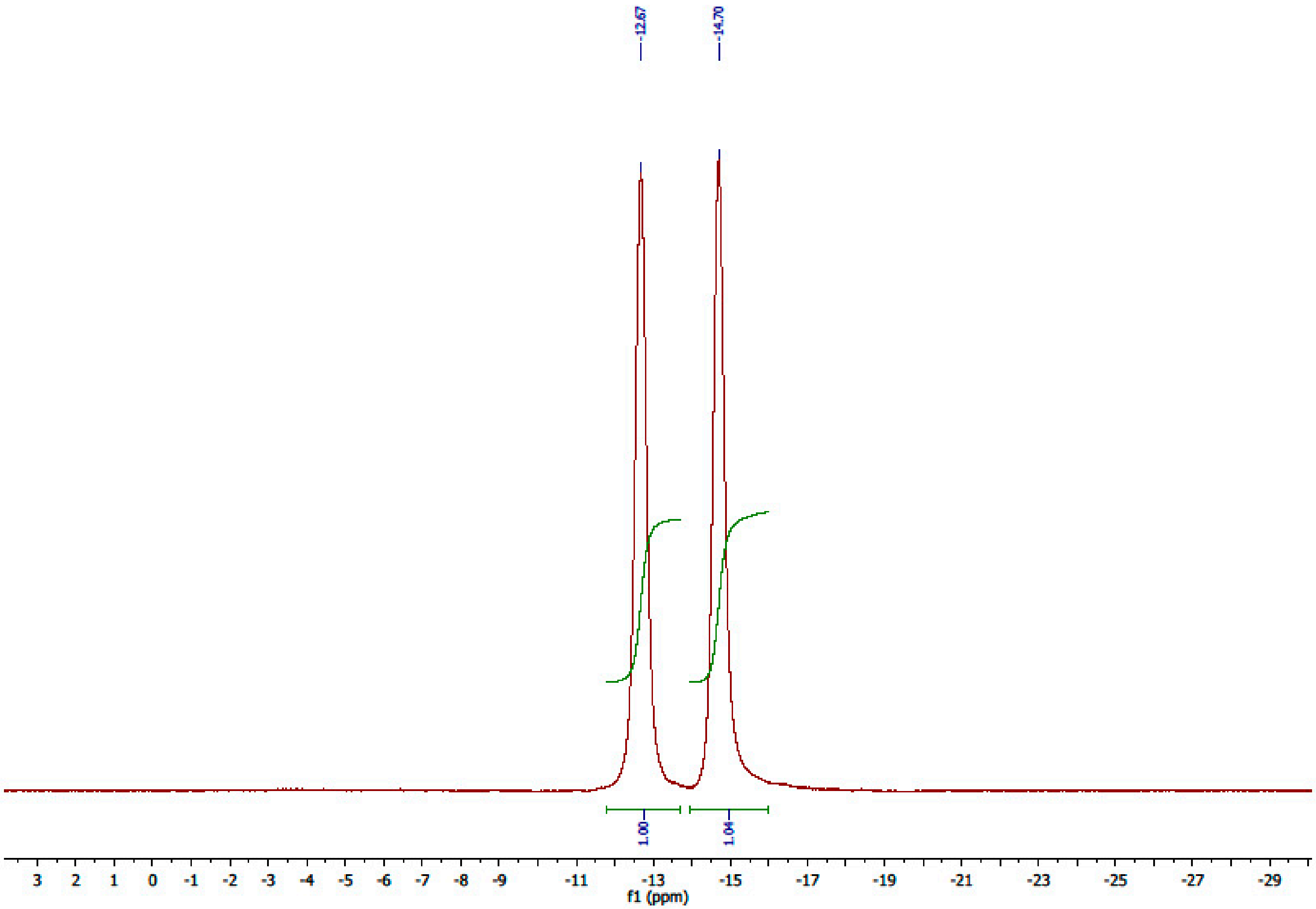

Figure S56. ^{11}B NMR {^{1}H BB} spectrum of compound **22**.

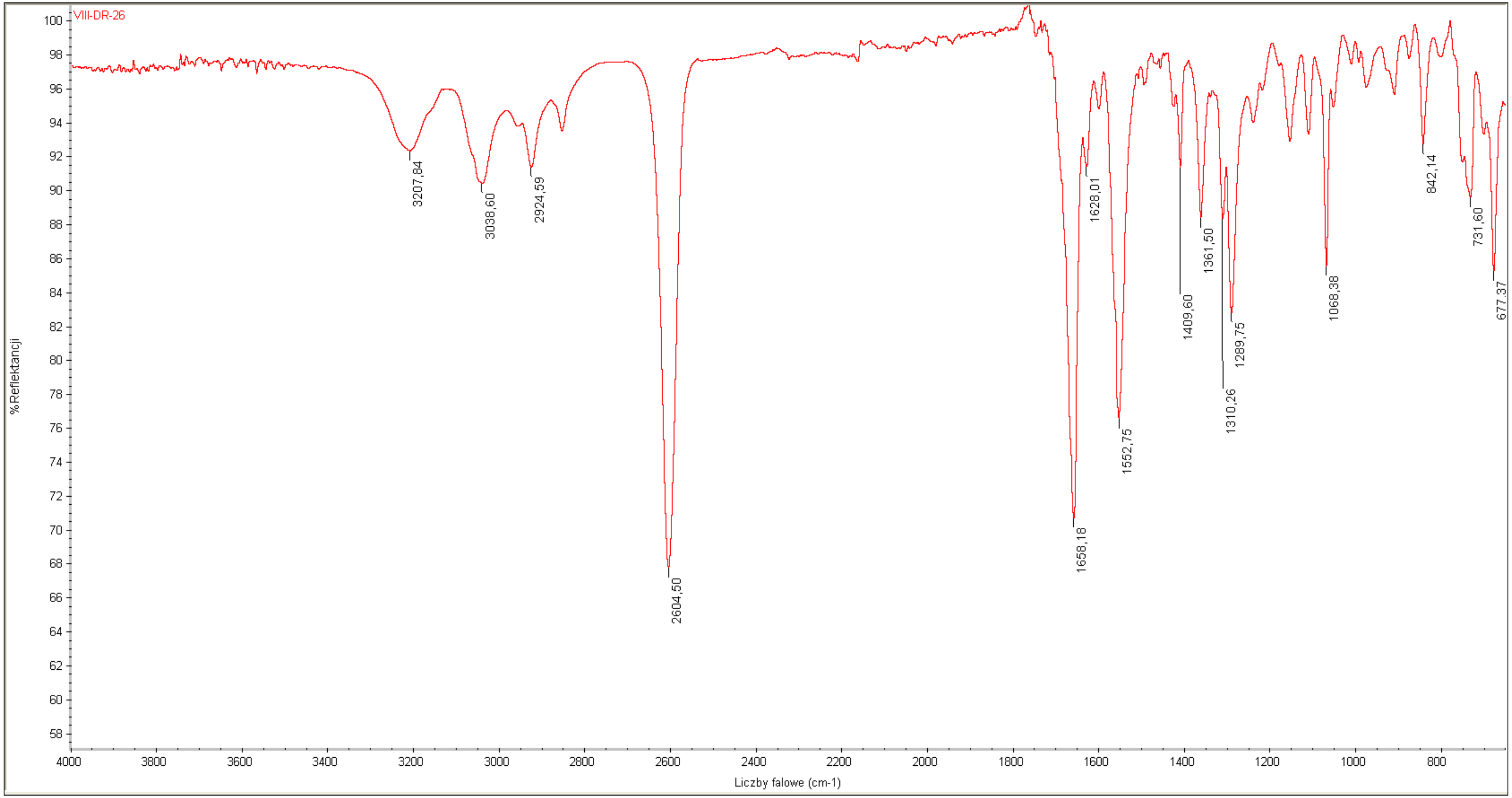

Figure S57. IR spectrum of compound **22**.

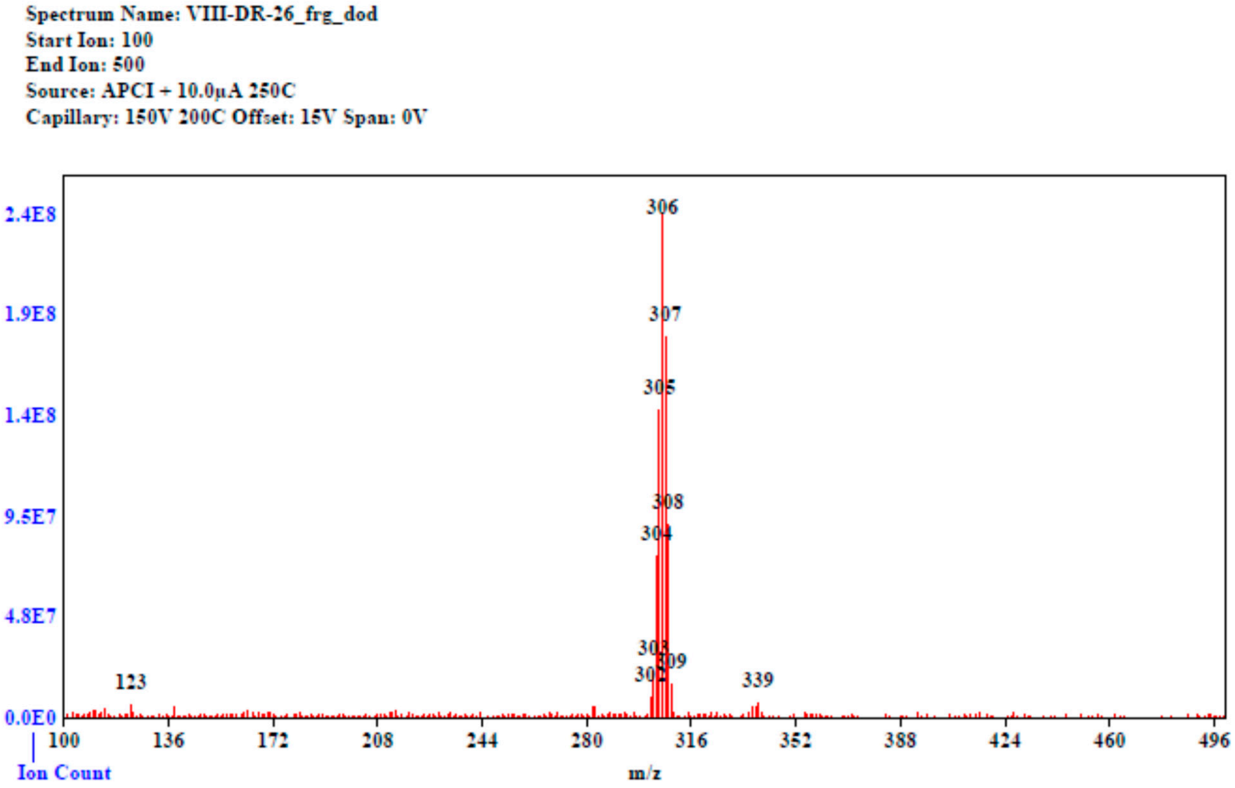

Figure S58. MS spectrum of compound **22**.

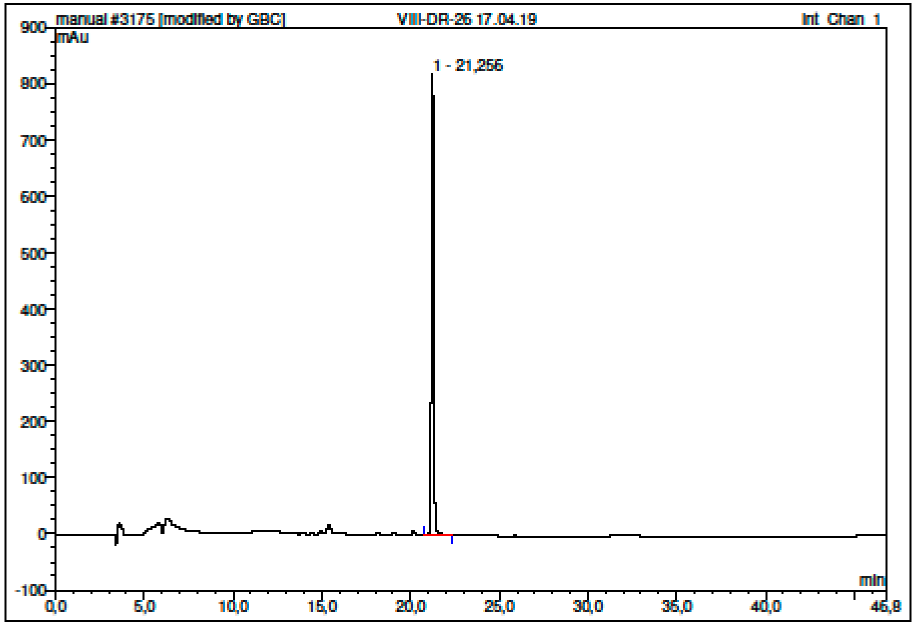

No.	Ret.Time min	Peak Name	Height mAu	Area mAu*min	Rel.Area %	Amount	Type
1	21,26	n.a.	820,802	117,104	100,00	n.a.	BMB
Total:			820,802	117,104	100,00	0,000	

Figure S59. HPLC analysis of compound **22**.

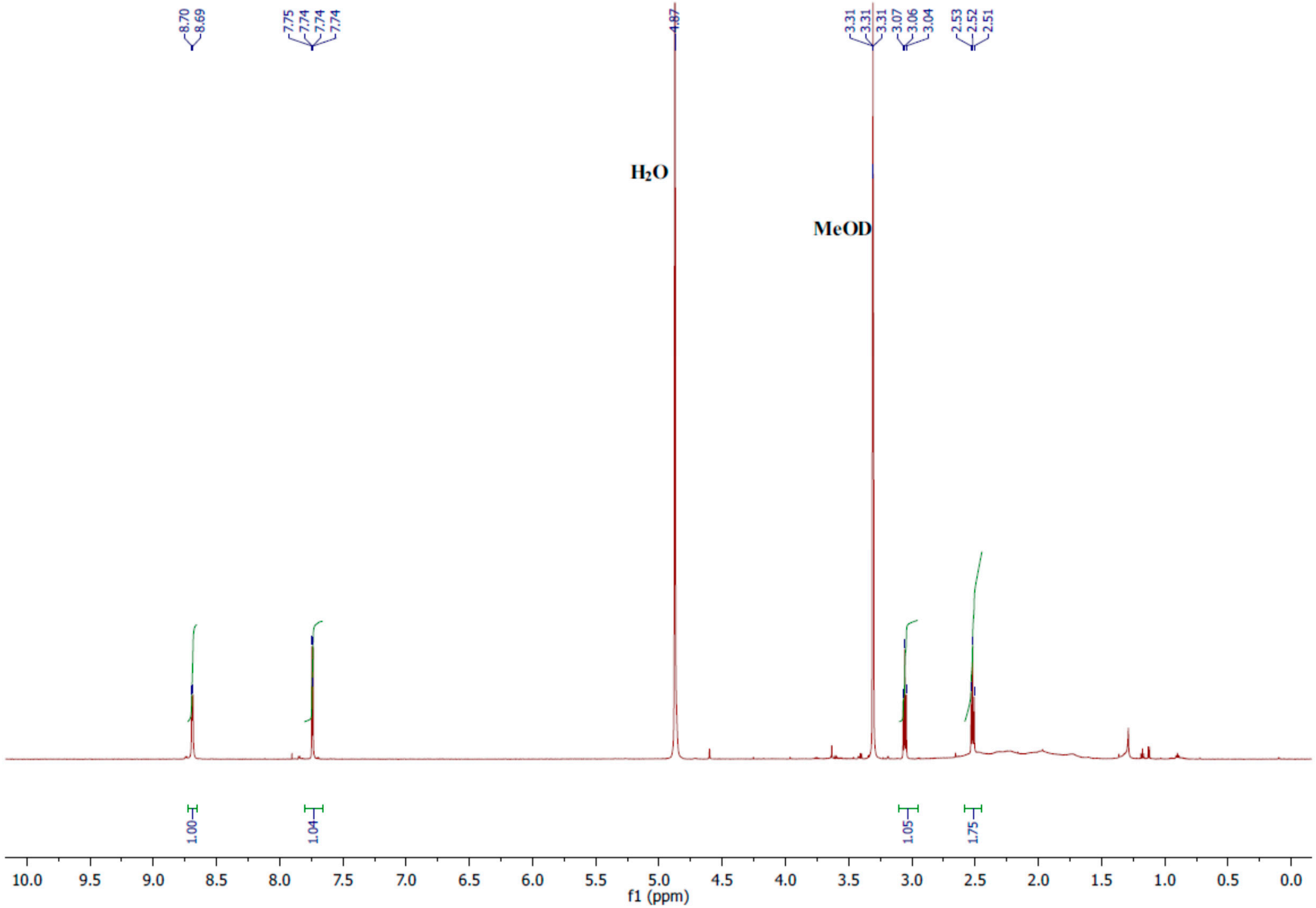

Figure S60. ^{1}H NMR spectrum of compound **23**.

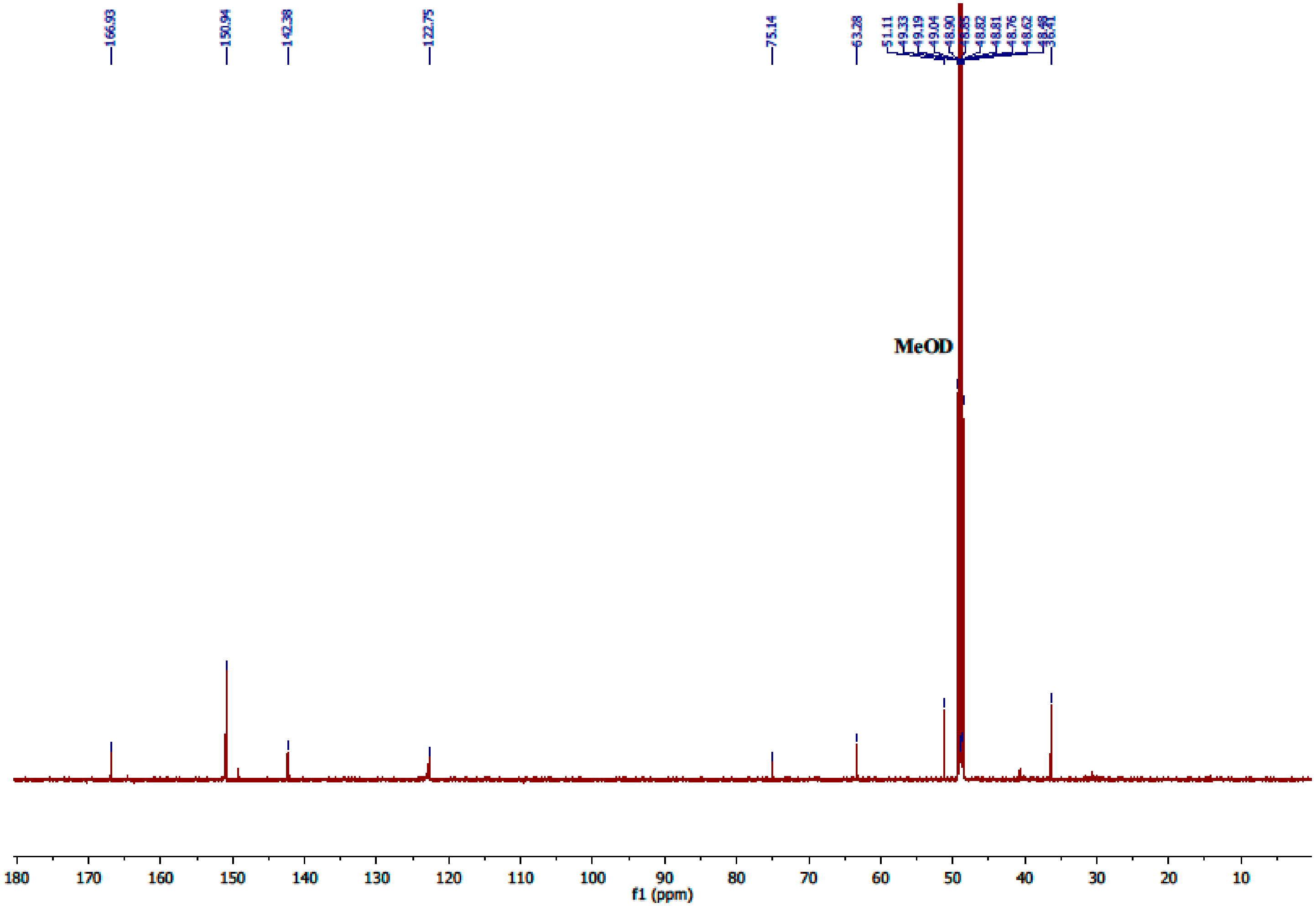

Figure S61. ^{13}C NMR spectrum of compound **23**.

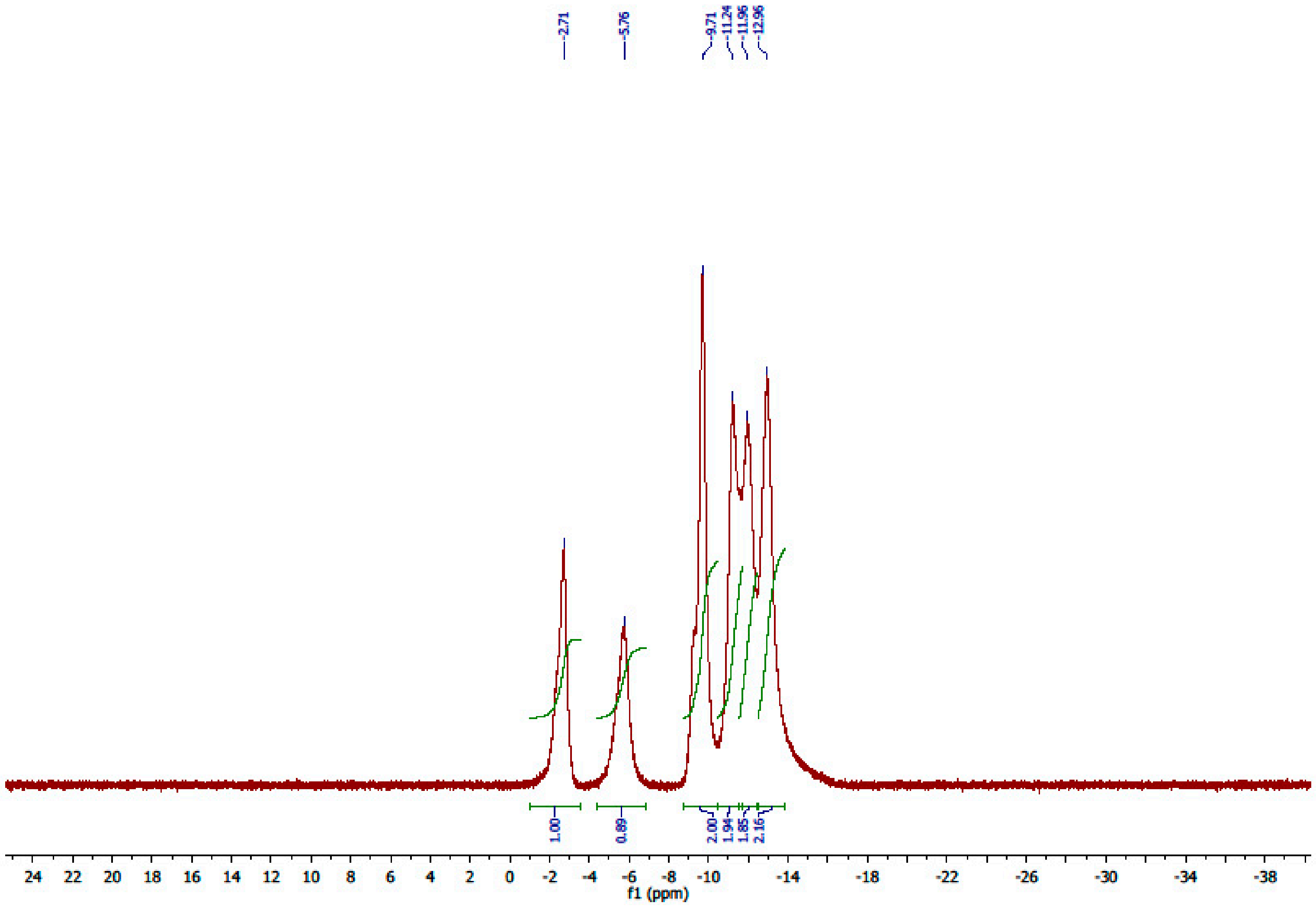

Figure S62. ^{11}B NMR {^{1}H BB} spectrum of compound **23**.

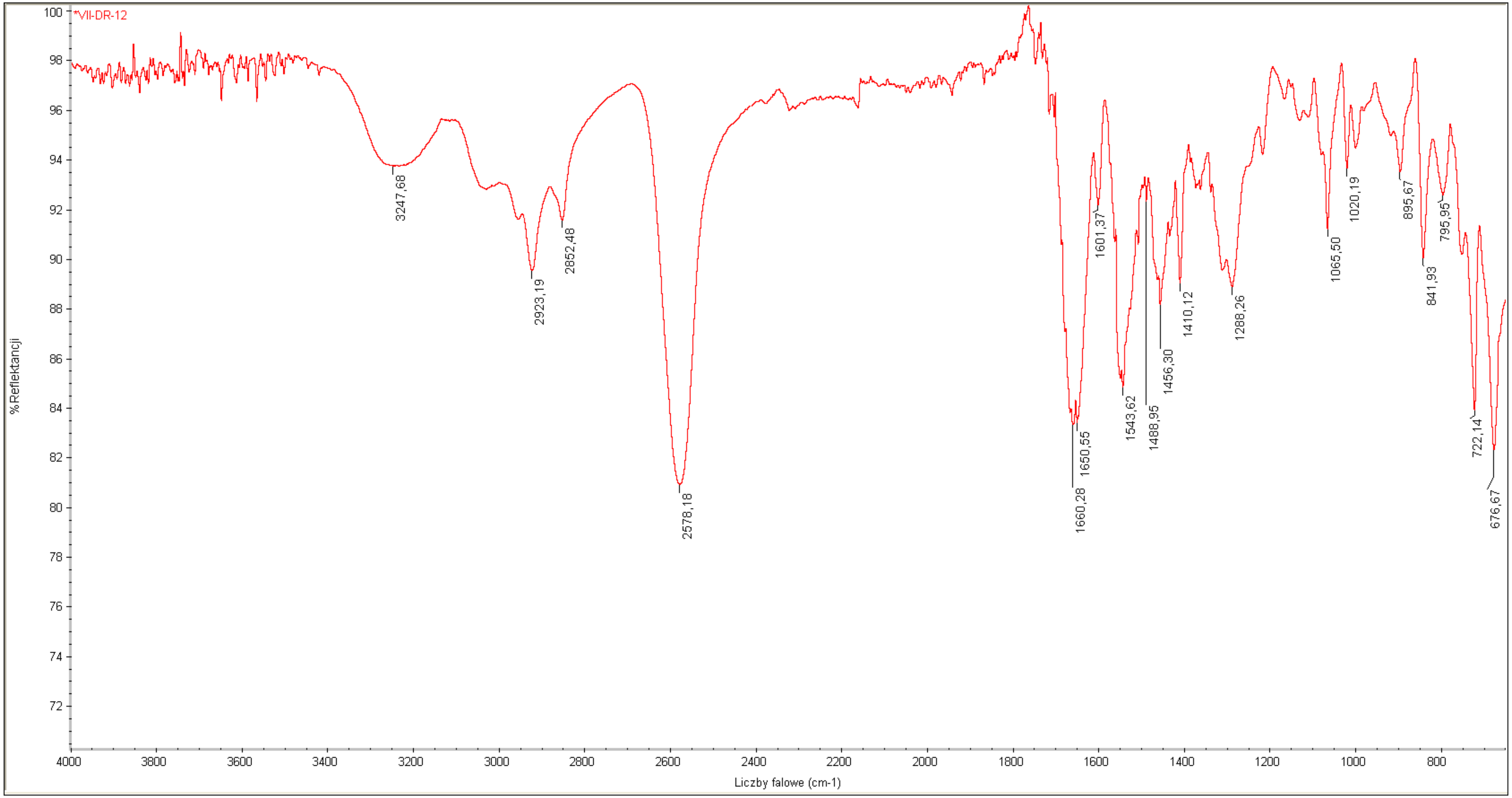

Figure S63. IR spectrum of compound **23**.

Spectrum Name: VII-DR-12_rob_dod
Start Ion: 100
End Ion: 500
Source: APCI + 10.0µA 400C
Capillary: 180V 300C Offset: 30V Span: 20V

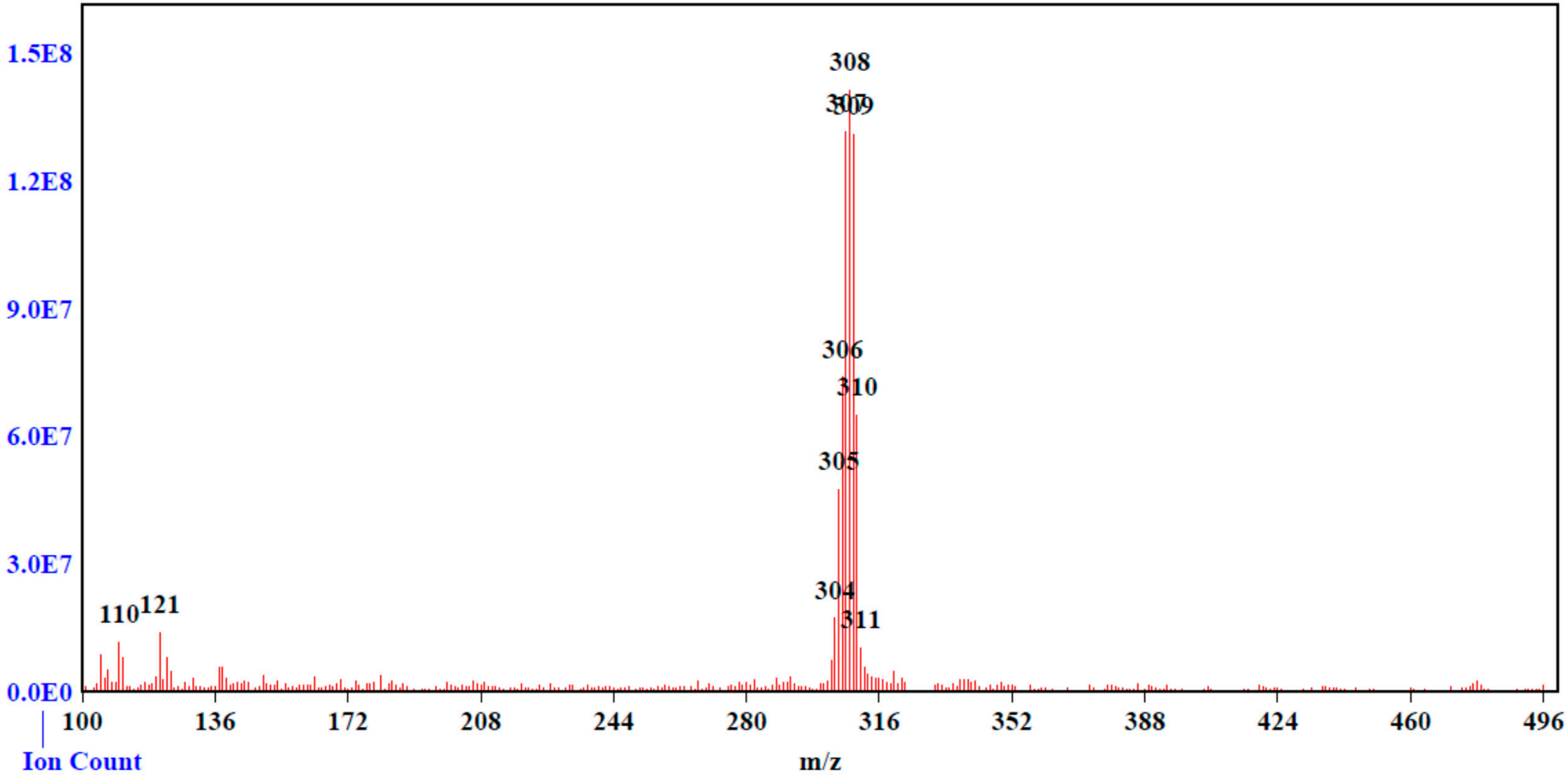

Figure S64. MS spectrum of compound **23**.

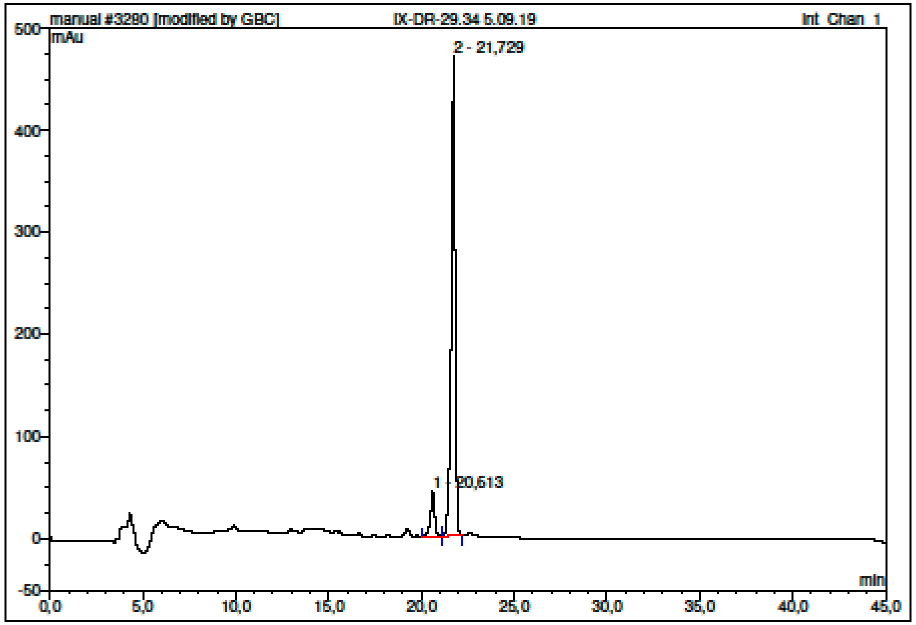

No.	Ret.Time min	Peak Name	Height mAu	Area mAu*min	Rel.Area %	Amount	Type
1	20,61	n.a.	43,971	11,151	8,86	n.a.	BM
2	21,73	n.a.	469,778	114,700	91,14	n.a.	MB
Total:			513,750	125,851	100,00	0,000	

Figure S65. HPLC analysis of compound **23**.

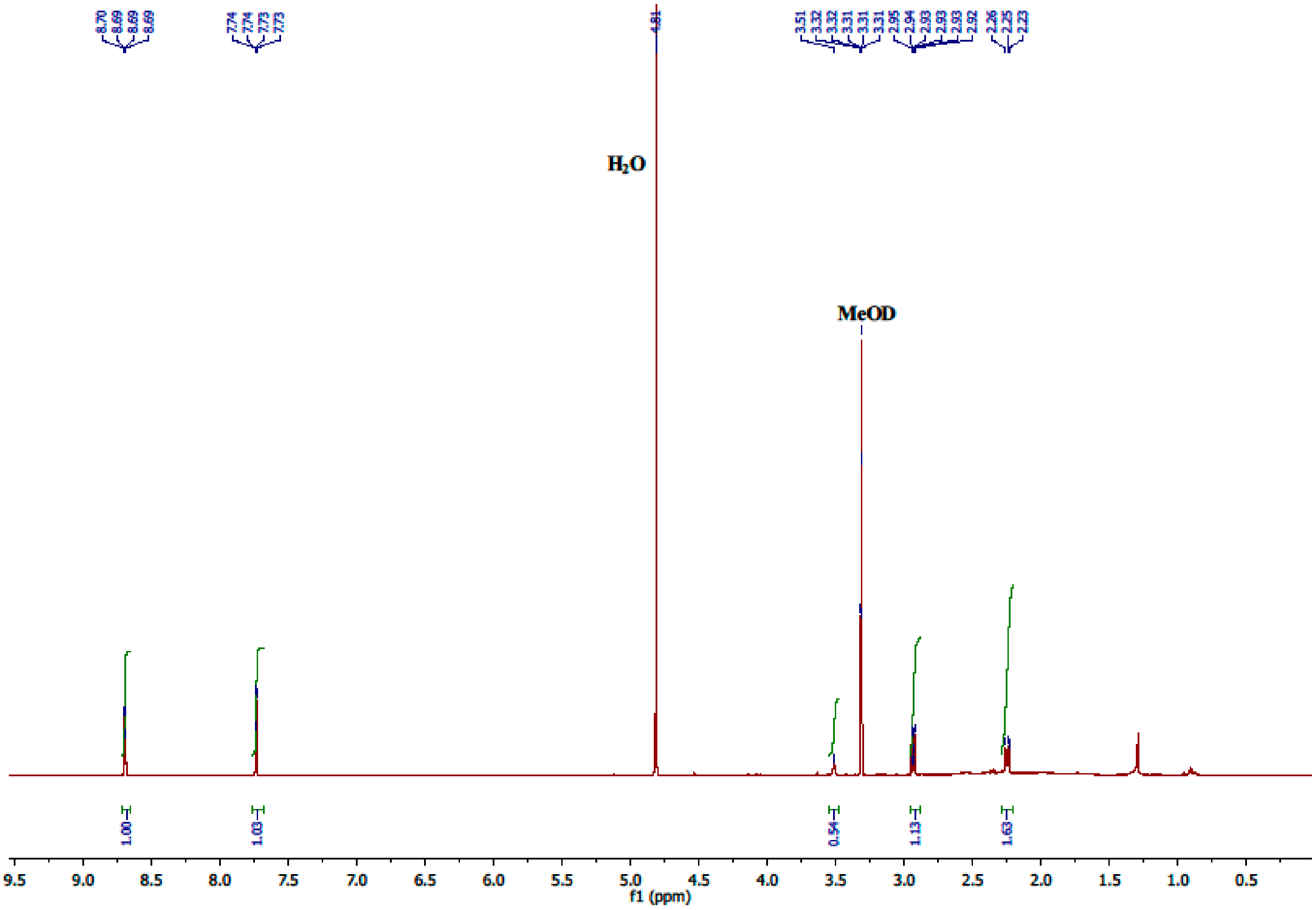

Figure S66. ^{1}H NMR spectrum of compound **24**.

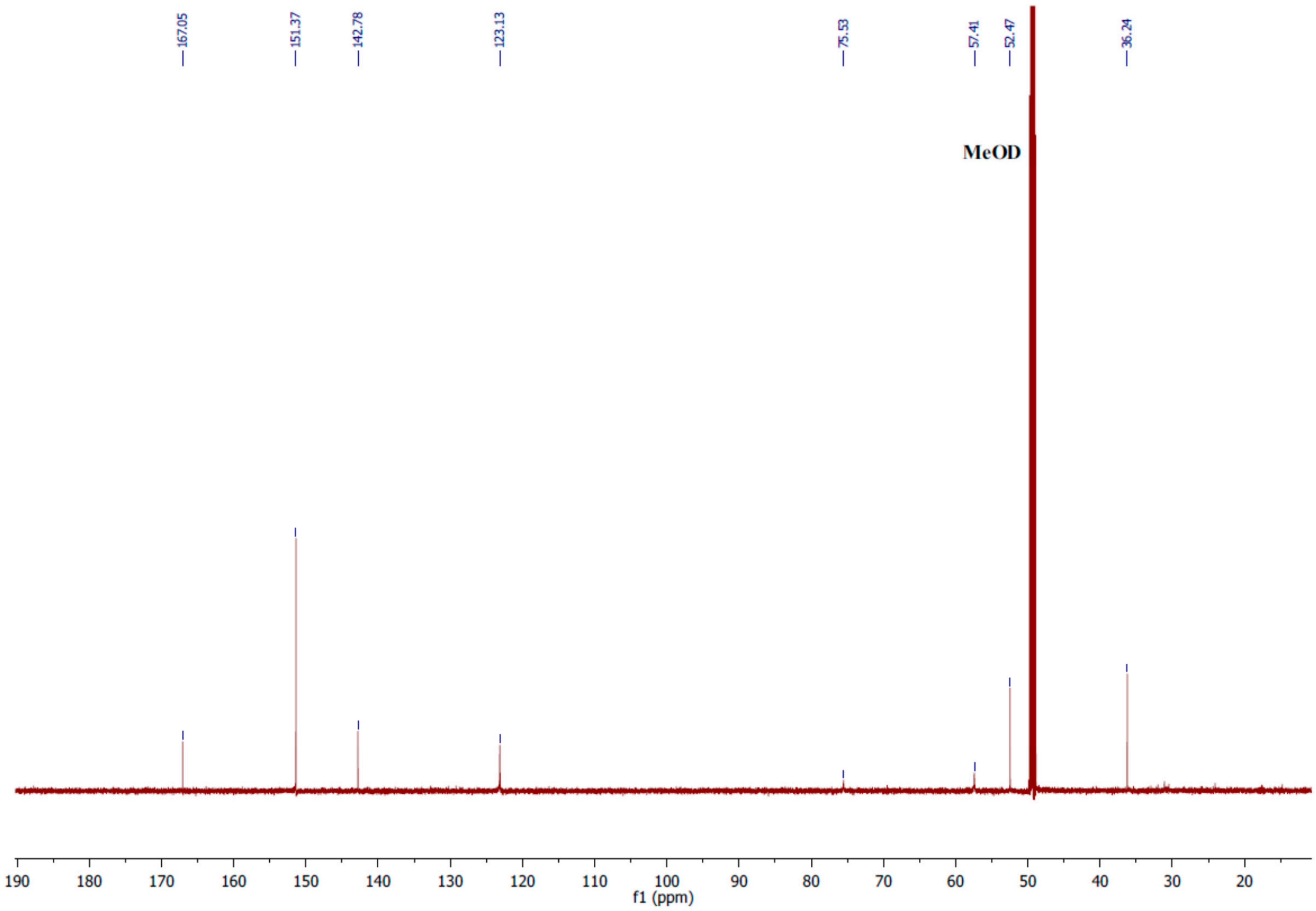

Figure S67. ^{13}C NMR spectrum of compound **24**.

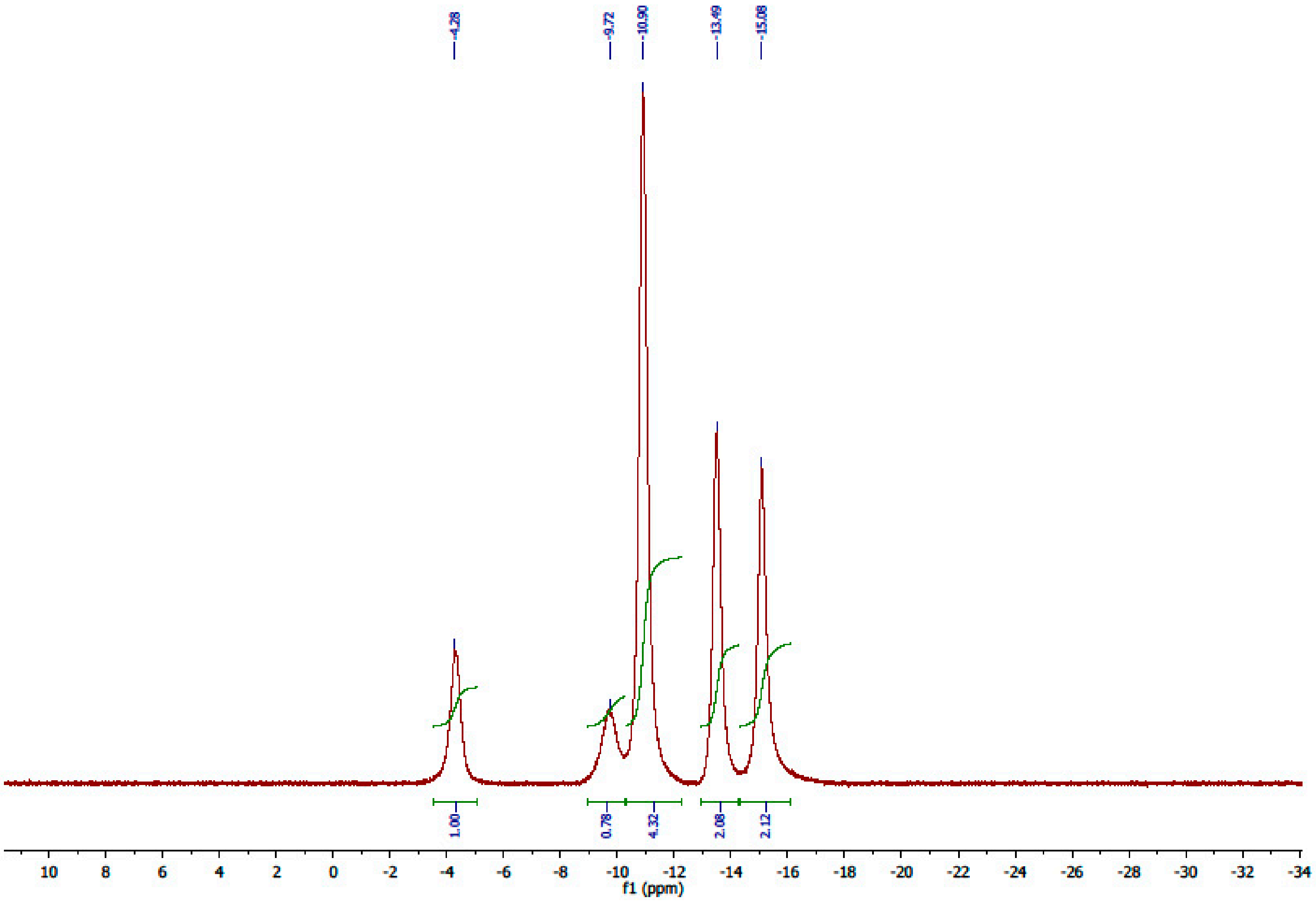

Figure S68. ^{11}B NMR {^{1}H BB} spectrum of compound **24**.

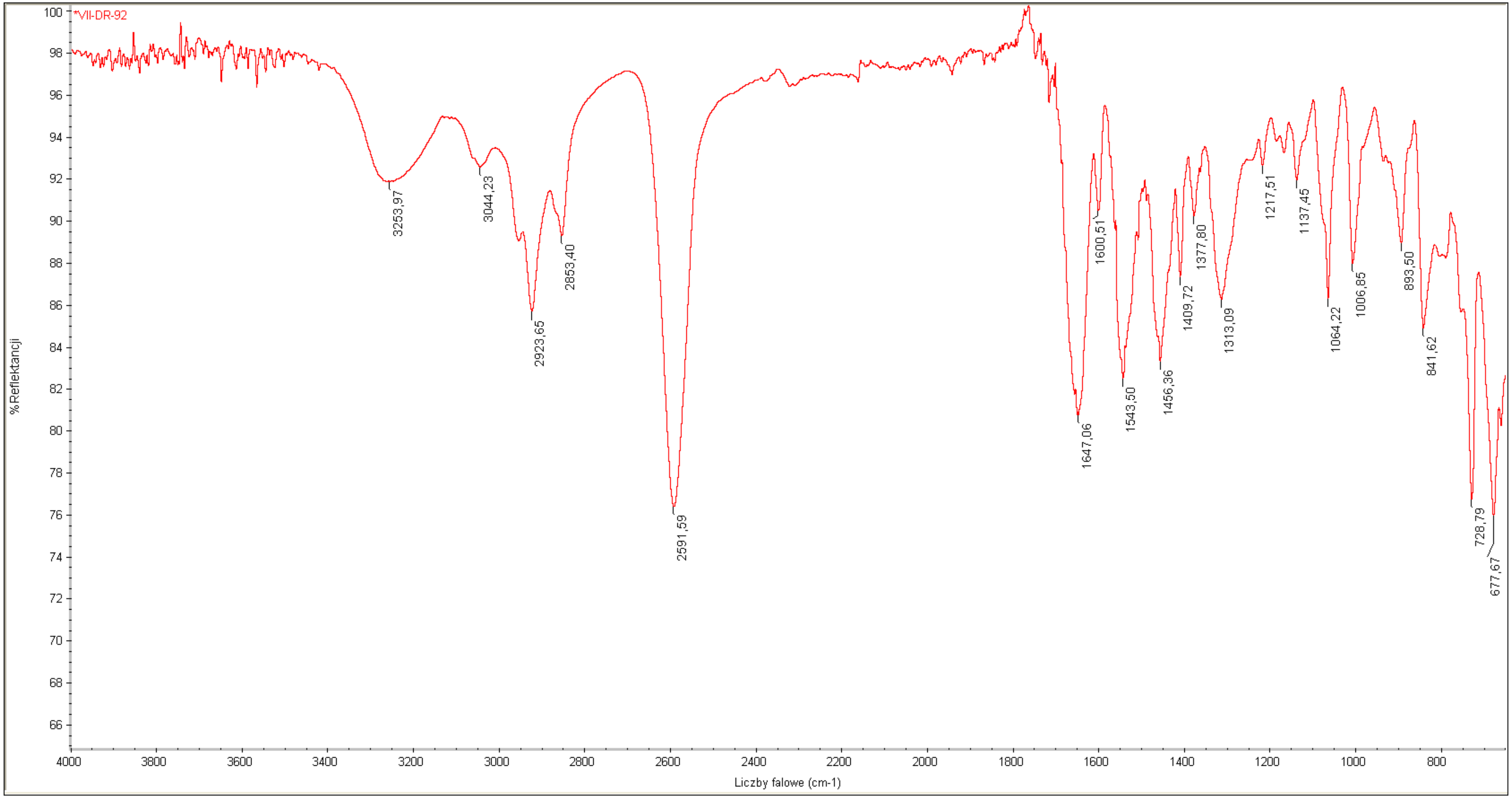

Figure S69. IR spectrum of compound **24**.

Spectrum Name: VI-DR-92_frg_dod
Start Ion: 200
End Ion: 450
Source: APCI + 10.0µA 250C
Capillary: 150V 200C Offset: 15V Span: 0V

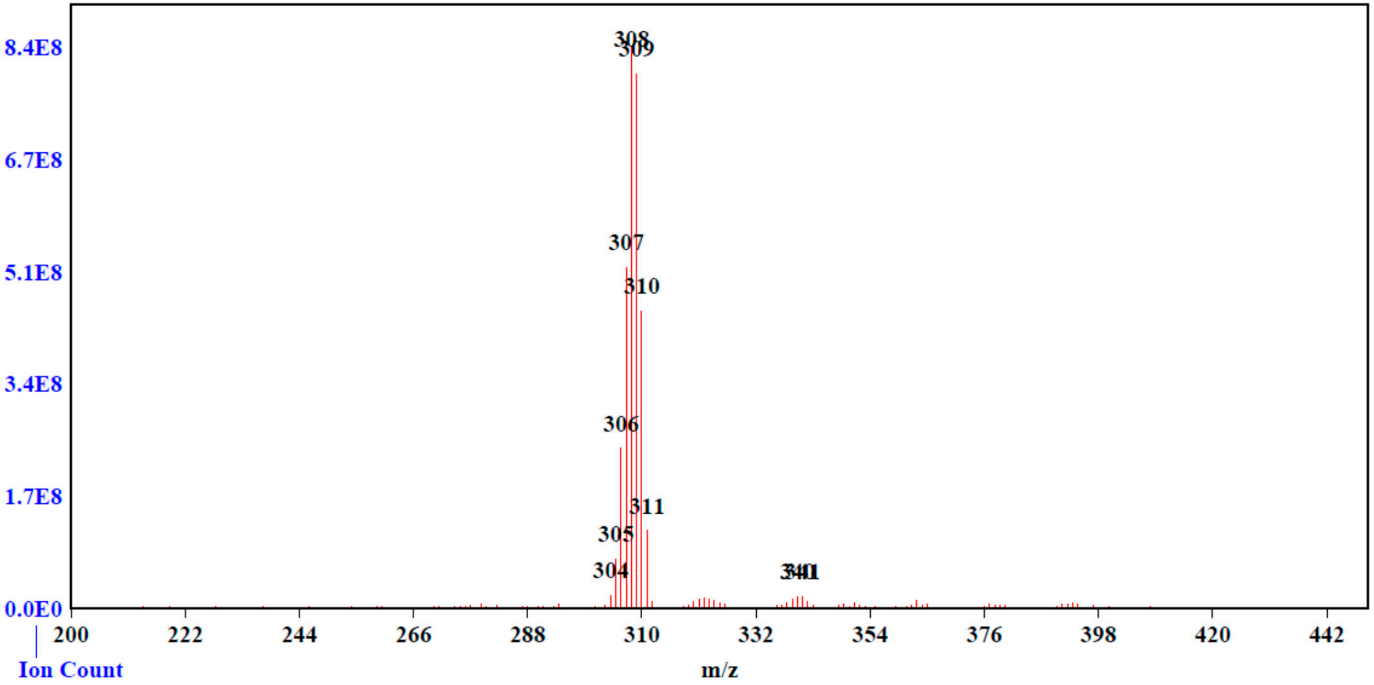

Figure S70. MS spectrum of compound **24**.

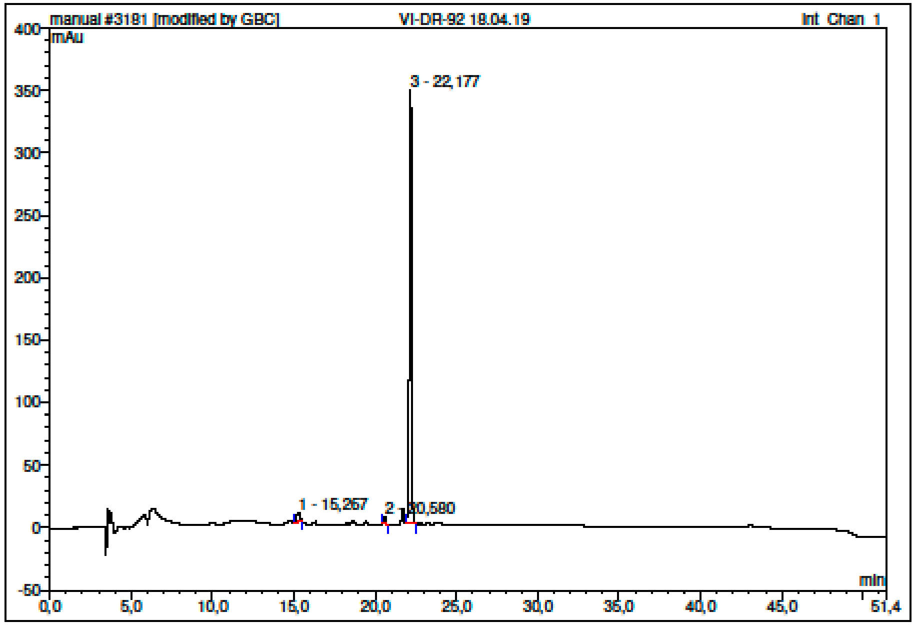

No.	Ret.Time min	Peak Name	Height mAu	Area mAu*min	Rel.Area %	Amount	Type
1	15,27	n.a.	7,034	1,867	3,07	n.a.	BMB*
2	20,58	n.a.	5,940	0,800	1,32	n.a.	BMB*
3	22,18	n.a.	347,519	58,052	95,61	n.a.	BMB
Total:			360,492	60,720	100,00	0,000	

Figure S71. HPLC analysis of compound **24**.

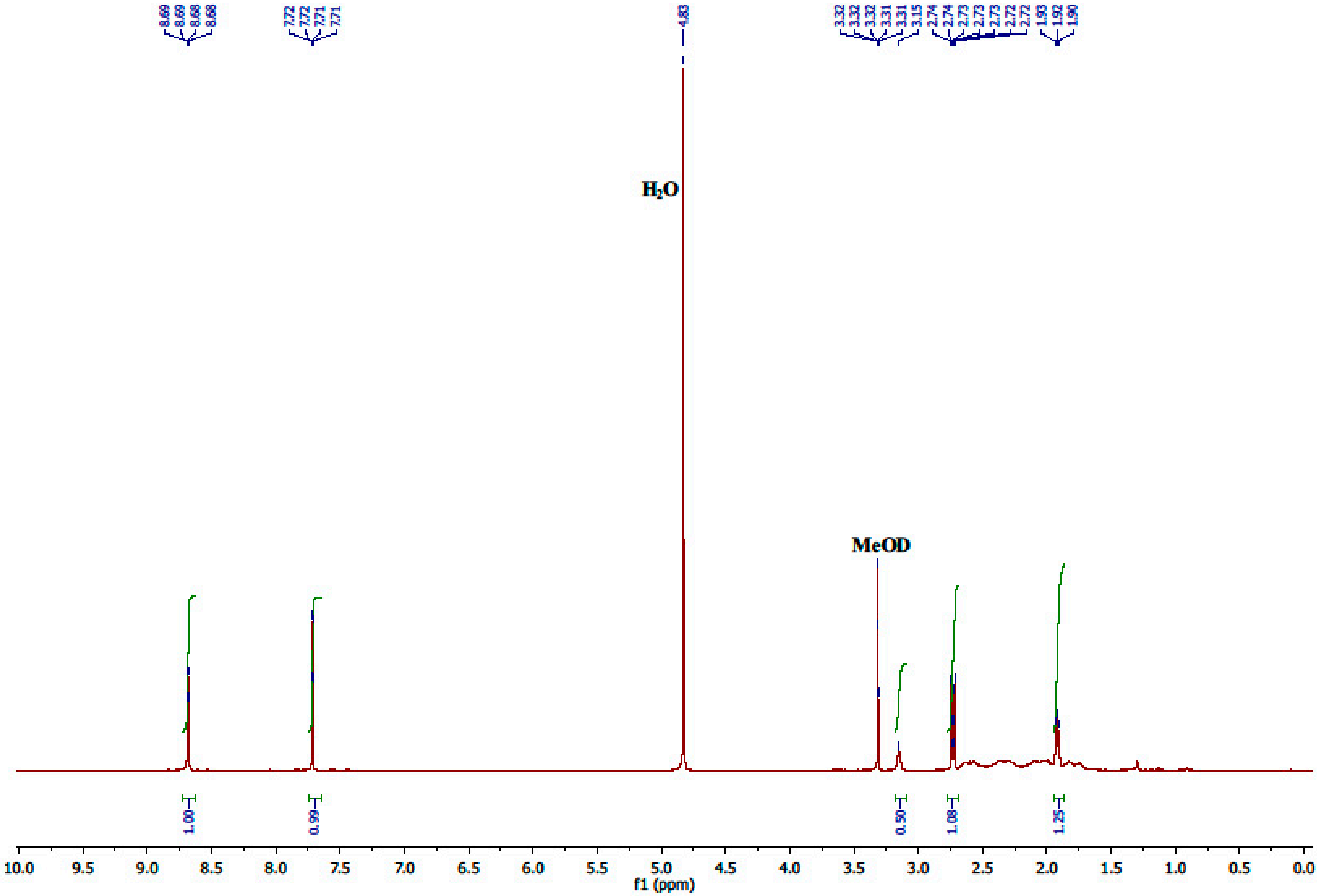

Figure S72. ^{1}H NMR spectrum of compound **25**.

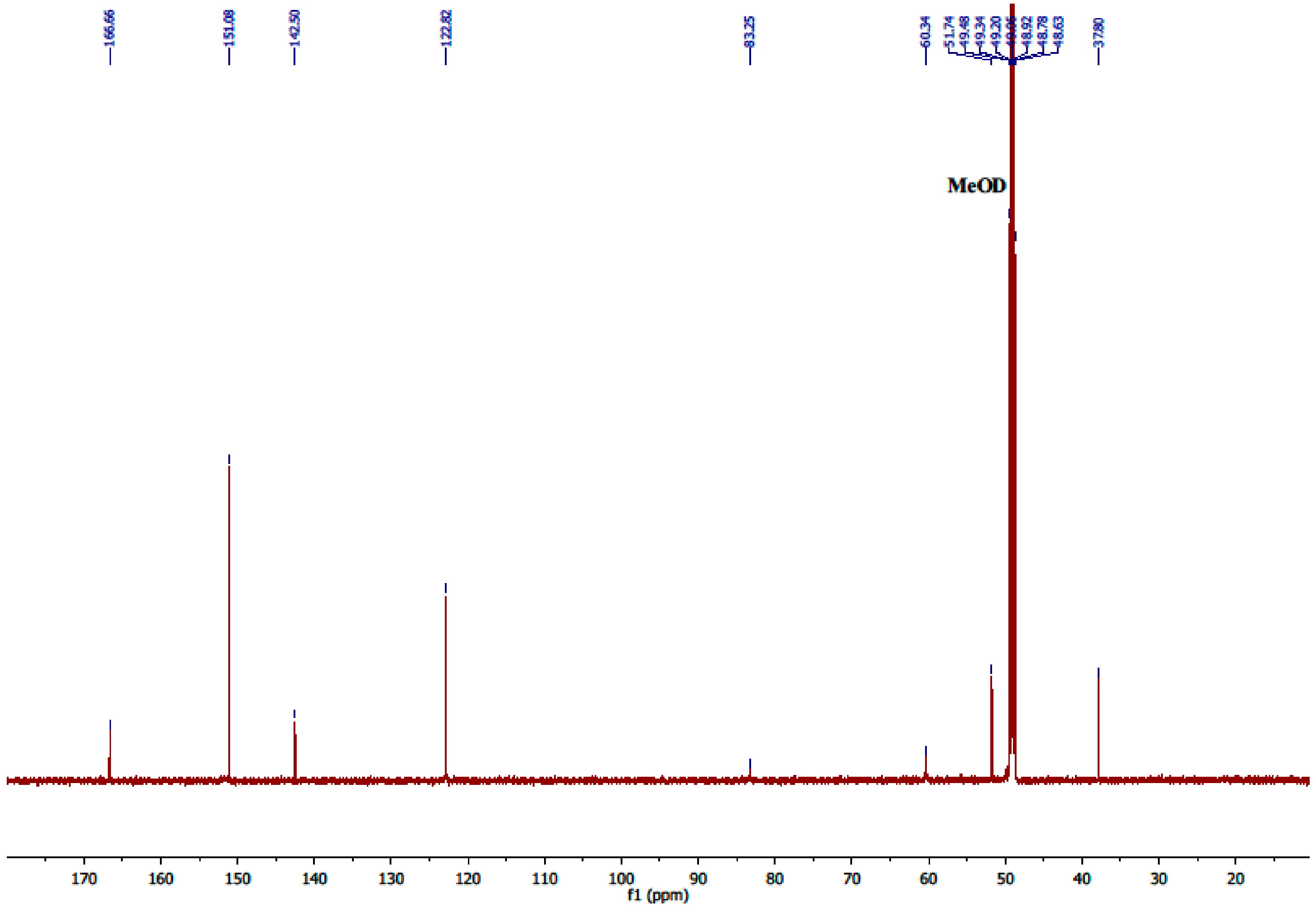

Figure S73. ^{13}C NMR spectrum of compound **25**.

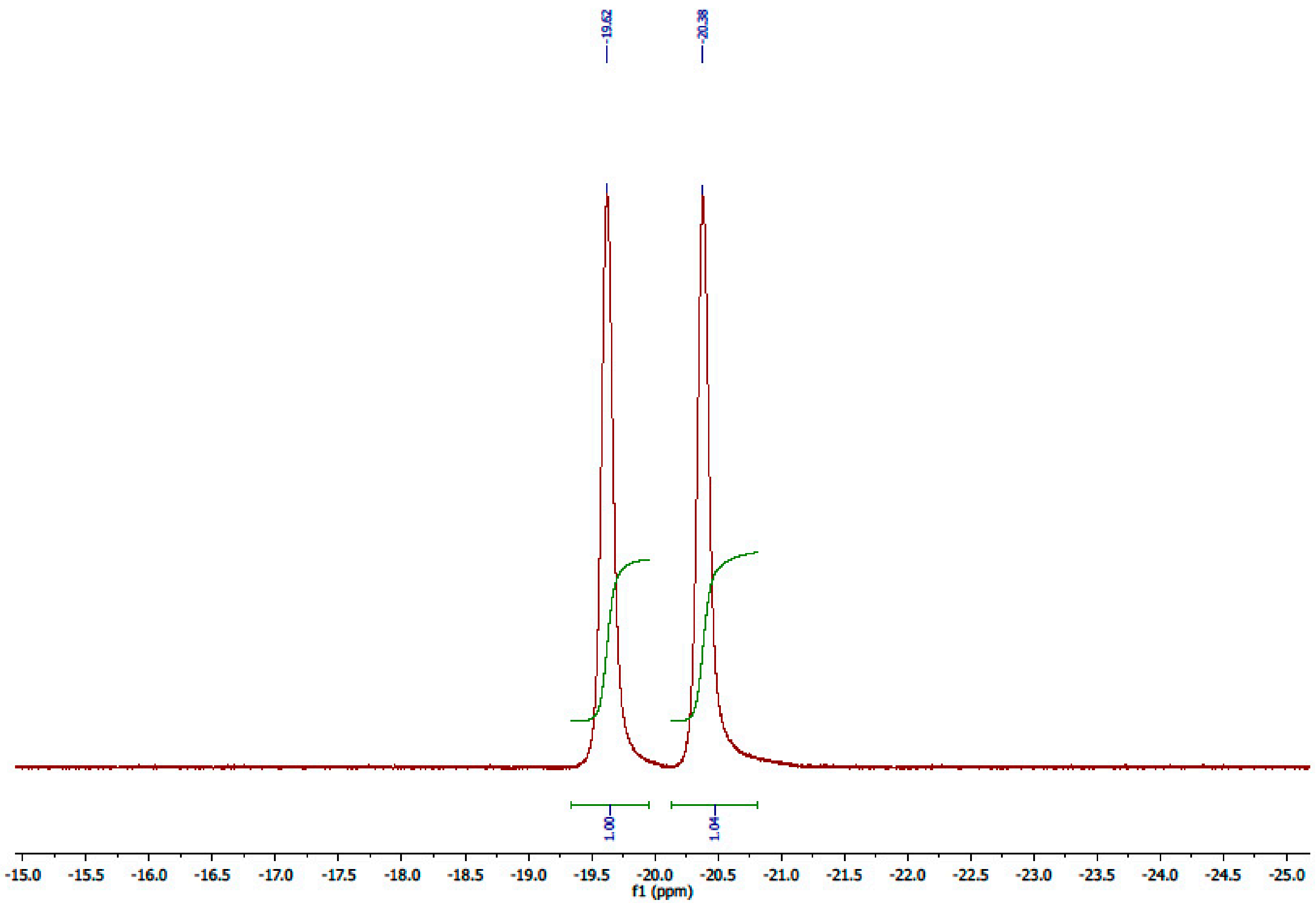

Figure S74. ^{11}B NMR {^{1}H BB} spectrum of compound **25**.

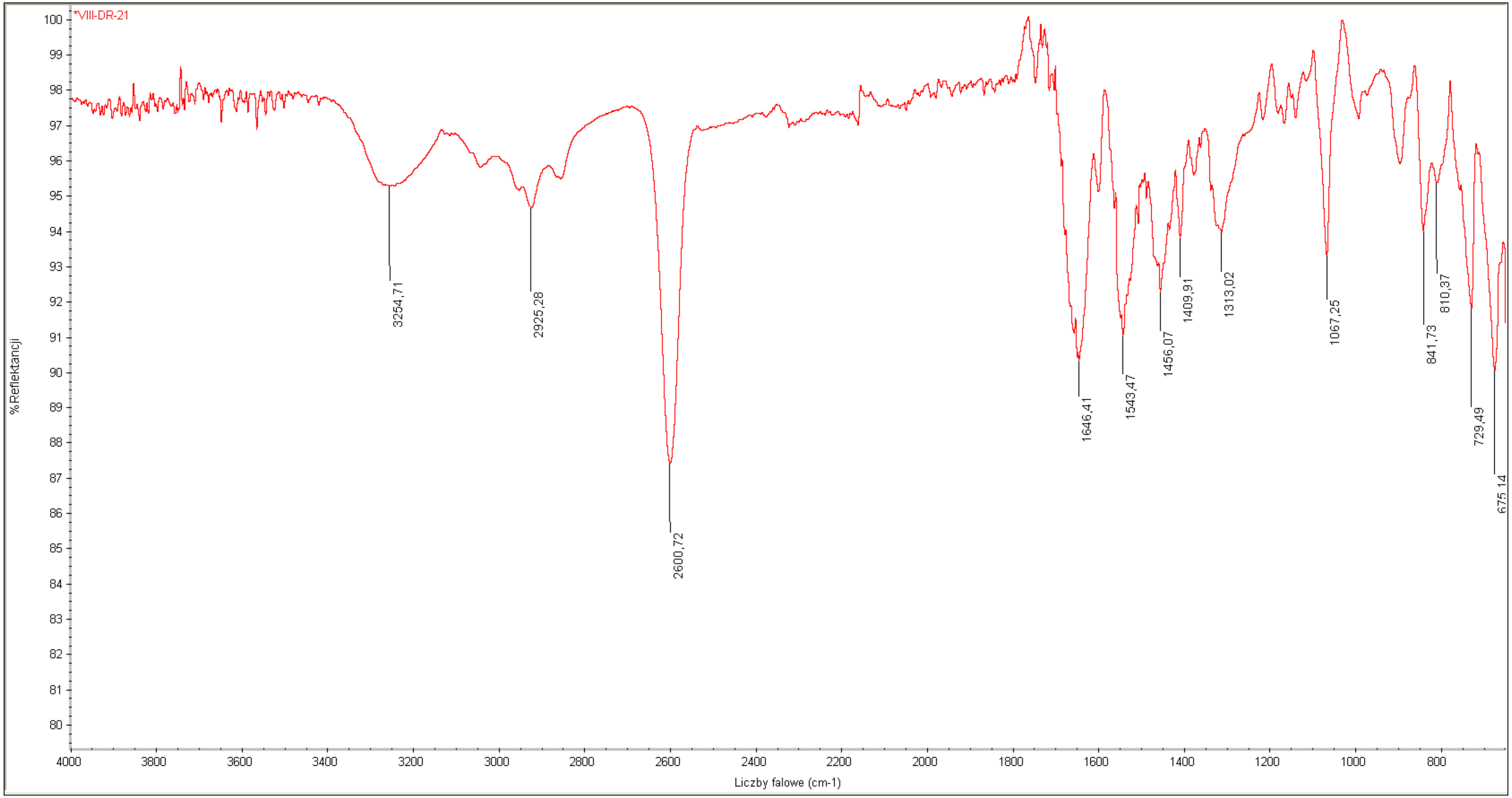

Figure S75. IR spectrum of compound **25**.

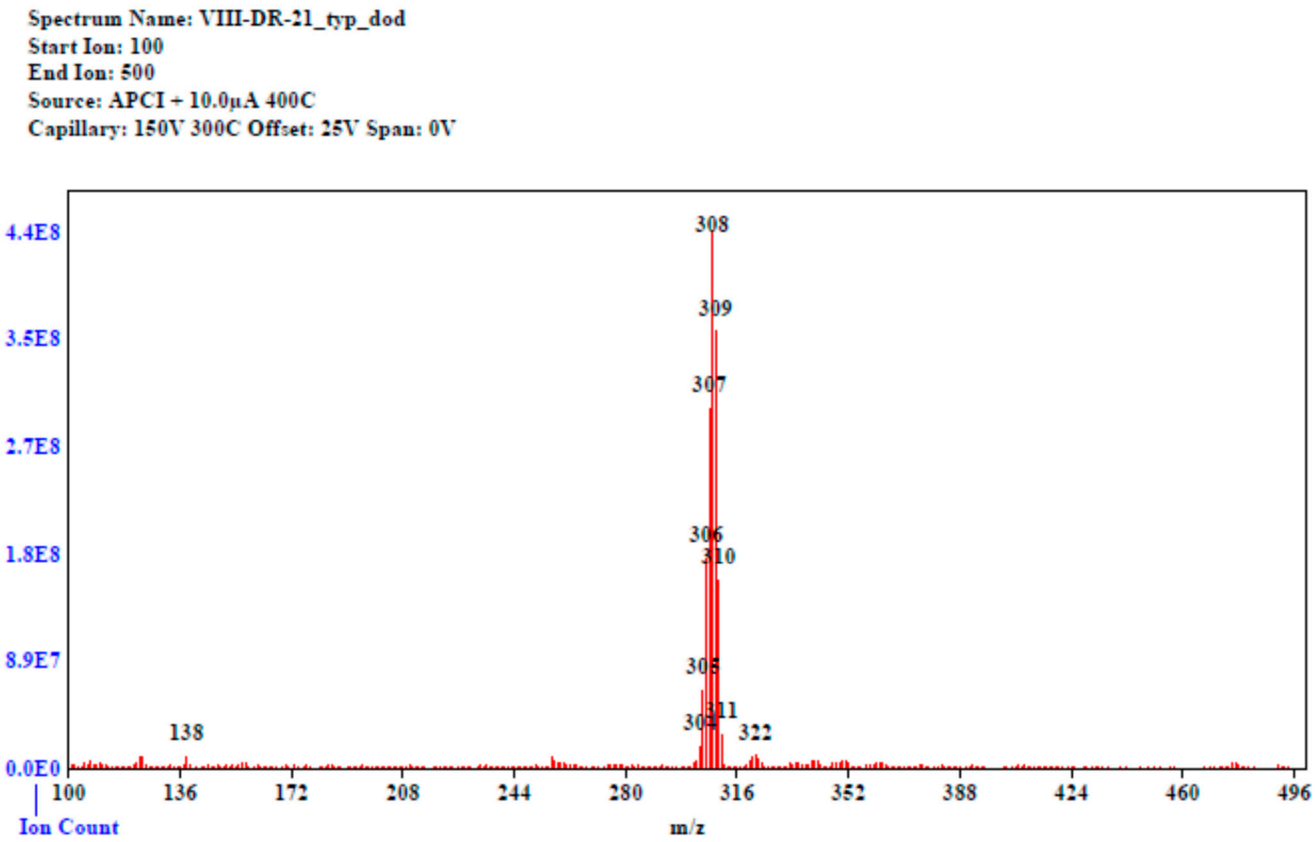

Figure S76. MS spectrum of compound **25**.

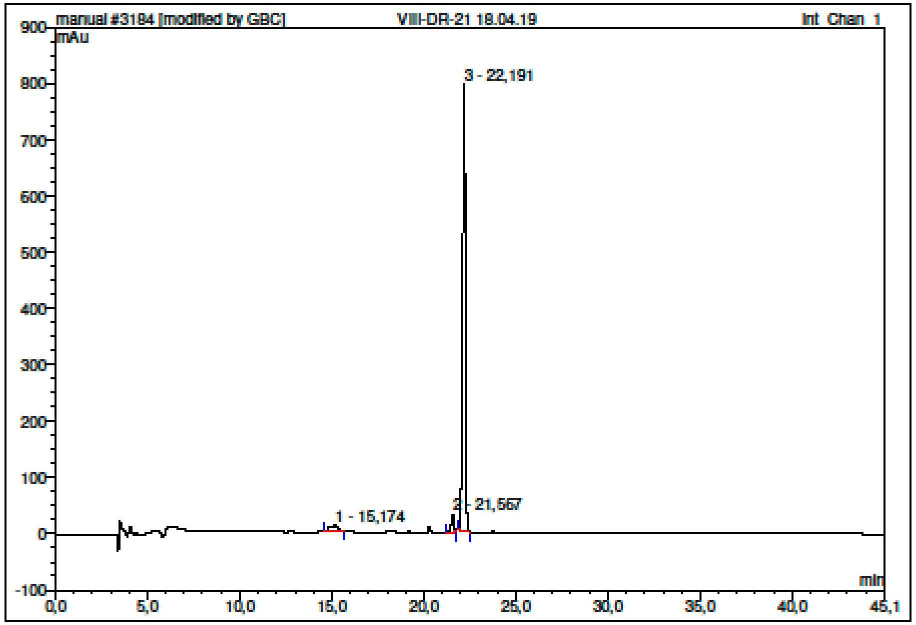

No.	Ret.Time min	Peak Name	Height mAu	Area mAu*min	Rel.Area %	Amount	Type
1	15,17	n.a.	9,692	5,308	3,54	n.a.	BMB
2	21,57	n.a.	31,846	5,170	3,45	n.a.	BMB
3	22,19	n.a.	794,425	139,310	93,00	n.a.	BMB
Total:			835,962	149,788	100,00	0,000	

Figure S77. HPLC analysis of compound **25**.

Table S1. Crystallographic data.

Compound	14	15	21
Wavelength [Å]	0.71073 (Mo Kα)	0.71073 (Mo Kα)	1.54184 (Cu Kα)
Temperature [K]	132	131	130
Space group	Pbcn	$P2_1/c$	C2/c
Z	8	4	8
a [Å]	20.6123(11)	10.9141(5)	21.3660(4)
b [Å]	7.7069(4)	20.0103(9)	6.47800(10)
c [Å]	20.7512(9)	7.6763(5)	27.1899(5)
β [°]	90	108.904(15)	103.552(2)
R_{int}	0.0565	0.0331	0.0295
Resolution [Å]	0.77	0.77	0.79
% completeness	94	90.3	98
Independent reflections	3522	3239	3772
R/R(for F_o>4σ)	0.0890/0.0556	0.0502/0.0429	0.0482/0.0443
CSD code	2012276	2012274	2012277

General procedure for competitive trapping

Compound (1.45-3.45 mg, 4.7-11.4 μmol), TEMPO (1 eq.), and Mn catalyst ($[Mn^{IV}\text{-}Mn^{IV}(\mu\text{-}O)_3L_2](PF_6)_2$ (L = 1,4,7-trimethyl-1,4,7-triazacyclononane) (6×10^{-3} mmol, 1.6 mol%) was dissolved in degassed ($Ar_{(g)}$) solution of MeOH/ACN (1:99, v/v). Periodic acid (2 eq.) was added slowly and the reaction mixture stirred for further 15 min. The solvent was removed and the reaction was quenched by H_2SO_4 (1 M, 20-25 uL), potassium bicarbonate (0.5 M, 20-25 uL) and saturated sodium sulfite solution (20-25 uL). The mixture was made alkaline with sodium carbonate and extracted with dichloromethane (3×0.1 mL). The organic phase was separated, dried over $MgSO_4$, filtered and evaporated to dryness. Then, the product was purified by column chromatography on silica gel (230-400 mesh) using a gradient elution from 0 to 10% MeOH in CH_2Cl_2 to afford pure product.

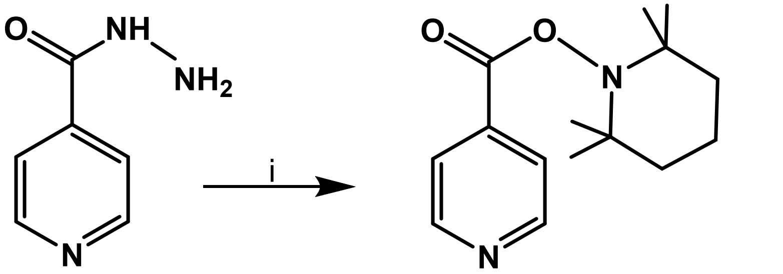

INH (**4**) *O*-isonicotinoylhydroxylamine (**26**)

Figure S78. The resulting trapped product *O*-isonicotinoylhydroxylamine (**26**): i) TEMPO, MeOH/ACN (1:99, v/v), $[Mn^{IV}\text{-}Mn^{IV}(\mu\text{-}O)_3L_2](PF_6)_2$ (L=1,4,7-trimethyl-1,4,7-triazacyclononane)/H_5IO_6.

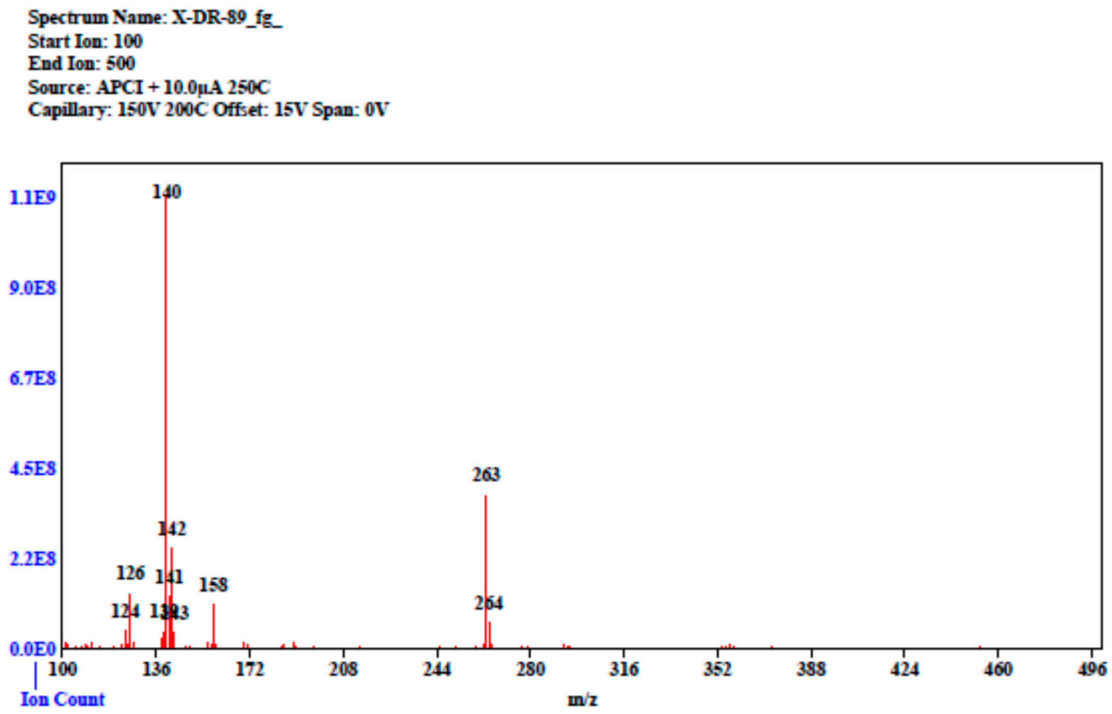

Figure S79. MS spectrum of reaction of INH with TEMPO, and manganese catalyst: APCI-MS: *m/z* 263, calcd for $C_{15}H_{22}N_2O_2$ = 262 (product **26**).

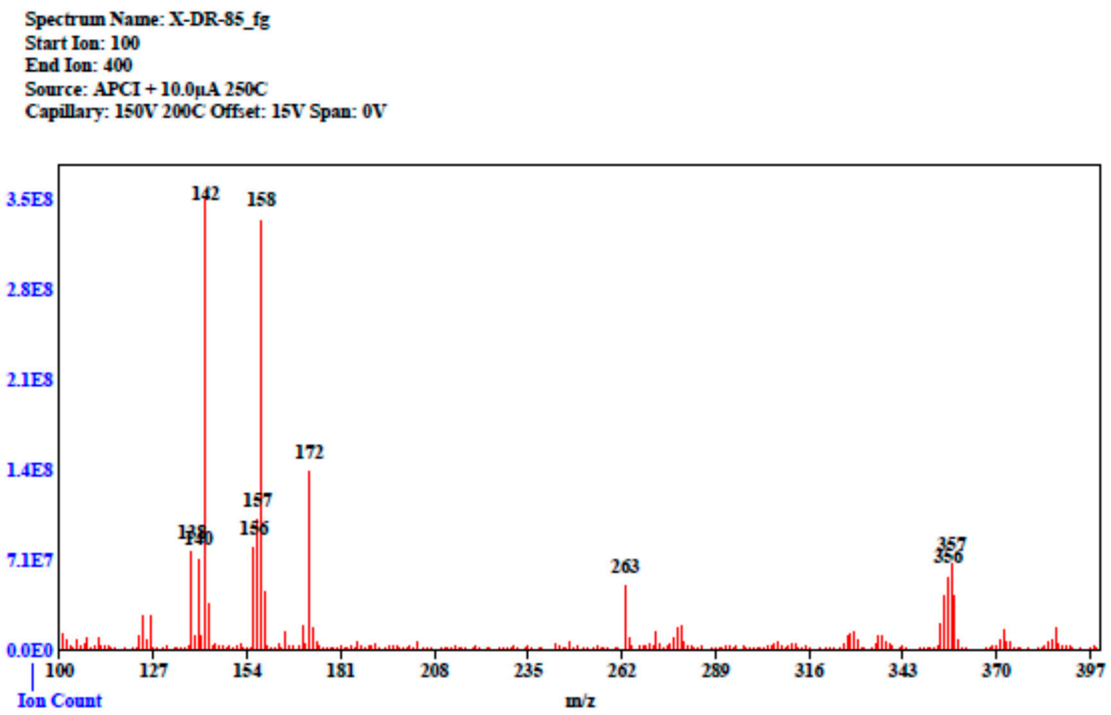

Figure S80. MS spectrum of reaction of **8** with TEMPO, and manganese catalyst.

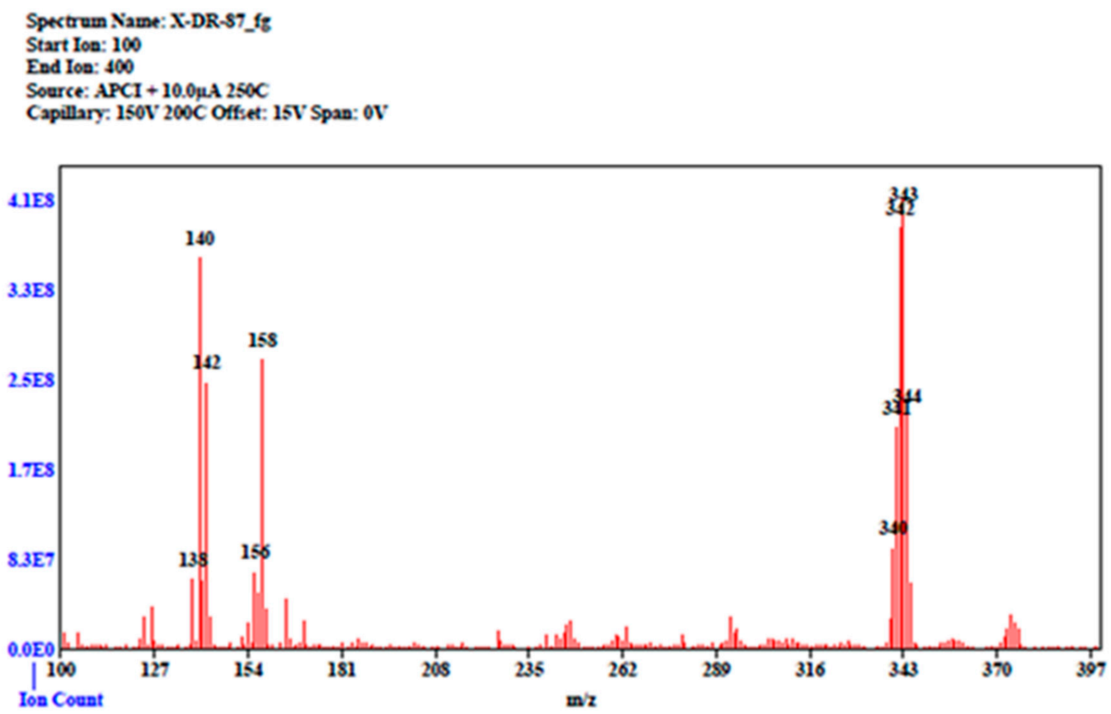

Figure S81. MS spectrum of reaction of **21** with TEMPO, and manganese catalyst.

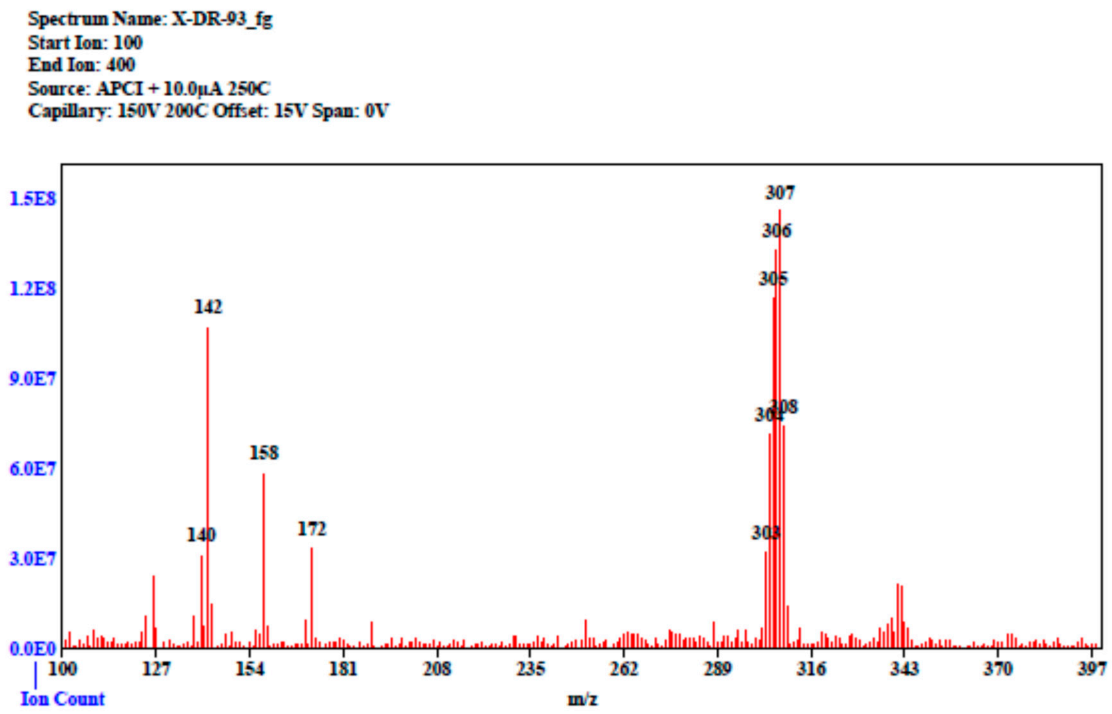

Figure S82. MS spectrum of reaction of **25** with TEMPO, and manganese catalyst.

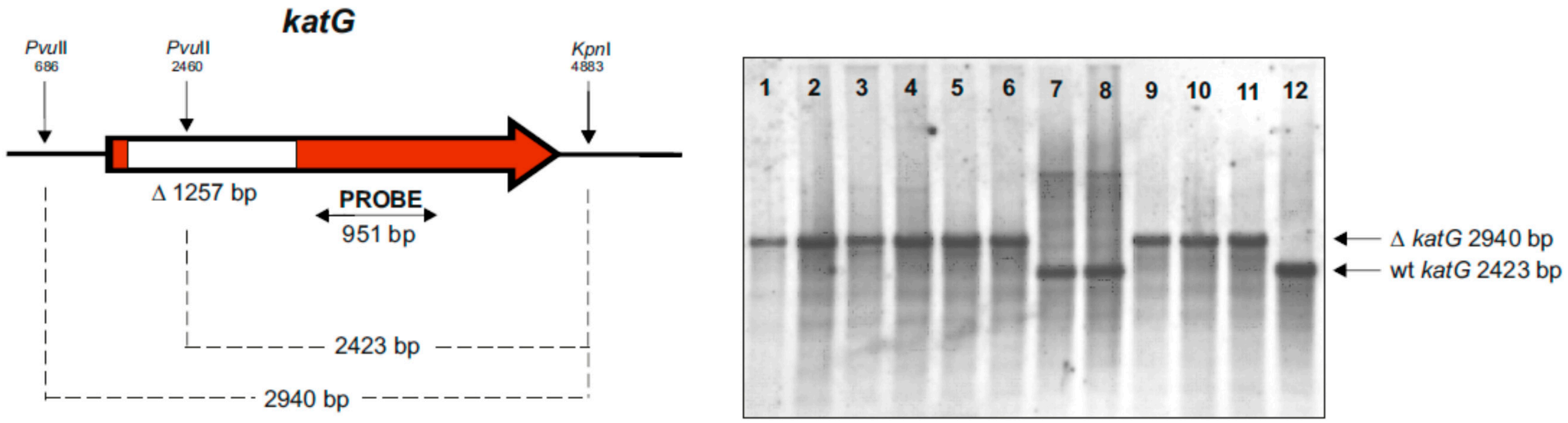

Figure S83. Confirmation of mutant construction by Southern blotting analysis. The *katG* was presented as red arrow. Restriction sites and internal deletion fragments (white rectangle) are indicated. DNA was digested with PvuII and KpnI restrictions enzymes. Lanes 1-6, 9-11 – *M. tuberculosis* H37Rv Δ*katG* DCO (double cross-over mutants), lanes 7-8 – *M. tuberculosis* H37Rv SCO (single cross-over mutants), lane 12 – *M. tuberculosis* H37Rv wild-type strain.
